# Diterpenoids from *Blumea balsamifera* and Their Anti-Inflammatory Activities

**DOI:** 10.3390/molecules27092890

**Published:** 2022-04-30

**Authors:** Xiao-Ling Huang, Dai-Wei Wang, Ying-Qian Liu, Yong-Xian Cheng

**Affiliations:** 1 School of Pharmacy, Lanzhou University, Lanzhou 730000, China; huangxl19@lzu.edu.cn; 2 Institute for Inheritance-Based Innovation of Chinese Medicine, School of Pharmaceutical Sciences, Health Science Center, Shenzhen University, Shenzhen 518060, China; dweiwang@foxmail.com; 3 Guangdong Key Laboratory of Functional Substances in Medicinal Edible Resources and HealthcareProducts, School of Life Sciences and Food Engineering, Hanshan Normal University, Chaozhou 521041, China

**Keywords:** *Blumea balsamifera*, diterpenoid, anti-inflammation

## Abstract

Six new diterpenoids, blusamiferoids A–F (**1**–**6**), including four pimarane-type diterpenoids, one rosane-type diterpenoid (**3**), and one rearranged abietane-type diterpenoid (**6**), were isolated from the dry aerial parts of *Blumea balsamifera.* Their structures were characterized by spectroscopic and computational methods. In particular, the structures of **1** and **4** were confirmed by X-ray crystallography. Compounds **5** and **6** were found to dose-dependently inhibit the production of TNF-α, IL-6, and nitrite oxide, and compound **5** also downregulated NF-*κ*B phosphorylation in lipopolysaccharide (LPS)-induced RAW 264.7 cells.

## 1. Introduction

*Blumea balsamifera* (L.) DC. (Asteraceae), also named sambong, is a perennial herbaceous plant and a traditional herb, which is commonly found in Southeast Asia, such as China, Malaysia, Thailand, and the Philippines [1]. As a traditional medicine, the whole plants or leaves of *B. balsamifera* were widely used to treat a cough, urinary tract infection, gastric ulcer, headache, fever, rheumatism, and menstrual diseases [2,3]. In addition, *B. balsamifera* is an important plant source of l-borneol, which was designated as the only natural source of *Aipian* by the Pharmacopoeia of the PR of China [4]. The importance of *B. balsamifera* in traditional herbs has aroused widespread interest over the past decades. Previous phytochemical investigations revealed that *B. balsamifera* contains several types of chemicals such as volatile oils, flavonoids, and terpenoids [5]. Pharmacological research has disclosed that the whole plants, crude extracts, and isolated constituents of *B. balsamifera* contain several biological capacities such as wound healing [6], anti-cancer [7], anti-bacterial [8], anti-inflammatory [9], anti-oxidant [10], and anti-influenza virus activities [11].

As a common folk herb, *B. balsamifera* is often used to treat rheumatoid arthritis, dermatitis, and colds [12], indicating its anti-inflammatory activity. It was found that the volatile oil of *B. balsamifera* had a significant anti-inflammatory effect in inflammatory mice [9,13]. It has been reported that non-volatile components of *B. balsamifera*, such as the ethanol extract and the residue after extraction of the volatile oil, also have a certain inhibitory effect on inflammation, indicating that the non-volatile part of *B. balsamifera* still has its utilization value [14,15]. However, there are few related reports and it is necessary to further study this part. To further study the non-volatile components of *B. balsamifera* and their anti-inflammatory activities, we have carried out research on the ethyl acetate fraction of a 95% ethanol extract of *B. balsamifera*, resulting in the isolation of four new pimarane-type diterpenoids, one rosane-type diterpenoid, and a rearranged abietane-type diterpenoid. In this paper, we report their isolation, structural characterization, and anti-inflammatory activity evaluation.

## 2. Results and Discussion

### 2.1. Structure Elucidation of the Compounds

**Figure 1:**
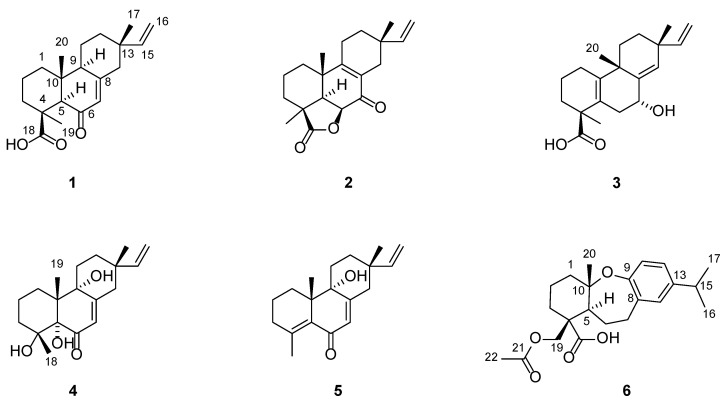
The structures of compounds **1**–**6** from *B. balsamifera*.

**Figure 2:**
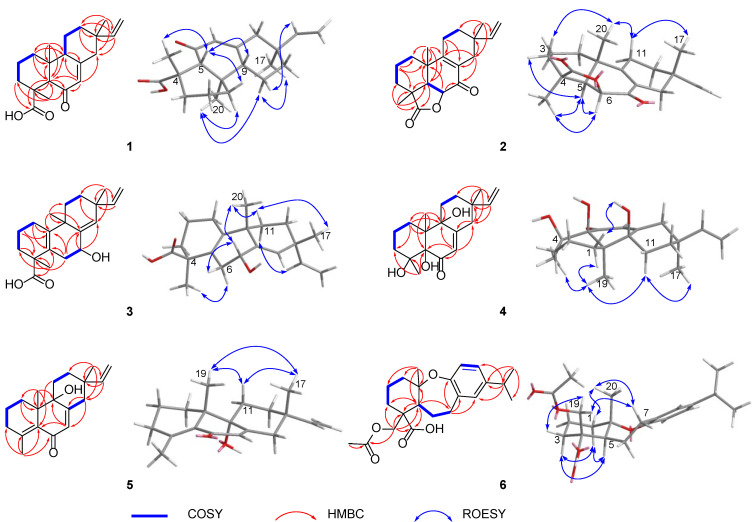
Key 2D NMR correlations of **1**–**6**.

**Figure 3:**
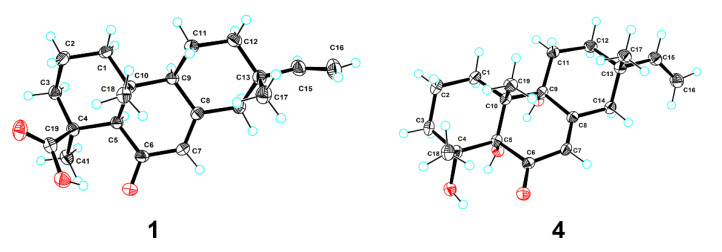
Plot of X-ray crystallographic data for compounds **1** (**left**) and **4** (**right**). Displacement ellipsoids are drawn at the 50% probability level.

Blusamiferoid A (**1**) was obtained as colorless small quadrate crystals. Its molecular formula C_20_H_28_O_3_ was deduced on the basis of the positive HRESIMS at *m/z* 339.1922 [M + Na]^+^ (calculated for C_20_H_28_O_3_Na 339.1931), ^13^C NMR, and DEPT spectra, indicating seven degrees of unsaturation (Supplementary Materials). In the ^1^H NMR spectrum, three methyl signals (*δ*_H_ 0.88, s; 0.91, s; 1.42, s), a typical ABX system of a vinyl group (*δ*_H_ 4.96, dd, *J* = 10.8, 0.9 Hz; *δ*_H_ 4.99, dd, *J* = 17.5, 0.9 Hz, and *δ*_H_ 5.81, dd, *J* = 17.5, 10.8 Hz), an olefinic proton signal (*δ*_H_ 5.92, brs), and a carboxylic acid proton (*δ*_H_ 12.45, brs) are observed (Table 1). The ^13^C NMR and DEPT spectra (Table 1) show 20 signals attributed to three methyls, seven methylenes (one sp^2^ and six sp^3^), four methines (two sp^2^ and two sp^3^), and six nonprotonated carbons (including one keto-carbonyl, one carboxylic carbonyl, and one olefinic). Analyses of the 1D and 2D NMR spectra and comparison with the literature suggest that compound **1** possesses a similar structure to 6*β*-hydroxyisopimaric acid [16]. The differences between them are the presence of one carbonyl group at C-6 (*δ*_C_ 205.9) in **1** rather than a hydroxy group, which is confirmed by the HMBC correlations of H_3_-19/C-6, H-5/C-6, and H-7 (*δ*_H_ 5.92)/C-6, as well as the chemical shifts of C-8 (*δ*_C_ 164.7). Thus, the planar structure of **1** was assigned (Figure 1). The relative configuration of **1** was determined by ROESY data (Figure 2), the correlations of H_3_-20/Ha-1, Hb-1/H-5, H_3_-19/H-5, and H-5/H-9 imply that H_3_-20, Ha-1 are on the same face of the bicyclic ring, while H_3_-19, H-5, H-9, and Hb-1 are on the other side. Likewise, ROESY correlations of H_3_-20/Hb-11, H_3_-17/Hb-11, H-15/Ha-11 suggest that H_3_-17 and H_3_-20 are on the same side. Thus, the relative configuration of **1** was defined. As for the absolute configuration of **1**, it was assigned by X-ray diffraction analysis with CuKα radiation. The results show the absolute configuration of **1** as 4*S*,5*R*,9*S*,10*R*,13*S* with a calculated Flack parameter of −0.03 (5) (Figure 3). Hence, the structure of **1** was ultimately determined.

Blusamiferoid B (**2**), obtained as white solids, has the molecular formula C_20_H_26_O_3,_ as deduced from its HRESIMS, ^13^C NMR, and DEPT spectra (eight degrees of unsaturation). The ^1^H NMR spectrum of **2** (Table 1) indicates the presence of three methyl signals (*δ*_H_ 0.98, s; 1.09, s; 1.32, s), a typical ABX system of a vinyl group (*δ*_H_ 4.89, dd, *J* = 17.5, 1.1 Hz; *δ*_H_ 4.95, dd, *J* = 10.7, 1.1 Hz, and *δ*_H_ 5.74, dd, *J* = 17.5, 10.7 Hz). The ^13^C NMR and DEPT spectra (Table 1) show 20 signals attributed to three methyls, seven methylenes (one sp^2^ and six sp^3^), three methines (one sp^2^ and two sp^3^), and seven nonprotonated carbons (including one keto-carbonyl, one ester carbonyl, and two olefinic carbons). Analysis of its ^1^H and ^13^C NMR data suggests that **2** belongs to a pimarane skeleton. Comparing the NMR data of dabeshanensin B [17] with those of **2** indicates that **2** might be an analogue of dabeshanensin B with a missing double bond at C-5 and C-6, which is confirmed by the ^1^H-^1^H COSY correlation of H-5/H-6 and HMBC correlations of H_3_-19/C-5, C-6, H_3_-20/C-5, H-5/C-4, C-10, C-18 (*δ*_C_ 180.6), and H-6 (*δ*_H_ 4.74)/C-10, C-7 (*δ*_C_ 192.1) (Figure 2). Therefore, the planar structure of **2** was established.

**Figure 4:**
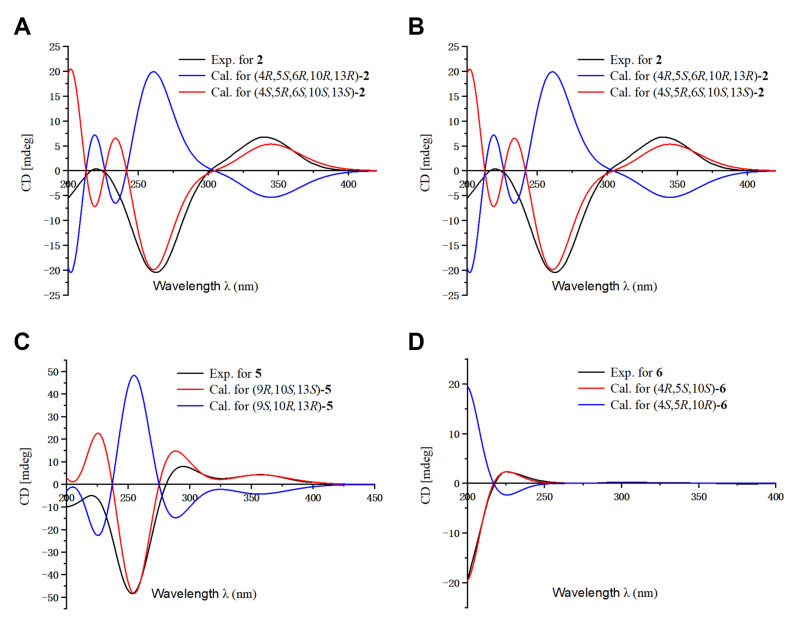
Comparison of the calculated ECD and experimental spectra in MeOH. (**A**) The calculated ECD spectra of (4*S*,5*R*,6*S*,10*S*,13*S*)-**2** and (4*R*,5*S*,6*R*,10*R*,13*R*)-**2** at B3LYP/6-31G level, σ = 0.30 eV; shift = 2 nm. (**B**) The calculated ECD spectra of (4*S*,7*R*,9*S*,13*R*)-**3** and (4*R*,7*S*,9*R*,13*S*)-**3** at B3LYP/6-31G level, σ = 0.30 eV; shift = −15 nm. (**C**) The calculated ECD spectra of (9*R*,10*S*,13*S*)-**5** and (9*S*,10*R*,13*R*)-**5** at B3LYP/6-31G level, σ = 0.30 eV; shift = −10 nm. (**D**) The calculated ECD spectra of (4*R*,5*S*,10*S)-***6** and (4*S*,5*R*,10*R)-***6** at B3LYP/6-31G level, σ = 0.30 eV; shift = −18 nm.

The relative configuration of **2** was determined by analysis of its ROESY spectrum (Figure 2). The ROESY correlations of H_3_-19/H-5, H-6; H-5/H-6, Hb-3, indicate they are on the same side, while H_3_-20 is on the opposite side for the correlation between H_3_-20 and Ha-3. Meanwhile, the ROESY correlations of H_3_-20/Hb-11 and H_3_-17/Hb-11 indicate they are cofacial. Hence, the relative configuration of **2** was assigned. The absolute stereochemistry of **2** was further clarified by comparison of the experimental electronic circular dichroism (ECD) spectrum of **2** with the calculated spectra of (4*S*,5*R*,6*S*,10*S*,13*S*)-**2** and (4*R*,5*S*,6*R*,10*R*,13*R*)-**2**. It was found that the calculated ECD spectrum of (4*S*,5*R*,6*S*,10*S*,13*S*)-**2** agrees well with the experimental spectrum of **2** (Figure 4), showing the absolute configuration of **2** to be 4*S*,5*R*,6*S*,10*S*,13*S*.

Blusamiferane C (**3**), separated as yellowish solids, has the molecular formula C_20_H_28_O_3_ derived from its HRESIMS, ^13^C NMR, and DEPT spectra, having seven degrees of unsaturation. Comparison of the NMR data of **3** with those of engleromycenolic acid [18], reveals that **3** might be a rosane-type diterpene. The resonances at *δ*_C_ 145.5 and *δ*_C_ 120.3 in the ^13^C NMR spectrum of **3** suggest that there is an additional double bond in **3**. The HMBC correlations of H_3_-17/C-14 (*δ*_C_ 120.3), H-15/C-14, H_3_-20/C-8 (*δ*_C_ 145.5), indicate that the new double bond is located between C-8 and C-14. In addition, the HMBC correlations of H-7 (*δ*_H_ 4.46)/C-8, C-9, C-14, as well as the ^1^H-^1^H COSY correlation of H-6/H-7 indicate the hydroxyl is attached to C-7 instead of C-2. Hence, the planar structure of **3** was established. On the basis of the analysis of the ROESY spectrum (Figure 2), the correlations of H_3_-20/H-7, Ha-11; H_3_-17/Ha-11, and H-15/Hb-11 indicate that H_3_-20, H_3_-17, and H-7 are on the same side of the ring system. The strong ROESY correlations of H_3_-19/Hb-6, and the coupling constant of H-7 (*J*_H-7,Hb-6_ = 10.6 Hz, *J*_H-7,Ha-6_ = 5.7 Hz) allow us to conclude that H_3_-19 and H-7 are at the opposite orientation. On the basis of these results, the absolute configuration of **3** was subsequently assigned by direct ECD calculations of (4*S*,7*R*,9*S*,13*R*)-**3** and (4*R*,7*S*,9*R*,13*S*)-**3**. It is evident that the ECD curve of (4*S*,7*R*,9*S*,13*R*)-**3** matches well with the experimental curve (Figure 4), suggesting that the configuration of (4*S*,7*R*,9*S*,13*R*)-**3** is more reasonable. As a result, the absolute configuration of **3** was clarified as 4*S*,7*R*,9*S*,13*R*.

Blusamiferoid D (**4**), obtained as colorless small quadrate crystals, has the molecular formula C_19_H_28_O_4_, as deduced from its HRESIMS, ^13^C NMR, and DEPT spectra (six degrees of unsaturation). The 1D NMR spectra of **4** exhibits a pattern analogous to that of **1**. The differences between **1** and **4** are the presence of three additional hydroxy groups located at C-4 (*δ*_C_ 74.4), C-5 (*δ*_C_ 80.2), C-9 (*δ*_C_ 74.0), and the absence of one carboxylic acid at C-4 in **4** on the basis of the HMBC correlations of H_3_-19/C-5, C-9, H_3_-18/C-4, C-5, and H-7/C-5, C-9. The relative configuration of **4** was assigned by ROESY evidence. The ROESY correlations (Figure 2) of H_3_-19/H_3_-18, Ha-11, Hb-1; 9-OH/Ha-1, and H_3_-17/Ha-11 are observed, indicating that three methyls are on the same side, while 9-OH is on the opposite side. Through analysis of the molecular model, we found that the ROESY correlation of H_3_-19/H_3_-18 can only be observed when H_3_-19 and 5-OH are on the opposite side, thus confirming the relative configuration of 5-OH. This conclusion was also secured by subsequent X-ray diffraction analysis using CuKα radiation, allowing us to assign the absolute configuration of **4** as 4*R*,5*S*,9*R*,10*R*,13*S* with a calculated Flack parameter of 0.01 (3) (Figure 3).

Blusamiferoid E (**5**) was isolated as yellowish gums. Its molecular formula was deduced as C_19_H_26_O_2_ by analysis of the HRESIMS (*m*/*z* 287.2005 [M + H]^+^, calculated for 287.2006), ^13^C NMR, and DEPT spectra (seven degrees of unsaturation). Through analysis of the 1D and 2D NMR data, it was noted that the presence of a double bond between C-4 and C-5 instead of two hydroxy groups in **5** are the main differences between **4** and **5**. In addition to the chemical shifts of C-4 (*δ*_C_ 149.4) and C-5 (*δ*_C_ 133.0), the HMBC correlations of H_3_-18/C-4, C-5, H_3_-19/C-5 further confirmed the general structure of **5** (Figure 1).

The relative configurations at the stereogenic centers in **5** were assigned by analysis of the ROESY spectrum (Figure 2), which shows correlations between H_3_-19/Hb-11, H_3_-17/Hb-11, H_3_-19/H_3_-17 (weak), indicating that H_3_-19, H_3_-17 are located on the same face. Through molecular model analysis, we found that the spacial interaction of H_3_-19/H_3_-17 can only be observed when H_3_-19 and 9-OH are on the opposite side. To confirm our conclusion from the molecular model study, NMR calculations to clarify the relative configuration at C-9 were carried out. The results disclose that **5** is likely the configuration of (9*R*,10*S*,13*S*)-**5** based on the DP4+ probability analysis (Figure S6) and the correlation coefficient (*R*^2^) (Figure S5). Thus, the relative configuration at C-9 was finalized. To assign the absolute configuration of **5**, ECD calculations on (9*R*,10*S*,13*S*)-**5** and (9*S*,10*R*,13*R*)-**5** were conducted. The results show that the ECD spectrum of the former enantiomer agrees well with the experimental spectrum of **5** (Figure 4), showing the absolute configuration of **5** to be 9*R*,10*S*,13*S*.

Blusamiferoid F (**6**), obtained as yellowish gums, has the molecular formula C_22_H_30_O_5_ as deduced from its HRESIMS (*m*/*z* 375.2165 [M + H]^+^, calculated for 375.2166), ^13^C NMR, and DEPT spectra (eight degrees of unsaturation). The 1D NMR data of **6** resemble those of karamatsuic acid [19] with the exception of the presence of an acetyl group (*δ*_H_ 2.04, H_3_-22, *δ*_C_ 20.8, C-22, and *δ*_C_ 170.9, C-21), which is confirmed by the HMBC correlations of H_3_-22/C-21 and H-19/C-3, C-4, C-5, C-18, C-21. Thus, the structure of **6** was defined (Figure 1). The relative configuration of three chiral centers of **6** was determined by analysis of ROESY spectrum. ROESY correlations (Figure 2) between H_3_-20/Ha-1, H-5/Hb-1, H-5/Ha-3 (in pyridine-*d*_5_), Hb-19/Hb-3 (in pyridine-*d*_5_), indicate H_3_-20 and Hb-19 are located on the same side of this ring system, while H-5 situates on the opposite side. Following that, we used ECD computations to determine its absolute configuration. The calculated ECD spectrum for (4*R*,5*S*,10*S*)-**6** fits the experimental spectrum well (Figure 4), allowing the absolute configuration of **6** to be assigned as 4*R*,5*S*,10*S*.

### 2.2. Biological Evaluation

**Figure 5:**
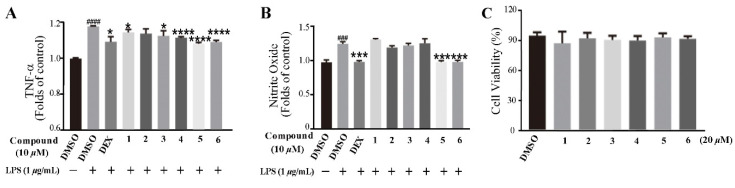
Compounds suppressed LPS-induced TNF-α and nitrite oxide expression in RAW 264.7 cells. (**A**) Compounds suppressed LPS-induced TNF-α expression in RAW 264.7 cells. The cells were pretreated with compounds for 2 h and then stimulated with 1 μg/mL LPS for 12 h. Culture media were collected to measure TNF-α concentration using ELISA kit. (**B**) Cells were treated with LPS with or without compounds for 24 h, the culture supernatants were collected and centrifuged. The production of nitrite oxide was measured using the Griess Kit. (**C**) RAW 264.7 cell proliferation in response to compounds. Data represent mean ± SEM values of three experiments. * *p* < 0.05, *** *p* < 0.001, and **** *p* < 0.0001 compared with LPS alone. ^###^ *p* < 0.001 and ^####^ *p* < 0.0001 compared with DMSO alone. Dexamethasone (DEX) (1 μM) was used as a positive control.

**Figure 6:**
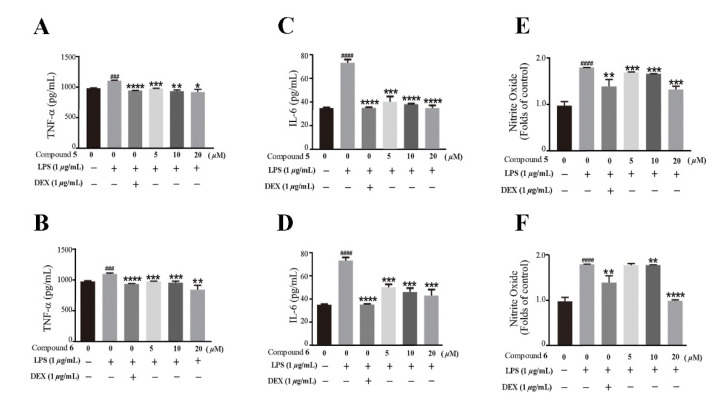
Compounds **5** and **6** suppressed pro-inflammatory expression in LPS-induced RAW 264.7 cells. (**A**,**B**) TNF-α in the supernatant were examined by an ELISA. (**C**,**D**) IL-6 in the supernatant were examined by an ELISA. (**E**,**F**) The production of nitrite oxide was measured using the Griess Kit. Data represent mean ± SEM values of three experiments. * *p* < 0.05, ** *p* < 0.01, *** *p* < 0.001 and **** *p* < 0.0001 compared with LPS alone. ^###^ *p* < 0.001 and ^####^ *p* < 0.0001 compared with DMSO alone. Dexamethasone (DEX) was used as a positive control.

**Figure 7:**
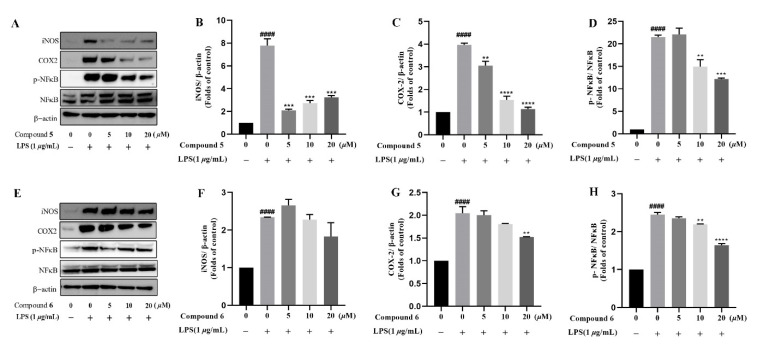
Compounds **5** and **6** suppressed pro-inflammatory expression in LPS-induced RAW 264.7 cells. Cells were incubated in different concentrations of compounds **5** and **6** for 2 h and then exposed to 1 μg/mL LPS for 12 h. (**A**–**H**), the protein level of iNOS, COX2, NF-*κ*B, and phosphor-NF-*κ*B were determined by Western blotting, β-actin was used as a control. Data represent mean ± SEM values of three experiments. ** *p* < 0.01, *** *p* < 0.001 and **** *p* < 0.0001 compared with LPS alone. ^####^ *p* < 0.0001 compared with DMSO alone.

Based on the traditional medicinal properties of *B. balsamifera*, we investigated the anti-inflammatory effects of compounds **1**–**6**. Following lipopolysaccharide (LPS) stimulation, we assessed the release of proinflammatory cytokines such as TNF-α and the generation of nitrite oxide pretreated with compounds to study their anti-inflammatory effects. According to the results of an ELISA assay, compounds **1**, **3**, **4**, **5** and **6** significantly suppressed LPS-induced TNF-α secretion, at the same time, compounds **5** and **6** de-creased the production of nitrite oxide induced by bacterial LPS in RAW 264.7 cells (Figure 5A,B). Therefore, we selected compounds **5** and **6** for the follow-up study. Following that, we looked at the drug toxicity of compounds in RAW 264.7 cells. The CCK-8 assay displays that no obvious cytotoxicity of compounds **5** and **6** at 20 μM in RAW 264.7 cells (Figure 5C). ELISA analysis shows that compounds **5** and **6** could dose-dependently inhibit LPS-induced TNF-α (Figure 6A,B), IL-6 (Figure 6C,D), and nitrite oxide generation (Figure 6E,F). As we know, nuclear factor-*κ*B (NF-*κ*B) plays an important role in the transcriptional regulation of inflammatory cytokines and the development of inflammation. To further study its anti-inflammatory mechanism, we measured the effect of compounds **5** and **6** on the activation of the transcription factor NF-*κ*B pathway. Western blot analysis confirms that compound **5** could dose-dependently down-regulate the expression of COX2 and p-NF-*κ*B, and also significantly down-regulate the expression of iNOS in RAW 264.7 cells induced by LPS (Figure 7A–D). Whereas, compound **6** could only dose-dependently reduce COX2 expression (Figure 7E–H), indicating its biological difference from **5**. Hence, compound **5** is considered to be a potent anti-inflammatory agent worthy for drug optimization.

According to the results of the anti-inflammatory activity, compounds **1**, **3**, **4**, **5** and **6** were found to suppress the secretion of inflammatory factor TNF-α, while compounds **5** and **6** also decreased the production of nitric oxide induced by bacterial LPS in RAW 264.7 cells, showing anti-inflammatory activity. Chemically, compounds **1**–**5** possess a similar chemical skeleton, while compounds **1**, **3**, **4**, and **5** all contain active hydrogen on oxygen atoms. Combined with the results of the anti-inflammatory activity, we speculated that active hydrogen on oxygen atoms may contribute to the reduction of TNF-α generation. Compound **5** also significantly inhibited the production of nitric oxide, which may be due to the presence of 9-OH and Δ^4,5^. In addition, compound **6**, a rearranged abietane-type diterpenoid, significantly reduced the production of nitric oxide, showing similar anti-inflammatory activity to the analogue jiadifenoic acid K, reported in the literature [20]. The results suggest that structural diversity leads to different anti-inflammatory activities.

## 3. Experimental Section

### 3.1. General Procedures

Optical rotations were determined on an Anton Paar MCP-100 digital polarimeter. UV and CD spectra were obtained on a Jasco J−815 circular dichroism spectrometer (JASCO, Tokyo, Japan). Semi-preparative HPLC was carried out by an Agilent 1260 liquid chromatograph (Agilent, Santa Clara, CA, USA) with a YMC-Pack ODS-A column (250 mm × 10 mm, i.d., 5 μm). NMR spectra were recorded on a Bruker AV-500 or AV-600 spectrometer (Billerica, MA, USA), with TMS as an internal standard. HRESIMS were collected by a SCIEX X500R QTOF MS spectrometer (Shimadzu Corporation, Tokyo, Japan). Silica gel (200–300 mesh; Qingdao Marine Chemical Inc., Qingdao, China), RP-18 silica gel (40–60 μm; Daiso Co., Tokyo, Japan), MCI gel CHP 20P (75–150 μm, Mitsubishi Chemical Industries, Tokyo, Japan), and Sephadex LH-20 (Amersham Pharmacia, Uppsala, Sweden) were used for column chromatography.

### 3.2. Plant Material

The dry aerial parts of *B. balsamifera* were purchased from Baoding Xiande Chinese Medicine Sales Co., Ltd., Guizhou province, China, in December 2019. The material was identified by Professor Bin Qiu at Yunnan University of Traditional Chinese Medicine, and a voucher specimen (CHYX0675) was deposited at the School of Pharmaceutical Sciences, Shenzhen University, China.

### 3.3. Extraction and Isolation

The dry aerial parts of *B. balsamifera* (50 kg) were soaked with 95% EtOH (300 L × 4 × 24 h) at room temperature. The 95% EtOH extracts were combined and evaporated under reduced pressure to afford a crude extract (2.4 kg), which was suspended in water and partitioned with EtOAc to gain an EtOAc soluble extract (1.6 kg). The EtOAc-soluble part was subjected to silica gel column chromatography, using a gradient of EtOAc in petroleum ether (20–100%) and MeOH in EtOAc (10–30%), to give eight fractions (Fr.1–Fr.8) based on thin-layer chromatography (TLC) analyses.

Fr.3 (200.0 g) was separated via MCI gel CHP 20P eluted with aqueous MeOH (50–100%) to provide fifteen fractions (Fr.3.1–Fr.3.15). Fr.3.3 (10.8 g) was further fractionated into nine parts (Fr.3.3.1–Fr.3.3.9) by a YMC GEL ODS-A-HG column eluted with gradient aqueous MeOH (50:50–100:0). Among them, Fr.3.3.6 (270.8 mg) was subjected to preparative TLC (petroleum ether–EtOAc (4:1)) to give Fr.3.3.6.1–Fr.3.3.6.6. Fr.3.3.6.4 (13.3 mg) was purified by semi-preparative HPLC on YMC-Pack ODS-A (aqueous MeCN, 65%, flow rate: 3 mL/min) to give compound **2** (*t*_R_ = 19.1 min, 1.4 mg). Fr.3.3.7 (1.5 g) was further separated via vacuum liquid chromatography (VLC) on silica gel washed with petroleum ether–EtOAc (30:1–1:1) to provide five portions (Fr.3.3.7.1–Fr.3.3.7.5). Of which, Fr.3.3.7.3 (160.8 mg) was further gel filtrated over Sephadex LH-20 (MeOH) followed by semi-preparative HPLC to give compounds **4** (aqueous MeOH, 70%, flow rate: 3 mL/min, *t*_R_ = 23.5 min, 5.3 mg) and **5** (aqueous MeCN, 62%, flow rate: 3 mL/min, *t*_R_ = 24.5 min, 54.0 mg). Similarly, Fr.3.3.8 (787.3 mg) was separated by VLC on silica gel eluted with petroleum ether–EtOAc (25:1–1:1) to provide eight portions (Fr.3.3.8.1–Fr.3.3.8.8). Fr.3.3.8.4 (167.0 mg) was further gel filtrated over Sephadex LH-20 (MeOH) followed by semi-preparative HPLC to give compound **3** (aqueous MeCN, 58%, flow rate: 3 mL/min, *t*_R_ = 20.2 min, 2.9 mg). Using the same protocols for the above fractions and subfractions, fraction Fr.3.4 yielded eleven subfractions Fr.3.4.1–Fr.3.4.11, compound **1** (aqueous MeCN, 75%, flow rate: 3 mL/min, *t*_R_ = 18.4 min, 14.7 mg) was isolated from Fr.3.4.5 and compound **6** (aqueous MeCN, 66%, flow rate: 3 mL/min, *t*_R_ = 18.49 min, 2.1 mg) was obtained from Fr.3.4.7.

### 3.4. Compound Characterization Data

Blusamiferoid A (**1**): colorless small quadrate crystals (MeOH); UV (MeOH) λ_max_ (logε) 248 (2.93) nm; {[α]${}_{D}^{20}$ +38.8 (c 0.07, MeOH); CD (MeOH) Δε_247_ −8.22, Δε_316_ +3.11}; HRMS (ESI) *m*/*z*: [M + Na]^+^ 339.1922 calculated for C_20_H_28_O_3_Na 339.1931; ^1^H and ^13^C NMR data, see Table 1.

Blusamiferoid B (**2**): white solids; UV (MeOH) λ_max_ (logε) 262 (2.65) nm; {[α]${}_{D}^{20}$ +16.67 (c 0.04, MeOH); CD (MeOH) Δε_263_ −4.75, Δε_340_ +1.57}; HRMS (ESI) *m*/*z*: [M + H]^+^ 315.1948 calculated for C_20_H_27_O_3_ 315.1955; ^1^H and ^13^C NMR data, see Table 1.

Blusamiferoid C (**3**): yellowish solids; UV (MeOH) λ_max_ (logε) 200 (2.90) nm; {[α]${}_{D}^{20}$ −120.0 (c 0.04, MeOH); CD (MeOH) Δε_217_ −4.89}; HRMS (ESI) *m*/*z*: [M + H]^+^ 317.2118 calculated for C_20_H_29_O_3_ 317.2111; ^1^H and ^13^C NMR data, see Table 1.

Blusamiferoid D (**4**): colorless small quadrate crystals (MeOH); UV (MeOH) λ_max_ (logε) 239 (2.87) nm; {[α]${}_{D}^{20}$ −29.17 (c 0.05, MeOH); CD (MeOH) Δε_253_ −11.17, Δε_356_ +2.02}; HRMS (ESI) *m*/*z*: [M + H]^+^ 343.1873 calculated for C_19_H_28_O_4_Na 343.1880; ^1^H and ^13^C NMR data, see Table 2.

Blusamiferoid E (**5**): yellowish gums; UV (MeOH) λ_max_ (logε) 250 (2.80) nm; {[α]${}_{D}^{20}$ −43.48 (c 0.07, MeOH); CD (MeOH) Δε_254_ −8.53, Δε_294_ +1.40, Δε_323_ +0.47, Δε_361_ +0.74}; HRMS (ESI) *m*/*z*: [M + H]^+^ 287.2005 calculated for C_19_H_27_O_2_ 287.2006; ^1^H and ^13^C NMR data, see Table 2.

Blusamiferoid F (**6**): yellowish gums; UV (MeOH) λ_max_ (logε) 220 (2.60), λ_max_ (logε) 279 (2.24) nm; {[α]${}_{D}^{20}$ −4.55 (c 0.04, MeOH); CD (MeOH) Δε_201_ −4.97, Δε_225_ +0.63}; HRMS (ESI) *m*/*z*: [M + H]^+^ 375.2165 calculated for C_22_H_31_O_5_ 375.2166; ^1^H and ^13^C NMR data, see Table 2.

### 3.5. Crystal Structure Determination of ***1*** and ***4***

Crystal data for **1** C_40_H_56_O_6_ (*M* = 632.84 g/mol): monoclinic, space group P2_1_ (no. 4), *a* = 6.35285(4) Å, *b* = 16.48619(7) Å, *c* = 16.53352(10) Å, *β* = 95.4460(5)°, *V* = 1723.810(16) Å^3^, *Z* = 2, *T* = 100.00(10) K, *μ*(Cu Kα) = 0.633 mm^−1^, *Dcalc* = 1.219 g/cm^3^, 32,528 reflections measured (5.37° ≤ 2Θ ≤ 148.784°), 6888 unique (*R*_int_ = 0.0241, *R*_sigma_ = 0.0184), which were used in all calculations. The final *R*_1_ was 0.0286 (I > 2σ(I)) and *wR*_2_ was 0.0744 (all data). The goodness of fit on *F*^2^ was 1.070. CCDC 2,144,854 for **1** contain the supplementary crystallographic data.

Crystal data for **4** C_38_H_56_O_8_ (*M* = 640.82 g/mol): monoclinic, space group P2_1_ (no. 4), *a* = 7.37143(4) Å, *b* = 12.23188(7) Å, *c* = 19.03670(10) Å, *β* = 93.1491(5)°, *V* = 1713.879(16) Å^3^, *Z* = 2, *T* = 99.99(10) K, *μ*(Cu Kα) = 0.687 mm^−1^, *Dcalc* = 1.242 g/cm^3^, 33,252 reflections measured (4.648° ≤ 2Θ ≤ 148.642°), 6840 unique (*R*_int_ = 0.0260, *R*_sigma_ = 0.0173), which were used in all calculations. The final *R*_1_ was 0.0278 (I > 2σ(I)) and *wR*_2_ was 0.0746 (all data). The goodness of fit on *F*^2^ was 1.054. CCDC 2,144,855 for **4** contain the supplementary crystallographic data.

### 3.6. ECD Calculations

Molecular Merck force field (MMFF) and DFT/TDDFT calculations were performed with a Spartan’14 software package (Wavefunction Inc., Irvine, CA, USA) and Gaussian 09 program package [21]. A CONFLEX conformational search generated low-energy conformers within a 10 kcal/mol energy and was finished by software CONFLEX 7. The predominant conformers were optimized by DFT calculation at B3LYP/6-31G(d,p) level with the PCM in MeOH. ECD calculations were further conducted at the B3LYP/6-31G(d,p) level with the PCM in MeOH. For comparisons of the calculated curves and experimental CD spectra, the program SpecDis 1.62 was used.

### 3.7. NMR Calculations of ***5***

A conformational search and geometric optimization were adopted using the same method as the ECD calculations in the Gaussian 09 software package [21]. Gauge-Independent Atomic Orbital (GIAO) calculations of NMR chemical shifts were submitted in Gaussian 09 by density functional theory (DFT) with the level of B3LYP/6-31G(d,p) in chloroform with the PCM solvent model. The calculated NMR chemical shifts were analyzed by subtracting the isotopic shifts for TMS calculated with the same methods [22]. Regression analysis of calculated versus experimental ^13^C NMR chemical shifts of **5** were carried out. Linear correlation coefficients (*R*^2^), mean absolute error (MAE), and corrected mean absolute error (CMAE) were calculated for the evaluation of the results. After Boltzmann weighing of the predicted chemical shift of each isomers, the DP4+ parameters were calculated using the excel file provided by Ariel M. Sarotti [23].

### 3.8. Anti-Inflammatory Activity

#### 3.8.1. Cell Culture

RAW 264.7, a mouse macrophage line (Procell Life Science & Technology Co., Wuhan, China), was cultured at 37 °C in a humid environment comprising 5% CO_2_ in high-glucose DMEM (C11995500BT, Gibco, Waltham, MA, USA) supplemented with 10% fetal bovine serum (FBS) (2094468CP, Gibco), 100 U/mL penicillin, and 100 μg/mL streptomycin. Compounds used in the cellular experiments were dissolved in DMSO (Sigma-Aldrich, Darmstadt, Germany).

#### 3.8.2. Cell Viability Assay

RAW 264.7 cells (2 × 10^4^ cells/mL) were planted into 96-well plates with DMEM that had been fully prepared. Cells were treated with various concentrations of compounds or DMSO for 24 h after an overnight culture. After that, each well was treated with a Cell Count Kit-8 (Beyotime, Shanghai, China) for 1 h at 37 °C. A microplate reader (BioTek, Winooski, VT, USA) was used to measure each well’s absorbance at 450 nm.

#### 3.8.3. ELISA of TNF-α and IL-6

RAW 264.7 cells were pretreated with compounds for 2 h and then stimulated with 1 μg/mL LPS for 12 h. The culture supernatants were collected and centrifuged from the treated cells. The concentrations of TNF-α and IL-6 were measured using the ELISA Kit (Proteintech, Chicago, IL, USA) according to the manufacturer’s instructions. Dexamethasone was used as a positive control.

#### 3.8.4. Determination of Nitrite Oxide

RAW 264.7 cells were treated with 1 μg/mL LPS with or without compounds for 24 h, the culture supernatants were collected and centrifuged. The production of nitrite oxide was measured using the Griess Kit (Beyotime, Shanghai, China) according to the manufacturer’s instructions. In short, 50 μL of the cell supernatants were mixed with 50 μL Griess reagent I and II, then the absorbance at 560 nm wavelength was measured using a microplate reader (BioTek, Winooski, VT, USA). Dexamethasone was used as a positive control.

#### 3.8.5. Western Blot

RAW 264.7 cells were incubated in different concentrations of compounds for 2 h and then exposed to 1 μg/mL LPS for 12 h. Total protein was extracted from cell lines after LPS treatment using a radioimmunoprecipitation assay (RIPA) buffer (Beyotime, Shanghai, China) including a protease and phosphatase inhibitor cocktail (Roche, Darmstadt, Germany), and protein samples were measured using the BCA assay (Thermo Scientific, Waltham, MA, USA). A 10% SDS-PAGE was used to separate equal quantities of protein extracts (15 μg), which were then transferred to PVDF membranes. The membranes were blocked with 5% BSA, then incubated overnight at 4 °C with the relevant antibodies, followed by a room temperature incubation with a horseradish peroxidase (HRP)-conjugated secondary antibody. The ECL kit (Bio-Rad, Hercules, CA, USA) and analysis system were used to view and quantify the bands (Bio-Rad, Hercules, CA, USA). ImageJ 1.51p software was used to perform densitometry analysis of the immunoblot findings (NIH, Bethesda, MD, USA).

#### 3.8.6. Statistical Analysis

All of the experiments in this study were carried out in triplicate. The data was provided as a mean ± standard error of the mean (SEM). Graphpad Prism 6 (GraphPad Software, San Diego, CA, USA) and Excel (Microsoft) were used to conduct statistical analyses, which included a Student’s *t*-test and a one-way ANOVA test. When * *p* ≤ 0.05, ** *p* ≤ 0.01, *** *p* ≤ 0.001, and **** *p* ≤ 0.0001, differences were judged as significant.

## 4. Conclusions

In summary, this study on *B. balsamifera* afforded six new diterpenoids belonging to pimarane-, rosane-, and abietane-types, which will add new aspects for the chemical profile of *B. balsamifera*. Compounds **1**, **3**, **4**, **5** and **6** could significantly inhibit LPS-induced TNF-α generation, showing their anti-inflammatory activity. The structure–activity relationship suggested that active hydrogen on oxygen atoms in compounds might be beneficial to inhibit the secretion of TNF-α, while the presence of 9-OH and *Δ*^4,5^ in compound **5** might contribute to reducing the production of nitrite oxides. In addition, compounds **5** and **6** could dose-dependently inhibit the production of TNF-α, IL-6 and nitrite oxides, and compound **5** significantly inhibits the phosphorylation of NF-κB in LPS-induced RAW 264.7 cells, suggesting that they play a potential role in inflammatory disorders. This finding indicates that the anti-inflammatory effect of *B. balsamifera* is not only related to volatile components, but also affected by other components in non-volatile parts, which is the result of the joint action of multiple components, and also provides the molecular template for the development of anti-inflammatory drugs.

## Figures and Tables

**Table 1:** ^1^H (600 MHz) and ^13^C (150 MHz) NMR data of **1**–**3** in CDCl_3_ (*δ* in ppm, *J* in Hz).

No.	1		2		3	
	*δ*_H_	*δ*_C_	*δ*_H_	*δ*_C_	*δ*_H_	*δ*_C_
1	Ha: 1.93, m Hb: 1.33, dt (13.3, 4.2)	39.6, t	1.62, overlap	29.5, t	Ha: 2.04, m Hb: 1.90, m	24.5, t
2	Ha: 1.87, m Hb: 1.53, overlap	18.9, t	Ha: 1.79, m Hb: 1.62, overlap	17.8, t	1.64, m	20.1, t
3	Ha: 2.33, d-like (13.3) Hb: 0.96, td (13.3, 3.3)	39.3, t	Ha: 2.21, m Hb: 1.52, m	27.8, t	Ha: 2.08, m Hb: 1.45, m	36.4, t
4		43.8, s		42.3, s		46.9, s
5	2.64, s	64.3, d	2.17, d (5.8)	49.0, d		125.4, s
6		205.9, s	4.74, d (5.8)	75.8, d	Ha: 2.41, dd (14.9, 5.5) Hb: 2.02, m	40.2, t
7	5.92, brs	125.6, d		192.1, s	4.46, ddd (10.6, 5.5, 1.7)	67.6, d
8		164.7, s		130.5, s		145.4, s
9	2.25, m	52.3, d		166.2, s		40.2, s
10		41.3, s		36.3, s		138.8, s
11	Ha: 1.83, m Hb: 1.53, overlap	20.9, t	Ha: 2.30, m Hb: 2.29, m	23.3, t	Ha: 1.58, m Hb: 1.39, m	31.5, t
12	Ha: 1.63, m Hb: 1.53, overlap	35.5, t	Ha: 1.60, overlap Hb: 1.46, ddd, (13.8, 13.4, 7.0)	33.1, t	1.55, m	32.0, t
13		37.8, s		34.6, s		37.8, s
14	2.22, brs	46.2, t	Ha: 2.40, d-like (17.4) Hb: 2.05, d-like (17.4)	33.7, t	5.32, brs	120.3, d
15	5.81, dd (17.5, 10.8)	148.3, d	5.74, dd, (17.5, 10.7)	145.9, d	5.70, dd, (17.3, 10.4)	146.8, d
16	Ha: 4.99, dd (17.5, 0.9) Hb: 4.96, dd (10.8, 0.9)	110.8, t	Ha: 4.95, dd (10.7, 1.1) Hb: 4.89, dd (17.5, 1.1)	111.6, t	Ha: 4.92, dd (10.4, 1.8) Hb: 4.80, dd (17.3, 1.8)	112.6, t
17	0.91, s	22.2, q	0.98, s	26.3, q	1.11, s	29.3, q
18		176.6, s		180.6, s		182.5, s
19	1.42, s	28.5, q	1.32, s	24.2, q	1.26, s	24.0, q
20	0.88, s	14.8, q	1.09, s	27.8, q	1.22, s	26.0, q
4-COOH	12.45, brs					

**Table 2:** ^1^H (600 MHz) and ^13^C (150 MHz) NMR data of **4**–**6** in CDCl_3_ (*δ* in ppm, *J* in Hz).

No.	4		5		6	
	*δ*_H_	*δ*_C_	*δ*_H_	*δ*_C_	*δ*_H_	*δ*_C_
1	Ha: 2.24, dt (13.5, 4.3) Hb: 1.33, overlap	25.5, t	Ha: 2.13, overlap Hb: 1.48, m	29.2, t	Ha: 1.92, dd (13.4, 5.6) Hb: 1.63, m	36.7, t
2	Ha: 1.70, m Hb: 1.51, m	18.4, t	Ha: 1.72, m Hb: 1.52, m	18.5, t	Ha: 1.84, m Hb: 1.76, m	19.4, t
3	Ha: 1.79, m Hb: 1.47, m	34.8, t	Ha: 2.14, m Hb: 2.09, m	33.9, t	1.80, overlap	31.6, t
4		74.4, s		149.4, s		52.0, s
5		80.2, s		133.0, s	1.97, t-like (5.5)	53.4, d
6		198.8, s		190.8, s	Ha: 1.80, overlap Hb: 1.72, m	26.2, t
7	5.73, brd (2.2) *	123.4, d	5.83, brd (2.1) *	128.2, d	2.62, m	29.4, t
8		163.1, s		159.4, s		127.7, s
9		74.0, s		73.6, s		151.6, s
10		42.9, s		44.4, s		86.2, s
11	Ha: 1.86, m Hb: 1.73, m	27.3, t	Ha: 1.93, dd (13.7, 4.3) Hb: 1.76, m	25.7, t	6.66, d (8.1)	115.4, d
12	Ha: 1.95, dt (13.0, 3.9) Hb: 1.33, overlap	31.5, t	Ha: 1.82, dt (13.1, 4.3) Hb: 1.41, m	31.8, t	6.93, dd (8.1, 2.3)	125.2, d
13		38.2, s		37.2, s		141.5, s
14	Ha: 2.80, dd (14.5,1.2) * Hb: 1.99, dd (14.5, 3.0) *	43.7, t	Ha: 2.60, dd (15.2, 1.4) * Hb: 2.06, dd (15.2, 3.0) *	42.9, t	6.90, d (2.3)	128.4, d
15	5.85, dd, (17.4, 10.7)	148.8, d	5.83, dd, (17.4, 10.7)	148.7, d	2.81, h (6.9)	33.4, d
16	Ha: 5.00, dd (17.4, 1.0) Hb: 4.95, dd (10.7, 1.0)	110.5, t	Ha: 5.00, dd (17.4, 1.0) Hb: 4.95, dd (10.7, 1.0)	110.6, t	1.21, d (6.9)	24.4, q
17	0.95, s	22.0, q	0.94, s	21.9, q	1.21, d (6.9)	24.4, q
18	1.67, s	26.2, q	1.97, s	22.7, q		177.1, s
19	1.10, s	20.1, q	1.23, s	25.2, q	Ha: 4.44, d (11.9) Hb: 4.29, d (11.9)	64.7, t
20					1.53, s	22.3, q
21						170.9, s
22					2.04, s	20.8, q
5-OH	4.82, brs					
9-OH	4.83, d (2.7)					

* H-7, Ha-14, and Hb-14 interact each other.

## Data Availability

Data is contained within the article or supplementary materials. CCDC 2144854 and 2144855 contain the supplementary crystallographic data for this paper.

## Supplementary Materials

The following supporting information can be downloaded at: https://www.mdpi.com/article/10.3390/molecules27092890/s1. Table S1: ^1^H and ^13^C NMR Data of **6** in pyridine-*d*_5_, Figures S1–S4: Optimized geometries of predominant conformers for **2**, **3**, **5** and **6**, Tables S2–S5: Energy analysis for conformers of **2**, **3**, **5** and **6**, Tables S6–S9: The Cartesian coordinates of the lowest energy conformers for **2**, **3**, **5** and **6**, Figure S5: Regression analysis of experimental versus calculated ^13^C NMR chemical shifts of **5**, Figure S6: DP4+ results of **5**, Figures S7–S16: NMR spectra of **1** in CDCl_3_, Figure S17: HRESIMS of **1**, Figures S18–S27: NMR spectra of **2** in CDCl_3_, Figure S28: HRESIMS of **2**, Figures S29–S37: NMR spectra of **3** in CDCl_3_, Figure S38: HRESIMS of **3**, Figures S39–S48: NMR spectra of **4** in CDCl_3_, Figure S49: HRESIMS of **4**, Figures S50–S60: NMR spectra of **5** in CDCl_3_, Figure S61: HRESIMS of **5**, Figures S62–S70: NMR spectra of **6** in CDCl_3_, Figure S71: HRESIMS of **3**, Figures S72–S77: NMR spectra of **6** in pyridine-d_5_, Figure S78: UV Spectra for compounds **1**–**6**, Tables S10 and S11: Crystal data and structure refinement for **1** and **4**.

## Sample Availability

Samples of the compounds **1**–**6** are available from the authors.
