# Supplementary material for: Diterpenoids from Blumea balsamifera and Their Anti-Inflammatory Activities
Source: Molecules. 2022 Apr 30;27(9):2890. doi: 10.3390/molecules27092890 (PMC9100843; doi:10.3390/molecules27092890)
Supplement: Supplementary file 1 [file molecules-27-02890-s001.zip › molecules-1706073-SI.pdf]

Supplementary Materials

Diterpenoids from *Blumea balsamifera* and Their Anti-Inflammatory Activities

Xiao-Ling Huang [1], Dai-Wei Wang [2], Ying-Qian Liu [1,*] and Yong-Xian Cheng [1,2,3,*]

[1] School of Pharmacy, Lanzhou University, Lanzhou 730000, China; huangxl19@lzu.edu.cn (X.-L. H.); yqliu@lzu.edu.cn (Y.-Q. L.)

[2] Institute for Inheritance-Based Innovation of Chinese Medicine, School of Pharmaceutical Sciences, Health Science Center, Shenzhen University, Shenzhen 518060, China; dweiwang@foxmail.com (D.-W. W.); yxcheng@szu.edu.cn (Y.-X. C.)

[3] Guangdong Key Laboratory of Functional Substances in Medicinal Edible Resources and Healthcare Products, School of Life Sciences and Food Engineering, Hanshan Normal University, Chaozhou 521041, China

* Correspondence: yqliu@lzu.edu.cn (Y.-Q. L.); yxcheng@szu.edu.cn (Y.-X. C.)

Contents

Table S1. ^{1}H (600 MHz) and ^{13}C NMR (150 MHz) Data of 6 in pyridine-d_5 (δ in ppm, J in Hz) 5

Figure S1. Optimized geometries of predominant conformers for (4*R*,5*S*,6*R*,10*R*,13*R*)-2 at the B3LYP/6-31G(d,p) level. 6

Figure S2. Optimized geometries of predominant conformers for (4*S*,7*R*,9*S*,13*R*)-3 at the B3LYP/6-31G(d,p) level....6

Figure S3. Optimized geometries of predominant conformers for (9*R*,10*S*,13*S*)-5 at the B3LYP/6-31G(d,p) level....6

Figure S4. Optimized geometries of predominant conformers for (4*R*,5*S*,10*S*)-6 at the B3LYP/6-31G(d,p) level....7

Table S2. Energy analysis for conformers of (4*R*,5*S*,6*R*,10*R*,13*R*)-2 for the calculations at the B3LYP/6-31G(d,p) level in MeOH....7

Table S3. Energy analysis for conformers of (4*S*,7*R*,9*S*,13*R*)-3 for the calculations at the B3LYP/6-31G(d,p) level in MeOH....7

Table S4. Energy analysis for conformers of (9*R*,10*S*,13*S*)-5 for the calculations at the B3LYP/6-31G(d,p) level in MeOH....8

Table S5. Energy analysis for conformers of (4*R*,5*S*,10*S*)-6 for the calculations at the B3LYP/6-31G(d,p) level in MeOH....8

Table S6. The Cartesian coordinates of the lowest energy conformers for (4*R*,5*S*,6*R*,10*R*,13*R*)-2....8

Table S7. The Cartesian coordinates of the lowest energy conformers for (4*S*,7*R*,9*S*,13*R*)-3 12

Table S8. The Cartesian coordinates of the lowest energy conformers for (9*R*,10*S*,13*S*)-5 ...15

Table S9. The Cartesian coordinates of the lowest energy conformers for (4*R*,5*S*,10*S*)-6 17

Figure S5. Regression analysis of experimental versus calculated ^{13}C NMR chemical shifts of (9*R*,10*S*,13*S*)-5 and (9*S*,10*S*,13*S*)-5 at the mPW1PW91/6-311+G(d,p) level. 23

Figure S6. DP4+ results of candidate (9*R*,10*S*,13*S*)-5 (Isomer 1) and (9*S*,10*S*,13*S*)-5 (Isomer 2). 23

Figure S7. ^{1}H NMR (600 MHz) spectrum of 1 in $CDCl_3$.....24

Figure S8. ^{13}C NMR and DEPT (150 MHz) spectra of 1 in $CDCl_3$. 24

Figure S9. ^{1}H-^{1}H COSY (600 MHz) spectrum of 1 in $CDCl_3$. 25

Figure S10. Enlarged ^{1}H-^{1}H COSY (600 MHz) spectrum of 1 in $CDCl_3$.....25

Figure S11. HSQC (600 MHz) spectrum of 1 in $CDCl_3$. 26

Figure S12. Enlarged HSQC (600 MHz) spectrum of 1 in $CDCl_3$.....26

Figure S14. Enlarged HMBC (600 MHz) spectrum of 1 in $CDCl_3$.....27

Figure S15. ROESY (600 MHz) spectrum of 1 in $CDCl_3$.....28

Figure S16. Enlarged ROESY (600 MHz) spectrum of 1 in $CDCl_3$. 28

Figure S17. HRESIMS of 1. 29

Figure S18. ^{1}H NMR (600 MHz) spectrum of 2 in $CDCl_3$.....29

Figure S19. ^{13}C NMR and DEPT (150 MHz) spectra of 2 in $CDCl_3$. 30

Figure S20. ^{1}H-^{1}H COSY (600 MHz) spectrum of 2 in $CDCl_3$. 30

Figure S21. Enlarged ^1H-^1H COSY (600 MHz) spectrum of 2 in $CDCl_3$. **31**
Figure S22. HSQC (600 MHz) spectrum of 2 in $CDCl_3$. **31**
Figure S23. Enlarged HSQC (600 MHz) spectrum of 2 in $CDCl_3$. **32**
Figure S24. HMBC (600 MHz) spectrum of 2 in $CDCl_3$. **32**
Figure S25. Enlarged HMBC (600 MHz) spectrum of 2 in $CDCl_3$. **33**
Figure S26. ROESY (600 MHz) spectrum of 2 in $CDCl_3$. **33**
Figure S27. Enlarged ROESY (600 MHz) spectrum of 2 in $CDCl_3$. **34**
Figure S28. HRESIMS of 2. .... **34**
Figure S29. ^1H NMR (600 MHz) spectrum of 3 in $CDCl_3$. **35**
Figure S30. ^{13}C NMR and DEPT (150 MHz) spectra of 3 in $CDCl_3$. **35**
Figure S31. ^1H-^1H COSY (600 MHz) spectrum of 3 in $CDCl_3$. **36**
Figure S32. HSQC (600 MHz) spectrum of 3 in $CDCl_3$. **36**
Figure S33. Enlarged HSQC (600 MHz) spectrum of 3 in $CDCl_3$. **37**
Figure S34. HMBC (600 MHz) spectrum of 3 in $CDCl_3$. **37**
Figure S35. Enlarged HMBC (600 MHz) spectrum of 3 in $CDCl_3$. **38**
Figure S36. ROESY (600 MHz) spectrum of 3 in $CDCl_3$. **38**
Figure S37. Enlarged ROESY (600 MHz) spectrum of 3 in $CDCl_3$. **39**
Figure S38. HRESIMS of 3. .... **39**
Figure S39. ^1H NMR (600 MHz) spectrum of 4 in $CDCl_3$. **40**
Figure S40. ^{13}C NMR and DEPT (150 MHz) spectra of 4 in $CDCl_3$. **40**
Figure S41. ^1H-^1H COSY (600 MHz) spectrum of 4 in $CDCl_3$. **41**
Figure S42. Enlarged ^1H-^1H COSY (600 MHz) spectrum of 4 in $CDCl_3$. **41**
Figure S43. HSQC (600 MHz) spectrum of 4 in $CDCl_3$. **42**
Figure S44. Enlarged HSQC (600 MHz) spectrum of 4 in $CDCl_3$. **42**
Figure S45. HMBC (600 MHz) spectrum of 4 in $CDCl_3$. **43**
Figure S46. Enlarged HMBC (600 MHz) spectrum of 4 in $CDCl_3$. **43**
Figure S47. ROESY (600 MHz) spectrum of 4 in $CDCl_3$. **44**
Figure S48. Enlarged ROESY (600 MHz) spectrum of 4 in $CDCl_3$. **44**
Figure S49. HRESIMS of 4. .... **45**
Figure S50. ^1H NMR (600 MHz) spectrum of 5 in $CDCl_3$. **45**
Figure S51. ^{13}C NMR and DEPT (150 MHz) spectra of 5 in $CDCl_3$. **46**
Figure S52. ^1H-^1H COSY (600 MHz) spectrum of 5 in $CDCl_3$. **46**
Figure S53. Enlarged ^1H-^1H COSY (600 MHz) spectrum of 5 in $CDCl_3$. **47**
Figure S54. HSQC (600 MHz) spectrum of 5 in $CDCl_3$. **47**
Figure S55. Enlarged HSQC (600 MHz) spectrum of 5 in $CDCl_3$. **48**
Figure S56. HMBC (600 MHz) spectrum of 5 in $CDCl_3$. **48**
Figure S57. Enlarged HMBC (600 MHz) spectrum of 5 in $CDCl_3$. **49**
Figure S58. ROESY (600 MHz) spectrum of 5 in $CDCl_3$. **49**

Figure S59. NOE irradiation spectrum (freq: 0.94 ppm) of compound 5 in $CDCl_3$. 50
Figure S60. NOE irradiation spectrum (freq: 1.23 ppm) of compound 5 in $CDCl_3$. 50
Figure S61. HRESIMS of 5. 51
Figure S62. ^{1}H NMR (600 MHz) spectrum of 6 in $CDCl_3$. 51
Figure S63. ^{13}C NMR and DEPT (150 MHz) spectra of 6 in $CDCl_3$. 52
Figure S64. ^{1}H-^{1}H COSY (600 MHz) spectrum of 6 in $CDCl_3$. 52
Figure S65. HSQC (600 MHz) spectrum of 6 in $CDCl_3$. 53
Figure S66. Enlarged HSQC (600 MHz) spectrum of 6 in $CDCl_3$. 53
Figure S67. HMBC (600 MHz) spectrum of 6 in $CDCl_3$. 54
Figure S68. Enlarged HMBC (600 MHz) spectrum of 6 in $CDCl_3$. 54
Figure S69. ROESY (600 MHz) spectrum of 6 in $CDCl_3$. 55
Figure S70. Enlarged ROESY (600 MHz) spectrum of 6 in $CDCl_3$. 55
Figure S71. HRESIMS of 6. 56
Figure S72. ^{1}H NMR (600 MHz) spectrum of 6 in pyridine-*d*$_5$. 56
Figure S73. ^{13}C NMR and DEPT (150 MHz) spectra of 6 in pyridine-*d*$_5$. 57
Figure S75. HSQC (600 MHz) spectrum of 6 in pyridine-*d*$_5$. 58
Figure S76. HMBC (600 MHz) spectrum of 6 in pyridine-*d*$_5$. 58
Figure S77. ROESY (600 MHz) spectrum of 6 in pyridine-*d*$_5$. 59
Figure S78. UV Spectra for compounds 1–6. 60
Table S10. Crystal data and structure refinement for 1 60
Table S11. Crystal data and structure refinement for 4 60

Table S1. ^{1}H (600 MHz) and ^{13}C NMR (150 MHz) Data of **6** in pyridine-d_5 (δ in ppm, J in Hz)

no	δ_H	δ_C
1	Ha: 1.76, overlap<br>Hb: 1.57, m	37.0, t
2	Ha: 1.69, m<br>Hb: 1.63, m	20.1, t
3	Ha: 1.84, m<br>Hb: 1.76, overlap	32.3, t
4		52.4, s
5	2.14, t-like (5.4)	53.6, d
6	2.04, m	27.2, t
7	3.00, m	30.4, t
8		125.7, s
9		155.0, s
10		86.6, s
11	7.12, d (8.1)	116.1, d
12	7.06, dd (8.1, 2.1)	129.1, d
13		140.2, s
14	7.22, d (2.1)	129.5, d
15	2.85, h (6.9)	34.0, d
16	1.25, d (6.9)	25.0, q
17	1.25, d (6.9)	25.0, q
18		177.7, s
19	Ha: 4.85, d (11.6)<br>Hb: 4.65, d (11.6)	65.3, t
20	1.56, s	22.5, q
21		171.0, s
22	2.07, s	21.0, q
4-COOH	11.3, brd	

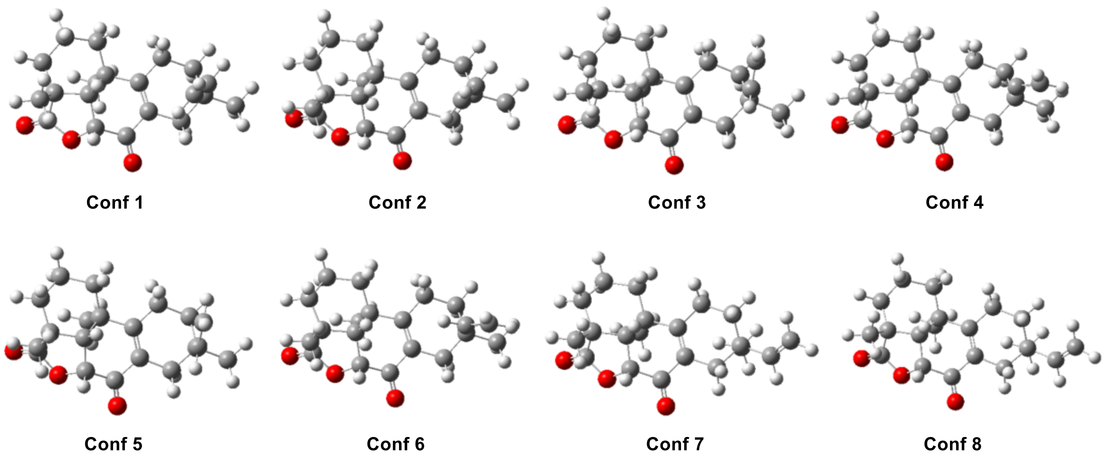

Figure S1. Optimized geometries of predominant conformers for (4*R*,5*S*,6*R*,10*R*,13*R*)-**2** at the B3LYP/6-31G(d,p) level.

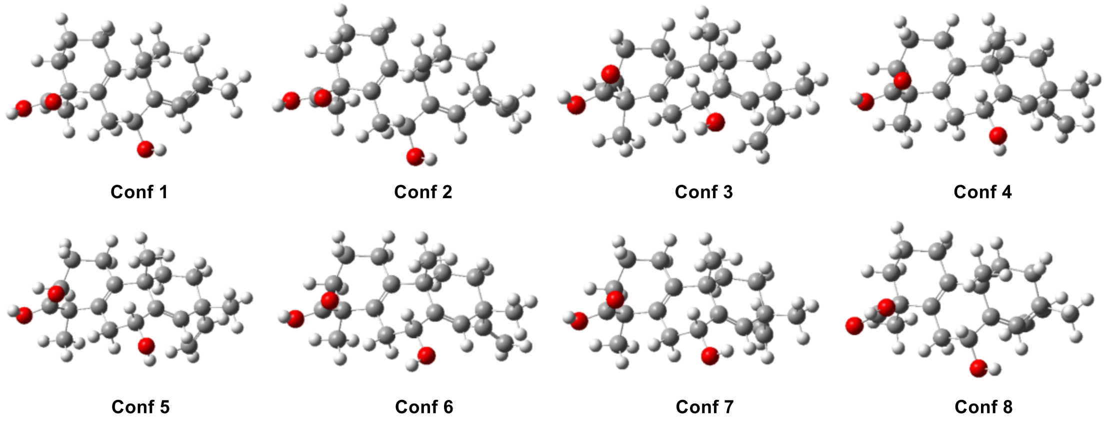

Figure S2. Optimized geometries of predominant conformers for (4*S*,7*R*,9*S*,13*R*)-**3** at the B3LYP/6-31G(d,p) level.

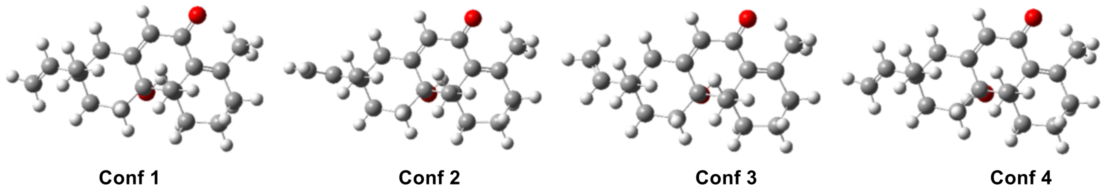

Figure S3. Optimized geometries of predominant conformers for (9*R*,10*S*,13*S*)-**5** at the B3LYP/6-31G(d,p) level.

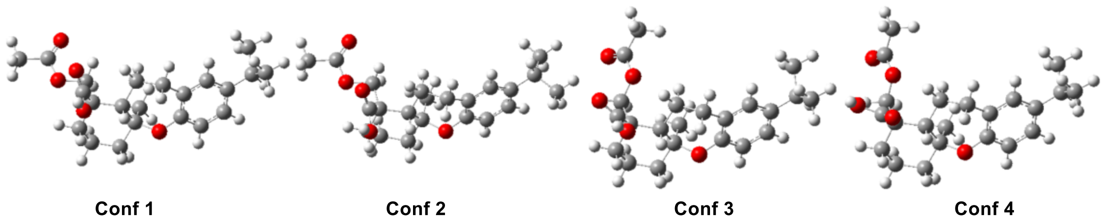

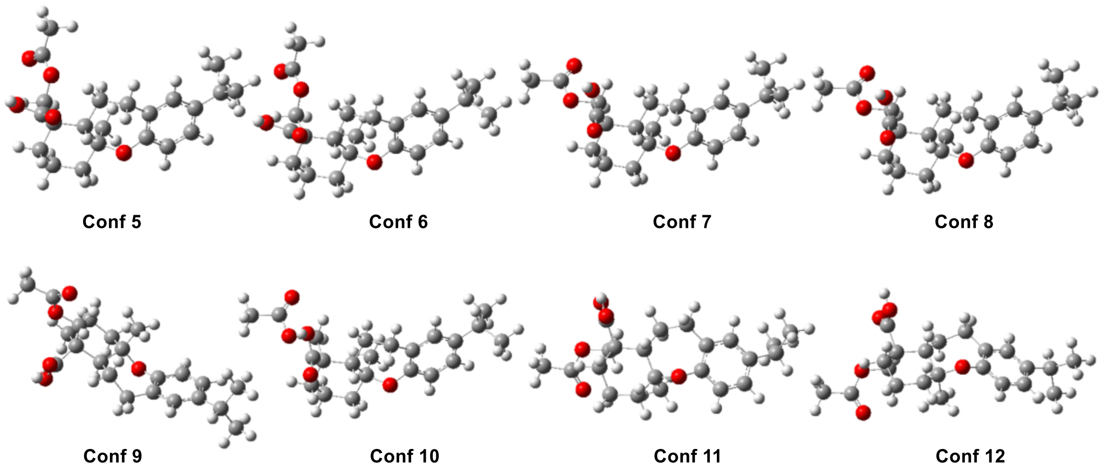

Figure S4. Optimized geometries of predominant conformers for (4*R*,5*S*,10*S*)-**6** at the B3LYP /6-31G(d,p) level.

Table S2. Energy analysis for conformers of (4*R*,5*S*,6*R*,10*R*,13*R*)-**2** for the calculations at the B3LYP/6-31G(d,p) level in MeOH

Confermors	G (Hartree)	*Δ*G (Kcal/mol)	Population
Conf 1	-1003.11287	0.00000	39.95%
Conf 2	-1003.11212	0.47000	18.07%
Conf 3	-1003.11149	0.86722	9.24%
Conf 4	-1003.11146	0.88416	8.98%
Conf 5	-1003.11087	1.25314	4.81%
Conf 6	-1003.11085	1.26757	4.70%
Conf 7	-1003.11122	1.03602	6.95%
Conf 8	-1003.11127	1.00590	7.31%

Table S3. Energy analysis for conformers of (4*S*,7*R*,9*S*,13*R*)-**3** for the calculations at the B3L YP/6-31G(d,p) level in MeOH

Confermors	G (Hartree)	*Δ*G (Kcal/mol)	Population
Conf 1	-1004.29231	0.20896	25.63%
Conf 2	-1004.29059	1.28828	4.14%
Conf 3	-1004.29149	0.72164	10.78%
Conf 4	-1004.29041	1.39809	3.44%
Conf 5	-1004.29183	0.51079	15.39%

Conf 6	-1004.28981	1.77523	1.82%
Conf 7	-1004.29004	1.62964	2.33%
Conf 8	-1004.29264	0.00000	36.47%

Table S4. Energy analysis for conformers of (9*R*,10*S*,13*S*)-**5** for the calculations at the B3LYP/6-31G(d,p) level in MeOH

Confermors	G (Hartree)	⊿G (Kcal/mol)	Population
Conf 1	-889.77107	0.26104	28.19%
Conf 2	-889.77149	0.00000	43.80%
Conf 3	-889.77116	0.20394	31.04%
Conf 4	-889.76821	2.05635	1.36%

Table S5. Energy analysis for conformers of (4*R*,5*S*,10*S*)-**6** for the calculations at the B3LYP/6-31G(d,p) level in MeOH

Confermors	G (Hartree)	⊿G (Kcal/mol)	Population
Conf 1	-1232.19570	-0.00941	14.60%
Conf 2	-1232.19569	-0.00063	14.39%
Conf 3	-1232.19469	0.62312	5.02%
Conf 4	-1232.19398	1.07179	2.35%
Conf 5	-1232.19428	0.88102	3.25%
Conf 6	-1232.19437	0.82580	3.56%
Conf 7	-1232.19570	-0.01004	14.62%
Conf 8	-1232.19568	0.00000	14.37%
Conf 9	-1232.19538	0.19327	10.37%
Conf 10	-1232.19538	0.19327	10.37%
Conf 11	-1232.19433	0.84714	3.44%
Conf 12	-1232.19439	0.81074	3.66%

Table S6. The Cartesian coordinates of the lowest energy conformers for (4*R*,5*S*,6*R*,10*R*,13*R*)-**2**

Conf 1	X axis(Å)	Y axis(Å)	Z axis(Å)	Conf 2	X axis(Å)	Y axis(Å)	Z axis(Å)
C	-2.3234	2.6926	0.3155	C	-2.5138	2.5553	-0.2083
C	-3.3295	1.5702	0.0767	C	-3.2735	1.5892	0.6967
C	-2.8304	0.2206	0.6166	C	-2.7419	0.15	0.8034

C	-1.3038	0.0954	0.599	C	-1.231	0.0257	0.6291
C	-0.5969	0.9227	-0.5118	C	-0.603	0.9124	-0.4943
C	-0.9056	2.4149	-0.2099	C	-0.9968	2.367	-0.1584
C	-1.1036	-1.3966	0.4532	C	-1.0689	-1.446	0.3153
C	0.2983	-1.7851	-0.0108	C	0.3249	-1.7825	-0.2275
C	1.3085	-0.6993	-0.2226	C	1.3299	-0.6724	-0.3344
C	0.8984	0.5672	-0.4564	C	0.8986	0.6034	-0.4578
C	2.7523	-1.1371	-0.2198	C	2.78	-1.0797	-0.336
C	3.7658	0.0317	-0.0955	C	3.7537	0.093	-0.0387
C	3.3197	1.1394	-1.075	C	3.3219	1.2915	-0.9137
C	1.9066	1.6547	-0.7737	C	1.8797	1.7398	-0.64
C	3.9165	0.6258	1.3062	C	3.824	0.5356	1.4241
C	3.3341	0.2244	2.4445	C	3.2157	-0.0101	2.4863
C	5.1578	-0.4827	-0.5134	C	5.1769	-0.3405	-0.4426
C	-3.2191	-0.955	-0.2615	C	-3.1959	-0.8338	-0.2591
O	-4.3058	-1.1195	-0.7907	O	-4.2848	-0.8492	-0.8069
O	-2.1728	-1.831	-0.3754	O	-2.1923	-1.7447	-0.5075
C	-3.4026	-0.0299	2.0223	C	-3.2333	-0.4284	2.1514
C	-1.0231	0.6056	-1.9703	C	-1.0436	0.6154	-1.9567
O	0.6191	-2.9607	-0.1828	O	0.6423	-2.9295	-0.5383
H	-0.8843	0.4173	1.5649	H	-0.7259	0.2638	1.5777
H	-1.2255	-1.887	1.4286	H	-1.1595	-2.0453	1.2308
H	-2.6981	3.6249	-0.1235	H	-2.8793	2.4583	-1.236
H	-2.2618	2.8764	1.3963	H	-2.7555	3.5842	0.0863
H	-3.5724	1.5166	-0.9892	H	-4.3298	1.5841	0.3997
H	-4.2793	1.8442	0.5544	H	-3.2506	2.0386	1.6998
H	-0.2096	2.8055	0.5436	H	-0.6294	2.641	0.8393
H	-0.7371	3.0226	-1.1086	H	-0.5413	3.0731	-0.863
H	2.9273	-1.8682	0.5797	H	2.9488	-1.8949	0.3789
H	2.9279	-1.6739	-1.1629	H	2.999	-1.4975	-1.3286
H	3.3375	0.7474	-2.1018	H	3.4038	1.0135	-1.9742
H	4.0205	1.9845	-1.0615	H	3.996	2.1468	-0.7735
H	1.9562	2.3491	0.0732	H	1.8604	2.3672	0.2593
H	1.5712	2.2328	-1.6426	H	1.5668	2.3704	-1.4803
H	4.593	1.4796	1.3751	H	4.4613	1.4012	1.6141
H	3.5413	0.7422	3.3772	H	3.3649	0.4097	3.4775
H	2.655	-0.6186	2.4976	H	2.5728	-0.88	2.419
H	5.1458	-0.8777	-1.5359	H	5.2215	-0.6238	-1.5005
H	5.9087	0.3159	-0.4818	H	5.9026	0.4675	-0.2908
H	5.4982	-1.2875	0.1488	H	5.5104	-1.2035	0.1458
H	-3.064	0.7371	2.7273	H	-2.751	0.0781	2.9948
H	-4.4988	-0.0147	2.007	H	-4.3174	-0.2973	2.2545
H	-3.11	-1.0065	2.4228	H	-3.0434	-1.502	2.2532
H	-0.85	-0.4421	-2.2392	H	-0.7288	-0.3733	-2.3078
H	-2.0719	0.8177	-2.176	H	-2.1248	0.6664	-2.1016
H	-0.4463	1.2088	-2.6832	H	-0.5975	1.3441	-2.6457
**Conf 3**	**X axis(Å)**	**Y axis(Å)**	**Z axis(Å)**	**Conf 4**	**X axis(Å)**	**Y axis(Å)**	**Z axis(Å)**
C	-2.2486	2.7462	0.0894	C	-2.3591	2.7192	0.2716
C	-3.2961	1.6451	-0.0489	C	-3.3803	1.6024	0.0745
C	-2.8473	0.3315	0.6107	C	-2.8944	0.265	0.6552
C	-1.3268	0.1465	0.6071	C	-1.3696	0.1205	0.6331
C	-0.5894	0.8455	-0.5701	C	-0.659	0.9024	-0.5081
C	-0.8409	2.369	-0.3997	C	-0.9484	2.4075	-0.2537
C	-1.1841	-1.3591	0.5932	C	-1.1883	-1.3777	0.5348
C	0.1993	-1.8407	0.1628	C	0.2056	-1.7976	0.0741
C	1.2518	-0.8177	-0.1388	C	1.2296	-0.731	-0.1748
C	0.8918	0.4388	-0.4808	C	0.8331	0.5316	-0.4493
C	2.6746	-1.3128	-0.1058	C	2.6659	-1.1942	-0.1696
C	3.7364	-0.1817	-0.0444	C	3.714	-0.0491	-0.1425
C	3.3477	0.8785	-1.0995	C	3.2531	1.0572	-1.1173
C	1.9399	1.4537	-0.892	C	1.85	1.5958	-0.813

C	3.8591	0.3384	1.3891	C	3.8253	0.5147	1.2783
C	3.9973	1.6077	1.796	C	4.9282	0.6399	2.0299
C	5.11	-0.7761	-0.4153	C	5.0572	-0.6155	-0.6531
C	-3.283	-0.9018	-0.1597	C	-3.3028	-0.9336	-0.1821
O	-4.3766	-1.0707	-0.6732	O	-4.3944	-1.1014	-0.6998
O	-2.2715	-1.8238	-0.1943	O	-2.2678	-1.8254	-0.2732
C	-3.4261	0.2296	2.0322	C	-3.4612	0.0672	2.0714
C	-1.0299	0.4191	-1.9962	C	-1.0978	0.5427	-1.9531
O	0.4719	-3.0385	0.0869	O	0.5092	-2.9817	-0.0681
H	-0.894	0.5355	1.5419	H	-0.9404	0.4686	1.5856
H	-1.321	-1.7572	1.6079	H	-1.3097	-1.8352	1.5261
H	-2.5873	3.6468	-0.4365	H	-2.7252	3.6419	-0.1943
H	-2.1824	3.0278	1.1487	H	-2.288	2.9362	1.3456
H	-3.5429	1.5052	-1.1059	H	-3.6299	1.5175	-0.9877
H	-4.2342	1.9963	0.4006	H	-4.3239	1.9034	0.5483
H	-0.1341	2.7951	0.3239	H	-0.2424	2.8145	0.4816
H	-0.6435	2.8904	-1.3454	H	-0.7793	2.983	-1.1732
H	2.8109	-1.9914	0.7471	H	2.8353	-1.8582	0.6891
H	2.8325	-1.9189	-1.0088	H	2.8117	-1.8067	-1.0702
H	3.3812	0.4169	-2.097	H	3.2511	0.6584	-2.1417
H	4.077	1.6977	-1.1303	H	3.9645	1.8935	-1.1183
H	1.9841	2.2408	-0.1323	H	1.9176	2.3175	0.0094
H	1.6383	1.9381	-1.8284	H	1.5097	2.1508	-1.6952
H	3.8702	-0.4287	2.1655	H	2.8925	0.8474	1.7341
H	4.0945	1.8374	2.8535	H	4.8664	1.0558	3.0318
H	4.0404	2.4452	1.1092	H	5.9134	0.3413	1.6887
H	5.1002	-1.201	-1.4257	H	4.9553	-1.0235	-1.6658
H	5.895	-0.0109	-0.3884	H	5.8309	0.1598	-0.6975
H	5.4006	-1.5771	0.275	H	5.42	-1.4268	-0.0113
H	-3.0577	1.0434	2.6662	H	-3.1091	0.8524	2.7492
H	-4.521	0.2845	2.0139	H	-4.5572	0.0954	2.0617
H	-3.1696	-0.7175	2.5189	H	-3.1781	-0.8994	2.5017
H	-0.8975	-0.654	-2.1724	H	-0.9378	-0.5151	-2.1885
H	-2.07	0.6523	-2.2217	H	-2.1454	0.7598	-2.1593
H	-0.4316	0.9353	-2.7579	H	-0.5188	1.1158	-2.6887
**Conf 5**	**X axis(Å)**	**Y axis(Å)**	**Z axis(Å)**	**Conf 6**	**X axis(Å)**	**Y axis(Å)**	**Z axis(Å)**
C	2.4434	-2.5471	-0.5119	C	2.5548	-2.5595	-0.2851
C	3.2328	-1.7328	0.5096	C	3.318	-1.6494	0.6732
C	2.7546	-0.2996	0.7971	C	2.8036	-0.2104	0.8463
C	1.2505	-0.0971	0.6377	C	1.2959	-0.0582	0.6653
C	0.5965	-0.8107	-0.5892	C	0.6669	-0.8802	-0.5061
C	0.9343	-2.3098	-0.4401	C	1.0399	-2.3546	-0.2391
C	1.1452	1.4075	0.5118	C	1.1552	1.4291	0.4235
C	-0.2302	1.862	0.0097	C	-0.2289	1.8092	-0.1151
C	-1.2764	0.8132	-0.2358	C	-1.2486	0.7183	-0.2821
C	-0.8933	-0.4526	-0.5154	C	-0.832	-0.5548	-0.4676
C	-2.7079	1.2741	-0.2009	C	-2.6913	1.1519	-0.2828
C	-3.7271	0.1171	-0.0116	C	-3.7056	-0.0118	-0.1094
C	-3.351	-1.0055	-1.0067	C	-3.2554	-1.1919	-1.0004
C	-1.9132	-1.5193	-0.8411	C	-1.8231	-1.6677	-0.7232
C	-3.7681	-0.302	1.4591	C	-3.7445	-0.4503	1.3588
C	-3.8199	-1.5431	1.9619	C	-4.81	-0.517	2.1696
C	-5.135	0.641	-0.3595	C	-5.083	0.4751	-0.6109
C	3.2519	0.7926	-0.1316	C	3.2797	0.8185	-0.1624
O	4.344	0.8357	-0.6715	O	4.3735	0.8464	-0.6997
O	2.2849	1.7651	-0.2642	O	2.2897	1.7532	-0.374
C	3.2587	0.0847	2.2081	C	3.2897	0.2945	2.2253
C	1.0567	-0.3482	-2.0019	C	1.1248	-0.5169	-1.9481
O	-0.5005	3.0497	-0.162	O	-0.527	2.9732	-0.3783
H	0.7312	-0.4336	1.548	H	0.7792	-0.3365	1.5965
H	1.2509	1.8834	1.4956	H	1.2446	1.9818	1.368

H	2.8185	-2.3343	-1.5185	H	2.931	-2.4163	-1.3036
H	2.6451	-3.6134	-0.3501	H	2.7811	-3.6048	-0.0401
H	4.2905	-1.7301	0.2174	H	4.3771	-1.6434	0.3863
H	3.1868	-2.3044	1.4476	H	3.28	-2.1474	1.6526
H	0.5518	-2.6939	0.5147	H	0.6597	-2.6728	0.7405
H	0.456	-2.9031	-1.2287	H	0.5826	-3.0193	-0.9816
H	-2.8404	2.0159	0.5983	H	-2.8542	1.8937	0.5109
H	-2.9105	1.7986	-1.1448	H	-2.8762	1.6679	-1.2349
H	-4.0505	-1.8483	-0.9416	H	-3.9413	-2.0425	-0.8931
H	-3.4533	-0.6182	-2.0309	H	-3.3132	-0.8874	-2.0552
H	-1.8858	-2.2796	-0.0528	H	-1.826	-2.3411	0.1424
H	-1.6345	-2.0225	-1.7747	H	-1.5021	-2.2597	-1.5882
H	-3.7915	0.519	2.1779	H	-2.7885	-0.7371	1.797
H	-3.8647	-1.6983	3.0364	H	-4.6976	-0.8444	3.1995
H	-3.842	-2.4307	1.3402	H	-5.813	-0.2552	1.8509
H	-5.1835	0.9937	-1.3962	H	-5.0331	0.7909	-1.6598
H	-5.8914	-0.1445	-0.2438	H	-5.8371	-0.3182	-0.5513
H	-5.4226	1.4793	0.2863	H	-5.4406	1.3335	-0.0305
H	2.7532	-0.5063	2.9799	H	2.7935	-0.2467	3.0386
H	4.3367	-0.0985	2.2953	H	4.3711	0.1445	2.3311
H	3.1075	1.1431	2.445	H	3.1123	1.3641	2.3784
H	0.7806	0.6878	-2.2262	H	0.825	0.4917	-2.2526
H	2.1362	-0.4206	-2.1505	H	2.2066	-0.5741	-2.0858
H	0.5885	-0.9672	-2.778	H	0.6766	-1.2052	-2.6762
**Conf 7**	**X axis(Å)**	**Y axis(Å)**	**Z axis(Å)**	**Conf 8**	**X axis(Å)**	**Y axis(Å)**	**Z axis(Å)**
C	2.6216	2.5516	0.2866	C	2.5741	2.6747	-0.2923
C	3.5033	1.5883	-0.5032	C	3.4921	1.5215	0.101
C	2.9874	0.1523	-0.6964	C	3.0393	0.1805	-0.4973
C	1.4666	0.0343	-0.7554	C	1.5249	0.0943	-0.7147
C	0.6779	0.9212	0.2586	C	0.6775	0.9662	0.2523
C	1.1297	2.3748	0.0013	C	1.0761	2.4381	-0.0397
C	1.2513	-1.4362	-0.4714	C	1.2621	-1.3872	-0.5735
C	-0.2064	-1.7706	-0.1318	C	-0.2009	-1.7362	-0.3148
C	-1.2194	-0.6579	-0.1641	C	-1.2094	-0.6253	-0.2578
C	-0.8024	0.6202	-0.0134	C	-0.8044	0.6432	-0.0203
C	-2.6658	-1.0683	-0.2756	C	-2.6559	-1.0448	-0.3717
C	-3.6776	0.0206	0.1725	C	-3.6716	-0.024	0.2041
C	-3.2152	1.3758	-0.4091	C	-3.2526	1.3827	-0.2718
C	-1.7907	1.7636	0.0057	C	-1.8213	1.7593	0.1278
C	-5.0687	-0.4027	-0.2984	C	-5.0701	-0.4333	-0.2573
C	-5.9168	0.2772	-1.0825	C	-5.9571	0.2954	-0.9488
C	-3.7331	0.096	1.7149	C	-3.6758	-0.085	1.748
C	3.2711	-0.8356	0.4196	C	3.2573	-0.9891	0.4457
O	4.2637	-0.8565	1.1268	O	4.2479	-1.1742	1.133
O	2.2383	-1.7425	0.5097	O	2.1791	-1.8314	0.4168
C	3.6756	-0.4245	-1.9557	C	3.8132	-0.1073	-1.795
C	0.8832	0.6166	1.7718	C	0.8664	0.6832	1.7669
O	-0.5589	-2.9199	0.1288	O	-0.5634	-2.9051	-0.1836
H	1.1138	0.2752	-1.7698	H	1.2701	0.4024	-1.7408
H	1.4727	-2.0359	-1.364	H	1.5164	-1.9024	-1.5098
H	2.8252	2.4417	1.3569	H	2.7148	2.8755	-1.3628
H	2.912	3.5814	0.0434	H	2.8901	3.5894	0.2236
H	4.5041	1.5763	-0.0534	H	3.5702	1.4728	1.1916
H	3.6305	2.0436	-1.4958	H	4.5124	1.7603	-0.2271
H	0.9268	2.6589	-1.0397	H	0.5537	2.8012	-0.9342
H	0.5711	3.0769	0.6326	H	0.7453	3.0882	0.7809
H	-2.847	-1.3373	-1.3258	H	-2.8606	-1.2157	-1.4381
H	-2.8465	-1.9778	0.3124	H	-2.8121	-2.0085	0.1306
H	-3.2473	1.3318	-1.5071	H	-3.9362	2.1481	0.1181
H	-3.8969	2.1829	-0.1109	H	-3.322	1.4339	-1.3679
H	-1.4597	2.5559	-0.6751	H	-1.5401	2.6183	-0.4895

H	-1.8104	2.1959	1.0134	H	-1.8157	2.1045	1.1686
H	-5.401	-1.3805	0.0563	H	-5.3717	-1.4458	0.0186
H	-6.884	-0.1462	-1.3396	H	-6.9246	-0.1241	-1.2113
H	-5.6911	1.2576	-1.4865	H	-5.7637	1.3126	-1.2702
H	-2.7542	0.2986	2.1608	H	-2.6864	0.0993	2.1784
H	-4.4161	0.888	2.0447	H	-4.3621	0.6599	2.1682
H	-4.0894	-0.8467	2.148	H	-3.9992	-1.0695	2.1075
H	3.332	0.0889	-2.8606	H	3.6115	0.6592	-2.5511
H	4.7636	-0.3013	-1.8909	H	4.8939	-0.1247	-1.611
H	3.4957	-1.4962	-2.0907	H	3.5546	-1.0804	-2.2261
H	0.5114	-0.3703	2.0678	H	0.623	-0.3513	2.0329
H	1.9289	0.6591	2.0836	H	1.8763	0.8739	2.1275
H	0.3398	1.3468	2.3852	H	0.2058	1.3228	2.3661

Table S7. The Cartesian coordinates of the lowest energy conformers for (4*S*,7*R*,9*S*,13*R*)-**3**

Conf 1	X axis(Å)	Y axis(Å)	Z axis(Å)	Conf 2	X axis(Å)	Y axis(Å)	Z axis(Å)
C	-2.6185	-2.3917	-0.0669	C	-2.6907	-2.3831	-0.1543
C	-3.0635	-1.4779	1.0636	C	-3.1284	-1.5395	1.0322
C	-2.7086	-0.0005	0.7776	C	-2.7782	-0.0469	0.8321
C	-1.2931	0.1506	0.1837	C	-1.3679	0.1423	0.237
C	-0.5521	-0.8974	-0.2638	C	-0.6282	-0.8749	-0.2786
C	-1.1075	-2.3122	-0.2484	C	-1.1813	-2.2896	-0.3425
C	-0.7628	1.5746	0.1063	C	-0.8416	1.5695	0.2391
C	0.3995	1.7498	-0.8696	C	0.3034	1.8044	-0.744
C	1.4319	0.6796	-0.6017	C	1.3471	0.7281	-0.5529
C	0.8946	-0.7454	-0.8149	C	0.8144	-0.6872	-0.8309
C	2.6809	0.9374	-0.157	C	2.606	0.9717	-0.1288
C	3.732	-0.1041	0.1756	C	3.6792	-0.074	0.1074
C	3.3124	-1.4891	-0.3659	C	3.2371	-1.4533	-0.4368
C	1.83	-1.7716	-0.1126	C	1.7563	-1.7395	-0.1787
C	3.9873	-0.2112	1.6788	C	3.9084	-0.1811	1.6134
C	3.3327	0.4034	2.6755	C	5.0567	-0.0286	2.2889
C	5.0556	0.3071	-0.4977	C	4.9434	0.3574	-0.6655
C	-3.7341	0.5462	-0.2368	C	-3.8129	0.557	-0.1397
O	-3.5618	0.7568	-1.428	O	-3.6509	0.8367	-1.318
O	-4.9579	0.7577	0.2945	O	-5.0322	0.7356	0.4136
C	-2.8297	0.7784	2.1041	C	-2.8896	0.6512	2.2037
C	0.8832	-1.0144	-2.3401	C	0.7983	-0.8826	-2.3669
O	0.9373	3.0618	-0.7207	O	0.8354	3.1103	-0.535
H	-3.1237	-2.1162	-1.0005	H	-2.9753	-3.4294	0.0062
H	-2.9066	-3.426	0.1535	H	-3.2038	-2.0535	-1.0659
H	-4.1434	-1.5987	1.2157	H	-4.2069	-1.6715	1.1852
H	-2.5806	-1.8087	1.9934	H	-2.6376	-1.9242	1.9368
H	-0.8604	-2.8335	-1.1788	H	-0.9408	-2.7548	-1.3038
H	-0.6269	-2.8663	0.5674	H	-0.6936	-2.8897	0.4356
H	-0.4555	1.8991	1.1097	H	-0.5186	1.8333	1.2552
H	-1.5588	2.2653	-0.1984	H	-1.6431	2.2756	-0.0107
H	0.0356	1.6735	-1.9008	H	-0.0774	1.7801	-1.7718
H	2.9771	1.968	0.0339	H	2.8997	1.9927	0.1128
H	3.9131	-2.2842	0.0954	H	3.8414	-2.2525	0.0132
H	3.5117	-1.5561	-1.4433	H	3.4236	-1.5169	-1.5169
H	1.6447	-1.7739	0.9703	H	1.5802	-1.7849	0.9043
H	1.6127	-2.7895	-0.4609	H	1.5338	-2.7433	-0.5634
H	4.8	-0.8807	1.9629	H	3.0185	-0.4042	2.2039
H	3.6159	0.2337	3.7098	H	5.073	-0.1284	3.3705
H	2.5009	1.0778	2.498	H	5.9996	0.1973	1.8038
H	4.9337	0.4207	-1.5818	H	4.7335	0.4733	-1.7362
H	5.4275	1.2628	-0.1075	H	5.3316	1.3194	-0.3092
H	5.8385	-0.443	-0.3321	H	5.744	-0.3857	-0.5703

H	-5.4858	1.0794	-0.4664	H	-5.5668	1.101	-0.3225
H	-3.7708	0.5486	2.6181	H	-3.8262	0.3894	2.7106
H	-2.0128	0.5259	2.7901	H	-2.0668	0.3596	2.8668
H	-2.8171	1.8626	1.9435	H	-2.8796	1.743	2.1081
H	0.0753	-0.47	-2.8434	H	-0.0136	-0.3173	-2.8399
H	1.823	-0.7091	-2.8151	H	1.7351	-0.551	-2.83
H	0.7431	-2.0775	-2.5651	H	0.662	-1.9342	-2.6423
H	1.6138	3.1534	-1.4127	H	1.4984	3.2444	-1.2329
**Conf 3**	**X axis(Å)**	**Y axis(Å)**	**Z axis(Å)**	**Conf 4**	**X axis(Å)**	**Y axis(Å)**	**Z axis(Å)**
C	-2.6214	-2.3922	-0.0694	C	-2.6854	-2.3928	-0.0528
C	-3.0636	-1.4808	1.0638	C	-3.1457	-1.4966	1.0849
C	-2.7149	-0.0022	0.7741	C	-2.8045	-0.0129	0.8159
C	-1.2964	0.1488	0.1854	C	-1.3858	0.1601	0.2319
C	-0.5547	-0.8993	-0.2604	C	-0.6319	-0.8743	-0.2223
C	-1.1104	-2.3139	-0.2502	C	-1.1745	-2.2951	-0.224
C	-0.76	1.5694	0.1144	C	-0.8714	1.587	0.173
C	0.4097	1.7509	-0.8548	C	0.3228	1.8014	-0.7612
C	1.4337	0.6737	-0.5926	C	1.3535	0.7165	-0.5417
C	0.8944	-0.7481	-0.8057	C	0.8104	-0.7032	-0.7761
C	2.6894	0.9302	-0.168	C	2.6311	0.9599	-0.1756
C	3.7423	-0.1095	0.1581	C	3.7009	-0.0869	0.0711
C	3.3121	-1.4984	-0.3665	C	3.2314	-1.4842	-0.3974
C	1.8297	-1.7733	-0.1019	C	1.7526	-1.7397	-0.0992
C	4.017	-0.2073	1.6591	C	3.9729	-0.1306	1.5739
C	3.3846	0.4249	2.659	C	5.1358	0.0711	2.2105
C	5.0582	0.2928	-0.5351	C	4.9462	0.2955	-0.7566
C	-3.7392	0.5496	-0.2394	C	-3.8315	0.5326	-0.1981
O	-3.5348	0.8901	-1.3948	O	-3.656	0.7551	-1.3863
O	-4.9941	0.6293	0.2544	O	-5.0588	0.729	0.3303
C	-2.847	0.7816	2.0963	C	-2.9428	0.7506	2.1499
C	0.8852	-1.0165	-2.3315	C	0.7864	-0.9404	-2.3068
O	0.9645	3.0549	-0.6859	O	0.8614	3.104	-0.5323
H	-2.9108	-3.4268	0.1483	H	-2.9638	-3.4325	0.1547
H	-3.1266	-2.1126	-1.0019	H	-3.1881	-2.1126	-0.9864
H	-2.5735	-1.8087	1.9909	H	-4.2251	-1.6301	1.23
H	-4.1413	-1.6081	1.2236	H	-2.6642	-1.8321	2.0137
H	-0.8635	-2.8324	-1.1822	H	-0.9168	-2.8057	-1.1575
H	-0.6306	-2.8709	0.5641	H	-0.6935	-2.8517	0.5899
H	-0.4561	1.8882	1.1208	H	-0.6026	1.9055	1.1893
H	-1.5517	2.2653	-0.1888	H	-1.6679	2.2694	-0.1487
H	0.065	1.6838	-1.8932	H	-0.0106	1.7818	-1.8048
H	2.9885	1.9664	-0.0104	H	2.9623	1.9875	-0.0339
H	3.9124	-2.2918	0.0985	H	3.8346	-2.2691	0.0785
H	3.5047	-1.5771	-1.4444	H	3.3966	-1.6023	-1.4763
H	1.6505	-1.7673	0.9819	H	1.5963	-1.7383	0.9878
H	1.6067	-2.7927	-0.4424	H	1.5129	-2.7556	-0.4386
H	4.8261	-0.8834	1.9371	H	3.1055	-0.3518	2.1971
H	3.6803	0.2622	3.6907	H	5.1858	0.0108	3.2939
H	2.5608	1.1099	2.4842	H	6.0597	0.2981	1.6901
H	4.9228	0.3984	-1.6184	H	4.7071	0.3613	-1.8254
H	5.4376	1.2504	-0.1571	H	5.3517	1.2703	-0.4591
H	5.8411	-0.4581	-0.3736	H	5.7436	-0.4491	-0.647
H	-5.5187	0.973	-0.4991	H	-5.5875	1.0484	-0.4314
H	-3.7934	0.555	2.6019	H	-3.8839	0.504	2.6561
H	-2.0366	0.5309	2.7907	H	-2.1269	0.5007	2.8381
H	-2.8321	1.8651	1.9318	H	-2.9426	1.8364	2.0006
H	0.0771	-0.4726	-2.8351	H	-0.0242	-0.3827	-2.7912
H	1.8247	-0.7094	-2.8059	H	1.7225	-0.6268	-2.7838
H	0.747	-2.0797	-2.5571	H	0.6425	-1.9982	-2.553
H	0.2902	3.685	-0.9893	H	1.1235	3.1401	0.4027
**Conf 5**	**X axis(Å)**	**Y axis(Å)**	**Z axis(Å)**	**Conf 6**	**X axis(Å)**	**Y axis(Å)**	**Z axis(Å)**

C	-2.6114	-2.3986	0.0229	C	-2.6915	-2.3836	-0.1597
C	-3.0768	-1.4438	1.1098	C	-3.1274	-1.5431	1.0293
C	-2.7337	0.0238	0.7651	C	-2.7839	-0.049	0.8264
C	-1.3118	0.1657	0.1807	C	-1.3701	0.1405	0.2375
C	-0.5567	-0.8917	-0.2146	C	-0.629	-0.8765	-0.2761
C	-1.0997	-2.3105	-0.1461	C	-1.182	-2.2908	-0.346
C	-0.7955	1.5879	0.0508	C	-0.8381	1.5643	0.2463
C	0.413	1.7535	-0.8757	C	0.3147	1.8056	-0.7293
C	1.4346	0.6764	-0.5874	C	1.3501	0.7231	-0.543
C	0.8879	-0.7499	-0.7695	C	0.8166	-0.6894	-0.8214
C	2.7034	0.9297	-0.1978	C	2.6143	0.9671	-0.1369
C	3.7552	-0.1132	0.1293	C	3.6886	-0.0762	0.0951
C	3.306	-1.5106	-0.3513	C	3.2392	-1.4597	-0.4339
C	1.8267	-1.7653	-0.0558	C	1.7582	-1.7405	-0.1671
C	4.0621	-0.1749	1.6256	C	3.9328	-0.1748	1.5999
C	3.4378	0.4605	2.6287	C	5.0882	-0.0206	2.2628
C	5.0589	0.262	-0.6022	C	4.9455	0.3478	-0.6934
C	-3.7549	0.5156	-0.2816	C	-3.8169	0.5602	-0.1445
O	-3.5726	0.678	-1.4785	O	-3.6218	0.9688	-1.2793
O	-4.9858	0.7371	0.2288	O	-5.068	0.6079	0.3629
C	-2.8793	0.8562	2.0564	C	-2.9073	0.6537	2.1943
C	0.8638	-1.0464	-2.2903	C	0.8042	-0.884	-2.3579
O	0.9544	3.0636	-0.7022	O	0.864	3.1026	-0.5013
H	-3.1095	-2.1675	-0.9265	H	-2.9772	-3.4301	-0.0026
H	-2.8913	-3.4259	0.2831	H	-3.2039	-2.0497	-1.0702
H	-4.1569	-1.5694	1.2565	H	-4.2038	-1.6824	1.1888
H	-2.5999	-1.7302	2.0572	H	-2.63	-1.9249	1.9316
H	-0.8378	-2.8677	-1.0514	H	-0.9408	-2.7528	-1.3087
H	-0.6225	-2.8249	0.6972	H	-0.6955	-2.8938	0.4306
H	-0.541	1.9609	1.0521	H	-0.5192	1.8221	1.2653
H	-1.5874	2.2513	-0.3185	H	-1.6356	2.2753	-0.0019
H	0.0949	1.6856	-1.9219	H	-0.0465	1.7907	-1.7643
H	3.0373	1.9607	-0.0943	H	2.9092	1.9957	0.072
H	3.9072	-2.2967	0.1249	H	3.8433	-2.2566	0.0204
H	3.4806	-1.6198	-1.4295	H	3.4208	-1.5341	-1.5141
H	1.665	-1.7312	1.0302	H	1.5865	-1.7786	0.917
H	1.5915	-2.791	-0.3678	H	1.5317	-2.7459	-0.5451
H	4.8899	-0.8306	1.8988	H	3.048	-0.3919	2.1997
H	3.7607	0.319	3.6558	H	5.1156	-0.113	3.3446
H	2.5934	1.1209	2.4647	H	6.0258	0.2015	1.7657
H	4.8998	0.3449	-1.6843	H	4.725	0.4568	-1.7627
H	5.4552	1.2241	-0.2542	H	5.3388	1.3115	-0.3475
H	5.8388	-0.4923	-0.4418	H	5.7458	-0.396	-0.6012
H	-5.5101	1.0162	-0.5515	H	-5.5991	0.9951	-0.3646
H	-3.8231	0.6361	2.5698	H	-3.8495	0.3952	2.6924
H	-2.0671	0.6431	2.7612	H	-2.0913	0.3635	2.8663
H	-2.8785	1.9327	1.8502	H	-2.8954	1.7452	2.0952
H	0.0541	-0.5073	-2.7968	H	-0.0075	-0.3193	-2.8321
H	1.8003	-0.753	-2.7789	H	1.7413	-0.5505	-2.8193
H	0.7176	-2.1127	-2.495	H	0.6703	-1.9356	-2.6342
H	1.198	3.1461	0.2347	H	0.1787	3.7433	-0.7533
**Conf 7**	**X axis(Å)**	**Y axis(Å)**	**Z axis(Å)**	**Conf 8**	**X axis(Å)**	**Y axis(Å)**	**Z axis(Å)**
C	-2.5729	-2.3537	-0.556	C	-2.6285	-2.3961	-0.0367
C	-3.038	-1.7177	0.744	C	-3.0777	-1.4766	1.0871
C	-2.7321	-0.2025	0.7805	C	-2.7107	-0.0007	0.806
C	-1.3266	0.1176	0.2321	C	-1.3021	0.1478	0.1975
C	-0.5563	-0.7859	-0.4299	C	-0.5625	-0.9032	-0.2454
C	-1.0666	-2.1884	-0.7174	C	-1.1169	-2.3181	-0.2137
C	-0.8416	1.5417	0.461	C	-0.7754	1.5715	0.1039
C	0.3062	1.9579	-0.4573	C	0.3793	1.7379	-0.8828
C	1.3748	0.8898	-0.4237	C	1.4163	0.6739	-0.6089

C	0.8808	-0.4724	-0.9365	C	0.8813	-0.7547	-0.8041
C	2.6195	1.0895	0.0614	C	2.6664	0.9394	-0.1719
C	3.7145	0.0415	0.1616	C	3.721	-0.0964	0.1681
C	3.3248	-1.2122	-0.658	C	3.3022	-1.4883	-0.3564
C	1.8541	-1.5917	-0.4686	C	1.8214	-1.7711	-0.094
C	3.9879	-0.2164	1.6426	C	3.9823	-0.1864	1.6713
C	3.9821	-1.3796	2.3094	C	3.3294	0.4367	2.6639
C	5.0092	0.6202	-0.4456	C	5.0411	0.3102	-0.5149
C	-3.7817	0.5149	-0.0932	C	-3.7965	0.5694	-0.1292
O	-3.6246	0.9812	-1.2116	O	-4.9012	0.9678	0.2119
O	-5.0079	0.5676	0.4712	O	-3.4778	0.5676	-1.4399
C	-2.8682	0.2706	2.2429	C	-2.8036	0.761	2.1461
C	0.8659	-0.4104	-2.4841	C	0.8645	-1.0406	-2.3263
O	0.8034	3.2265	-0.0374	O	0.9125	3.0538	-0.7578
H	-2.8266	-3.4201	-0.5618	H	-2.9173	-3.4291	0.189
H	-3.0936	-1.9016	-1.4089	H	-3.1311	-2.1276	-0.9735
H	-4.1124	-1.9032	0.8674	H	-4.1604	-1.5876	1.2291
H	-2.538	-2.2237	1.5812	H	-2.6064	-1.812	2.0212
H	-0.8102	-2.4901	-1.7381	H	-0.8669	-2.8506	-1.137
H	-0.5625	-2.8883	-0.0395	H	-0.6378	-2.8617	0.61
H	-0.536	1.654	1.51	H	-0.4606	1.9062	1.1016
H	-1.6611	2.2556	0.3122	H	-1.5756	2.2571	-0.2015
H	-0.0645	2.0885	-1.4807	H	0.008	1.6463	-1.9101
H	2.8787	2.0687	0.4621	H	2.9609	1.9724	0.0085
H	3.9639	-2.0659	-0.4014	H	3.9065	-2.2769	0.1114
H	3.5052	-1.0359	-1.7268	H	3.4975	-1.5668	-1.4337
H	1.6714	-1.8295	0.5874	H	1.6403	-1.7617	0.9895
H	1.6678	-2.5189	-1.0257	H	1.6048	-2.7933	-0.4301
H	4.224	0.6749	2.2268	H	4.7982	-0.85	1.9596
H	4.1981	-1.4005	3.3738	H	3.617	0.2789	3.6989
H	3.7632	-2.3306	1.8384	H	2.4944	1.1062	2.4827
H	4.8661	0.9058	-1.4949	H	4.9147	0.4115	-1.5997
H	5.3477	1.5161	0.09	H	5.4124	1.2708	-0.1368
H	5.8244	-0.1126	-0.4096	H	5.8262	-0.4364	-0.3441
H	-5.5509	1.0275	-0.2031	H	-4.2748	0.9361	-1.874
H	-3.7979	-0.094	2.696	H	-3.7527	0.5529	2.655
H	-2.0389	-0.0963	2.8588	H	-1.9936	0.4728	2.8262
H	-2.8907	1.3638	2.3179	H	-2.7582	1.8469	2.0037
H	0.0347	0.1987	-2.859	H	0.053	-0.5041	-2.8322
H	1.7897	0.0237	-2.8842	H	1.8014	-0.7378	-2.8085
H	0.7618	-1.4056	-2.9304	H	0.7266	-2.1065	-2.5389
H	1.4604	3.4894	-0.7036	H	1.6023	3.1303	-1.4383

Table S8. The Cartesian coordinates of the lowest energy conformers for (9*R*,10*S*,13*S*)-**5**

Conf 1	X axis(Å)	Y axis(Å)	Z axis(Å)	Conf 2	X axis(Å)	Y axis(Å)	Z axis(Å)
C	0.018	1.9296	0.0365	C	-0.0556	1.8815	0.0448
C	-0.7983	0.8865	-0.19	C	-0.815	0.8095	-0.2376
C	-2.2478	1.1236	-0.5631	C	-2.2593	0.992	-0.659
C	-3.2571	0.1979	0.1577	C	-3.2472	6.00E-04	0.0016
C	-0.3116	-0.5515	-0.1906	C	-0.2648	-0.6058	-0.2569
C	-4.6461	0.4677	-0.4224	C	-4.5854	0.0974	-0.7327
C	-3.3289	0.5551	1.6586	C	-3.3748	0.3253	1.5074
C	1.4802	1.7837	0.2134	C	1.4047	1.7957	0.2698
C	1.1244	-0.729	0.4734	C	1.1557	-0.737	0.4502
C	2.0455	0.4082	0.0191	C	2.0381	0.4527	0.0576
C	3.2991	0.23	-0.4589	C	3.3133	0.3444	-0.3827
O	-0.2528	-0.9111	-1.5812	O	-0.1462	-0.9244	-1.654
O	2.1555	2.7583	0.5197	O	2.0247	2.7897	0.6264
C	3.943	-1.1374	-0.5138	C	4.0205	-0.9907	-0.4524

C	1.7204	-2.118	0.0888	C	1.8268	-2.0866	0.0493
C	3.2242	-2.1921	0.3202	C	3.3241	-2.0994	0.329
C	1.0632	-0.644	2.0255	C	1.0402	-0.6981	2.001
C	-1.3523	-1.5016	0.4527	C	-1.2814	-1.6195	0.3271
C	-2.7883	-1.2651	-0.031	C	-2.7095	-1.4361	-0.1992
C	4.1886	1.3287	-0.9859	C	4.1683	1.4964	-0.8497
C	-5.4855	-0.4077	-0.9927	C	-5.7896	0.3941	-0.2245
H	-0.3355	2.9557	0.0331	H	-0.4554	2.8904	0.0545
H	-2.5192	2.1736	-0.3849	H	-2.5912	2.0215	-0.4652
H	-2.3333	0.9742	-1.6493	H	-2.2911	0.8706	-1.7516
H	-4.9864	1.5024	-0.3551	H	-4.5425	-0.108	-1.8041
H	-3.6947	1.5786	1.8075	H	-3.7918	1.3274	1.6635
H	-4.0111	-0.1187	2.1906	H	-4.026	-0.3952	2.0157
H	-2.3544	0.4947	2.1527	H	-2.4095	0.3074	2.023
H	0.377	-0.3095	-2.0154	H	0.4828	-0.2944	-2.0473
H	3.978	-1.4621	-1.5615	H	4.104	-1.284	-1.5065
H	4.9803	-1.0731	-0.1629	H	5.042	-0.8897	-0.0654
H	1.2299	-2.9163	0.6588	H	1.3553	-2.9217	0.5813
H	1.5395	-2.3452	-0.9688	H	1.6907	-2.2921	-1.0194
H	3.5946	-3.1874	0.0484	H	3.7484	-3.0691	0.0435
H	3.455	-2.0535	1.3828	H	3.514	-1.9803	1.402
H	0.6382	-1.5534	2.4641	H	0.6431	-1.6375	2.4009
H	2.0605	-0.5221	2.4651	H	2.0161	-0.5441	2.4771
H	0.4567	0.2004	2.3708	H	0.385	0.1084	2.3477
H	-1.0972	-2.5423	0.2162	H	-0.9705	-2.6409	0.0744
H	-1.3496	-1.4186	1.5432	H	-1.3187	-1.5644	1.4188
H	-2.8452	-1.5451	-1.0915	H	-2.7244	-1.7008	-1.2657
H	-3.4536	-1.9513	0.5091	H	-3.3671	-2.159	0.3018
H	4.752	0.9733	-1.8562	H	4.7746	1.1915	-1.7103
H	3.632	2.2045	-1.3292	H	3.5836	2.356	-1.1872
H	4.9052	1.6371	-0.2184	H	4.8452	1.8141	-0.0508
H	-6.452	-0.0797	-1.365	H	-6.6641	0.4243	-0.8685
H	-5.2506	-1.4601	-1.1083	H	-5.9509	0.61	0.8258
**Conf 3**	**X axis(Å)**	**Y axis(Å)**	**Z axis(Å)**	**Conf 4**	**X axis(Å)**	**Y axis(Å)**	**Z axis(Å)**
C	-0.113	1.8477	0.2069	C	0.0214	1.9327	0.0357
C	-0.8636	0.7777	-0.1046	C	-0.8043	0.8966	-0.1913
C	-2.3283	0.9517	-0.4552	C	-2.2643	1.1569	-0.5011
C	-3.2706	-0.09	0.1956	C	-3.2556	0.1852	0.1828
C	-0.2885	-0.6217	-0.2279	C	-0.3086	-0.5396	-0.2371
C	-4.7066	-0.033	-0.325	C	-4.6543	0.483	-0.3576
C	-3.3438	0.1524	1.7189	C	-3.2998	0.4526	1.7028
C	1.3569	1.7799	0.3667	C	1.4869	1.7856	0.189
C	1.1615	-0.7635	0.4138	C	1.1153	-0.7241	0.4546
C	2.0055	0.464	0.0544	C	2.0522	0.4082	0.0185
C	3.2636	0.4064	-0.4413	C	3.3185	0.2236	-0.4238
O	-0.2201	-0.8598	-1.6439	O	-0.1737	-0.8546	-1.6341
O	1.9726	2.7657	0.7524	O	2.1608	2.7663	0.483
C	3.9914	-0.9082	-0.6142	C	3.9563	-1.1467	-0.4616
C	1.84	-2.0747	-0.0892	C	1.7136	-2.1158	0.0811
C	3.3474	-2.0727	0.13	C	3.2103	-2.1991	0.3507
C	1.1089	-0.8136	1.9677	C	1.0247	-0.6415	2.0061
C	-1.2628	-1.6851	0.3373	C	-1.3484	-1.5291	0.3474
C	-2.7158	-1.5	-0.1154	C	-2.7891	-1.2626	-0.1026
C	4.0778	1.5996	-0.8763	C	4.2233	1.3158	-0.937
C	-5.2364	0.8407	-1.1922	C	-5.5032	-0.36	-0.9618
H	-0.5306	2.8459	0.2909	H	-0.3283	2.9597	0.0609
H	-2.6638	1.9648	-0.1945	H	-2.3852	1.0866	-1.5917
H	-2.4012	0.8775	-1.5495	H	-2.5286	2.1917	-0.241
H	-5.3739	-0.8016	0.0691	H	-4.9961	1.5114	-0.2242
H	-3.8087	1.121	1.9403	H	-3.6693	1.4625	1.9193
H	-3.9407	-0.6211	2.217	H	-3.9666	-0.2566	2.2075

H	-2.3568	0.1568	2.1919	H	-2.3148	0.371	2.1725
H	0.3692	-0.1873	-2.0281	H	-0.9731	-0.5671	-2.1034
H	4.0374	-1.1405	-1.6857	H	4.0188	-1.4711	-1.5081
H	5.0257	-0.8082	-0.2627	H	4.9839	-1.0875	-0.0823
H	1.4055	-2.9476	0.4129	H	1.2049	-2.9128	0.637
H	1.6645	-2.2229	-1.1616	H	1.5574	-2.3396	-0.9813
H	3.7771	-3.0162	-0.2265	H	3.5819	-3.1959	0.0859
H	3.5785	-2.01	1.1997	H	3.4148	-2.0648	1.4193
H	0.7454	-1.7817	2.3292	H	0.5952	-1.5525	2.4364
H	2.1005	-0.6667	2.4125	H	2.0134	-0.5169	2.464
H	0.454	-0.0412	2.3854	H	0.4098	0.2002	2.3424
H	-0.9461	-2.6847	0.0142	H	-1.0961	-2.552	0.0408
H	-1.2552	-1.6912	1.4311	H	-1.3375	-1.5197	1.4407
H	-2.7796	-1.6959	-1.1947	H	-2.8625	-1.4785	-1.1768
H	-3.3317	-2.2689	0.3702	H	-3.4472	-1.9808	0.4039
H	4.6547	1.3552	-1.7756	H	4.8189	0.9482	-1.7805
H	3.4645	2.4643	-1.1421	H	3.6767	2.1832	-1.3157
H	4.7804	1.8867	-0.0878	H	4.9111	1.6393	-0.1498
H	-6.2824	0.7642	-1.4766	H	-6.4773	-0.012	-1.2953
H	-4.6677	1.6467	-1.6421	H	-5.2742	-1.4049	-1.1391

Table S9. The Cartesian coordinates of the lowest energy conformers for (4*R*,5*S*,10*S*)-**6**

**Conf 1**	**X axis(Å)**	**Y axis(Å)**	**Z axis(Å)**	**Conf 2**	**X axis(Å)**	**Y axis(Å)**	**Z axis(Å)**
C	-2.36	-1.9428	-1.7634	C	-2.2823	-1.4257	-2.2228
C	-2.9967	-1.7008	-0.4044	C	-2.9836	-1.5421	-0.8791
C	-2.2967	-0.5825	0.4106	C	-2.342	-0.6632	0.2259
C	-0.7391	-0.8314	0.4726	C	-0.7843	-0.9088	0.2973
C	-0.055	-1.2022	-0.8878	C	-0.0332	-0.9164	-1.0782
C	-0.8887	-2.2967	-1.6006	C	-0.8133	-1.7992	-2.0847
C	0.0033	0.2946	1.2339	C	-0.098	-0.0086	1.3543
C	1.39	-0.1147	1.729	C	1.2728	-0.5209	1.7952
C	2.4306	-0.2185	0.651	C	2.3624	-0.3397	0.7775
C	2.2993	-1.1017	-0.4198	C	2.2955	-0.9219	-0.4875
C	3.6125	0.5377	0.7536	C	3.5242	0.3734	1.1253
C	4.6534	0.4101	-0.1821	C	4.6079	0.4954	0.2391
C	4.5041	-0.5115	-1.2249	C	4.5225	-0.1311	-1.0098
C	3.3389	-1.2693	-1.3353	C	3.3779	-0.8436	-1.3647
C	-2.8495	-0.6531	1.8432	C	-2.9567	-1.1	1.5653
O	-3.3312	0.2605	2.4936	O	-3.484	-0.3853	2.4026
O	-2.726	-1.8677	2.4158	O	-2.8361	-2.4195	1.8159
C	0.1582	-0.0484	-1.8969	C	0.203	0.4577	-1.7498
C	-2.6595	0.8236	-0.1302	C	-2.7068	0.8317	0.043
O	-4.0808	0.9427	-0.3261	O	-4.1199	0.9869	-0.1841
C	-4.4884	2.1663	-0.7617	C	-4.5308	2.2782	-0.3137
O	-3.7515	3.1033	-1.0333	O	-3.8003	3.2585	-0.3027
C	-5.9813	2.1982	-0.8761	C	-6.0174	2.3278	-0.4877
C	5.9288	1.2272	-0.074	C	5.8408	1.2774	0.6571
C	5.6463	2.729	-0.1639	C	7.075	0.376	0.7413
C	6.7028	0.8928	1.2035	C	6.096	2.4666	-0.2725
O	1.199	-1.887	-0.6059	O	1.2197	-1.6417	-0.9195
H	-0.4526	-2.5189	-2.5836	H	-0.3297	-1.7608	-3.0698
H	-0.8083	-3.2381	-1.0384	H	-0.7403	-2.8521	-1.7769
H	-0.6083	-1.727	1.1011	H	-0.6648	-1.934	0.6833
H	-2.4917	-1.0711	-2.4118	H	-2.4014	-0.4187	-2.6341
H	-2.8752	-2.7749	-2.258	H	-2.7591	-2.1081	-2.9366
H	-2.9612	-2.6508	0.1455	H	-2.9546	-2.6004	-0.5873
H	-4.0652	-1.4898	-0.5317	H	-4.0491	-1.313	-0.9997
H	0.0676	1.2063	0.6302	H	-0.0241	1.0272	1.0057
H	-0.5733	0.5645	2.1266	H	-0.7188	0.0212	2.2575

H	1.3417	-1.0681	2.2701	H	1.2184	-1.5809	2.0736
H	1.7088	0.63	2.4699	H	1.544	0.013	2.7153
H	3.7291	1.229	1.5865	H	3.5916	0.8314	2.1111
H	5.2942	-0.6509	-1.9589	H	5.3462	-0.0747	-1.717
H	3.2339	-1.9855	-2.1456	H	3.3225	-1.3306	-2.3345
H	-3.1023	-1.7484	3.3135	H	-3.2543	-2.5352	2.6954
H	-0.7678	0.3115	-2.3448	H	-0.7088	0.9133	-2.1356
H	0.665	0.813	-1.4535	H	0.6731	1.1814	-1.0784
H	0.7922	-0.3817	-2.7282	H	0.8796	0.3514	-2.6071
H	-2.3362	1.6079	0.5655	H	-2.4297	1.4153	0.9297
H	-2.1846	1.0099	-1.0922	H	-2.1931	1.26	-0.8162
H	-6.4322	1.99	0.0975	H	-6.5073	1.8754	0.3782
H	-6.2988	3.1936	-1.1998	H	-6.3389	3.3705	-0.5628
H	-6.3115	1.467	-1.6179	H	-6.3003	1.8073	-1.406
H	6.579	0.9725	-0.9215	H	5.6711	1.6869	1.6617
H	6.5832	3.2966	-0.1723	H	7.9356	0.9365	1.1223
H	5.1	2.9702	-1.0826	H	6.8982	-0.4676	1.4179
H	5.0498	3.086	0.6826	H	7.3513	-0.0329	-0.2367
H	7.6583	1.428	1.2257	H	6.9357	3.0674	0.0934
H	6.9181	-0.18	1.2612	H	5.2162	3.1175	-0.3258
H	6.1455	1.1701	2.1049	H	6.3366	2.1463	-1.292
**Conf 3**	**X axis(Å)**	**Y axis(Å)**	**Z axis(Å)**	**Conf 4**	**X axis(Å)**	**Y axis(Å)**	**Z axis(Å)**
C	-2.718	-2.3245	-1.6191	C	-2.5967	-2.221	-1.8621
C	-3.3859	-1.8124	-0.3501	C	-3.3441	-1.8332	-0.5934
C	-2.674	-0.5803	0.2699	C	-2.7059	-0.635	0.1591
C	-1.1346	-0.8904	0.454	C	-1.1681	-0.9139	0.3989
C	-0.4156	-1.4758	-0.8069	C	-0.3676	-1.374	-0.8648
C	-1.2548	-2.6531	-1.3689	C	-1.1378	-2.5258	-1.5619
C	-0.3499	0.26	1.1309	C	-0.4572	0.2005	1.2053
C	0.9733	-0.1851	1.753	C	0.8472	-0.2546	1.8592
C	2.0562	-0.4138	0.7378	C	1.987	-0.369	0.8878
C	1.9269	-1.3759	-0.2617	C	1.9399	-1.2502	-0.1905
C	3.2567	0.3138	0.8186	C	3.1574	0.3838	1.0894
C	4.3193	0.08	-0.0709	C	4.2706	0.2536	0.2419
C	4.1702	-0.9174	-1.0421	C	4.2034	-0.6655	-0.8125
C	2.9854	-1.6476	-1.1283	C	3.049	-1.42	-1.0189
C	-3.3395	-0.3765	1.6392	C	-3.4456	-0.5624	1.5033
O	-4.1441	0.4885	1.9459	O	-4.293	0.2509	1.8349
O	-3.0021	-1.3074	2.5544	O	-3.1236	-1.5541	2.3582
C	-0.1479	-0.478	-1.9546	C	-0.0759	-0.2791	-1.914
C	-3.0328	0.6653	-0.583	C	-3.0628	0.6645	-0.6099
O	-2.3473	1.8407	-0.1383	O	-2.4405	1.8196	-0.0375
C	-2.5855	2.9532	-0.8858	C	-2.6789	2.9817	-0.7053
O	-3.2878	2.999	-1.8853	O	-3.3318	3.0877	-1.7334
C	-1.8451	4.1217	-0.3121	C	-2.0084	4.1213	-0.0021
C	5.6169	0.8645	0.0118	C	5.5141	1.0876	0.4952
C	5.386	2.3616	-0.2088	C	6.7172	0.209	0.8466
C	6.3412	0.6133	1.3366	C	5.8326	1.9994	-0.6924
O	0.8112	-2.1473	-0.3995	O	0.8588	-2.0401	-0.4478
H	-0.799	-3.0281	-2.2949	H	-0.6238	-2.8111	-2.4895
H	-1.2016	-3.4991	-0.6684	H	-1.0915	-3.424	-0.929
H	-1.0888	-1.7124	1.1862	H	-1.1316	-1.7907	1.065
H	-2.8196	-1.6003	-2.4331	H	-3.0669	-3.1123	-2.2949
H	-3.2362	-3.2311	-1.9542	H	-2.6814	-1.4365	-2.6201
H	-3.4111	-2.6363	0.3763	H	-3.3782	-2.7136	0.063
H	-4.4374	-1.5786	-0.5632	H	-4.3908	-1.6139	-0.8423
H	-0.1588	1.0797	0.4319	H	-0.2587	1.0792	0.5845
H	-0.9554	0.6764	1.9447	H	-1.1163	0.5318	2.0164
H	0.8414	-1.0924	2.3553	H	0.7154	-1.211	2.38
H	1.2928	0.5958	2.4551	H	1.1048	0.4761	2.6367
H	3.3689	1.0672	1.5961	H	3.2065	1.0754	1.929

H	4.9766	-1.1391	-1.7371	H	5.0497	-0.808	-1.4797
H	2.8818	-2.4256	-1.8796	H	3.0091	-2.1372	-1.834
H	-3.518	-1.0578	3.3502	H	-3.6869	-1.3851	3.1431
H	-1.0551	-0.1432	-2.4554	H	-0.9676	0.0686	-2.4337
H	0.3879	0.414	-1.6175	H	0.4122	0.5979	-1.4794
H	0.4814	-0.9412	-2.7246	H	0.6063	-0.6599	-2.684
H	-2.8007	0.5008	-1.6365	H	-2.7725	0.5928	-1.6594
H	-4.1131	0.8596	-0.5315	H	-4.1503	0.822	-0.6004
H	-2.062	5.0148	-0.905	H	-0.9266	3.9665	0.0048
H	-2.172	4.2994	0.7156	H	-2.2258	5.0528	-0.5324
H	-0.7695	3.9312	-0.3465	H	-2.392	4.2047	1.0179
H	6.2836	0.5216	-0.7907	H	5.3276	1.7398	1.3588
H	6.3405	2.8985	-0.2327	H	7.5829	0.8279	1.1063
H	4.8756	2.5427	-1.1614	H	6.4949	-0.4338	1.7057
H	4.7761	2.8045	0.586	H	7.0096	-0.4381	0.0125
H	7.3125	1.1196	1.3461	H	6.6793	2.6539	-0.4585
H	6.5196	-0.4571	1.4884	H	4.975	2.6357	-0.9383
H	5.7667	0.9805	2.1939	H	6.0927	1.4282	-1.5902
**Conf 5**	**X axis(Å)**	**Y axis(Å)**	**Z axis(Å)**	**Conf 6**	**X axis(Å)**	**Y axis(Å)**	**Z axis(Å)**
C	-2.619	-2.4869	-1.4922	C	-2.4974	-2.4128	-1.7111
C	-3.3218	-1.8918	-0.2833	C	-3.2752	-1.93	-0.498
C	-2.651	-0.6043	0.2656	C	-2.6791	-0.6619	0.1695
C	-1.1029	-0.8618	0.4874	C	-1.1336	-0.8798	0.445
C	-0.3528	-1.5595	-0.6993	C	-0.3056	-1.4649	-0.7508
C	-1.1668	-2.7907	-1.1689	C	-1.0503	-2.6874	-1.3422
C	-0.3409	0.3757	1.0265	C	-0.4441	0.3424	1.1034
C	0.9806	0.0293	1.7116	C	0.8582	-0.005	1.8239
C	2.0826	-0.3111	0.7494	C	2.014	-0.2376	0.8933
C	1.9788	-1.387	-0.1298	C	1.9903	-1.2514	-0.0624
C	3.2787	0.4283	0.7671	C	3.1793	0.5396	1.0184
C	4.361	0.0969	-0.0658	C	4.3093	0.3061	0.2164
C	4.2366	-1.0103	-0.9135	C	4.2645	-0.7416	-0.7115
C	3.0567	-1.7532	-0.936	C	3.1158	-1.5215	-0.8407
C	-3.3206	-0.3731	1.6287	C	-3.4166	-0.5517	1.5125
O	-2.8236	-0.5377	2.7313	O	-2.9627	-0.7763	2.6232
O	-4.6175	-0.0125	1.5448	O	-4.7214	-0.2321	1.3934
C	-0.067	-0.6763	-1.9355	C	4.00E-04	-0.4867	-1.9082
C	-3.0096	0.5911	-0.65	C	-3.0426	0.5821	-0.6768
O	-2.4931	1.8269	-0.1391	O	-2.5973	1.7959	-0.0578
C	-2.6818	2.9015	-0.9536	C	-2.7919	2.9184	-0.8034
O	-3.2379	2.8769	-2.0417	O	-3.2985	2.9515	-1.9153
C	-2.0999	4.1289	-0.3228	C	-2.2866	4.1178	-0.0625
C	5.6537	0.8935	-0.0499	C	5.5462	1.1708	0.3845
C	5.4221	2.3528	-0.4503	C	6.7428	0.35	0.8713
C	6.351	0.8045	1.3097	C	5.8881	1.9222	-0.9047
O	0.8692	-2.1761	-0.2016	O	0.9159	-2.0718	-0.2404
H	-0.6833	-3.2488	-2.042	H	-0.5115	-3.0652	-2.2213
H	-1.1323	-3.5664	-0.3904	H	-1.0205	-3.5147	-0.6184
H	-1.0536	-1.5964	1.3084	H	-1.0923	-1.6686	1.2145
H	-2.6992	-1.8182	-2.3547	H	-2.565	-1.6877	-2.528
H	-3.1246	-3.4169	-1.7785	H	-2.9538	-3.3373	-2.0845
H	-3.3532	-2.6542	0.5082	H	-3.3121	-2.7474	0.2365
H	-4.3706	-1.6978	-0.5443	H	-4.3181	-1.7551	-0.7936
H	-0.1621	1.109	0.2336	H	-0.2588	1.1358	0.3724
H	-0.955	0.8826	1.7791	H	-1.1098	0.7727	1.8598
H	0.8511	-0.7969	2.4215	H	0.7291	-0.8834	2.4683
H	1.281	0.8961	2.3144	H	1.0982	0.827	2.4986
H	3.3718	1.269	1.4523	H	3.2112	1.3329	1.7635
H	5.0584	-1.3084	-1.5601	H	5.1241	-0.9656	-1.3382
H	2.972	-2.6159	-1.5908	H	3.0931	-2.338	-1.5571
H	-4.9014	0.0628	2.481	H	-5.049	-0.2335	2.3182

H	-0.9668	-0.3921	-2.4797	H	-0.8847	-0.1964	-2.4728
H	0.4661	0.2442	-1.6823	H	0.4863	0.4317	-1.5673
H	0.5728	-1.2127	-2.6473	H	0.6911	-0.9485	-2.6247
H	-2.6273	0.4357	-1.6597	H	-2.6102	0.5124	-1.6758
H	-4.0987	0.6949	-0.7413	H	-4.1298	0.6535	-0.8117
H	-1.0248	3.9961	-0.1778	H	-1.2145	4.0136	0.1229
H	-2.26	4.9862	-0.9828	H	-2.4508	5.0137	-0.6679
H	-2.5958	4.326	0.6309	H	-2.8312	4.2286	0.8785
H	6.3381	0.4625	-0.7927	H	5.341	1.9289	1.1521
H	6.3751	2.8887	-0.518	H	7.6023	1.0007	1.0659
H	4.931	2.4173	-1.4277	H	6.5036	-0.1767	1.8019
H	4.7943	2.8827	0.2743	H	7.0531	-0.3989	0.1344
H	7.3201	1.3144	1.2792	H	6.7289	2.6048	-0.7405
H	6.5302	-0.2394	1.5902	H	5.035	2.5177	-1.2486
H	5.7573	1.2666	2.1059	H	6.1676	1.2403	-1.7152
**Conf 7**	**X axis(Å)**	**Y axis(Å)**	**Z axis(Å)**	**Conf 8**	**X axis(Å)**	**Y axis(Å)**	**Z axis(Å)**
C	-2.3999	-1.7331	-1.8943	C	-2.336	-2.0916	-1.6289
C	-3.0104	-1.6447	-0.5073	C	-2.9798	-1.7476	-0.296
C	-2.2963	-0.6373	0.4263	C	-2.2924	-0.5646	0.44
C	-0.7364	-0.9059	0.4304	C	-0.7344	-0.8063	0.5275
C	-0.0811	-1.108	-0.9806	C	-0.0412	-1.2774	-0.7976
C	-0.9302	-2.1115	-1.7996	C	-0.8653	-2.4257	-1.4313
C	0.0301	0.1193	1.3048	C	0.0079	0.3733	1.2041
C	1.4193	-0.3604	1.7223	C	1.3938	0.0034	1.7326
C	2.4388	-0.3226	0.6193	C	2.4383	-0.1753	0.6687
C	2.2826	-1.0568	-0.5555	C	2.3116	-1.1359	-0.3333
C	3.6233	0.4157	0.795	C	3.6175	0.5901	0.7172
C	4.6427	0.4155	-0.1724	C	4.6605	0.3964	-0.2048
C	4.4689	-0.3597	-1.3248	C	4.5158	-0.6007	-1.1765
C	3.301	-1.0989	-1.5085	C	3.3531	-1.3682	-1.2322
C	-2.8481	-0.9544	1.8259	C	-2.837	-0.6096	1.8773
O	-2.271	-1.5437	2.7258	O	-2.9746	-1.6127	2.5619
O	-4.1235	-0.5633	2.0148	O	-3.1217	0.5871	2.4218
C	0.1006	0.1595	-1.8494	C	0.1724	-0.1998	-1.8878
C	-2.6233	0.8479	0.1287	C	-2.6681	0.785	-0.2174
O	-3.9447	1.0017	-0.4153	O	-4.0973	0.9004	-0.3558
C	-4.3412	2.2917	-0.5864	C	-4.517	2.0719	-0.9078
O	-3.6625	3.2793	-0.3438	O	-3.7884	2.9721	-1.2993
C	-5.7373	2.3314	-1.1266	C	-6.0128	2.0987	-0.9764
C	5.9214	1.2128	0.015	C	5.9336	1.2226	-0.1553
C	5.6391	2.7128	0.1323	C	5.6475	2.713	-0.356
C	6.7233	0.7125	1.2189	C	6.7063	0.9853	1.1445
O	1.1797	-1.8162	-0.8172	O	1.2133	-1.9353	-0.4599
H	-0.512	-2.2208	-2.809	H	-0.422	-2.7193	-2.3921
H	-0.8456	-3.1114	-1.3505	H	-0.7848	-3.3219	-0.7996
H	-0.603	-1.8751	0.9379	H	-0.6122	-1.6512	1.2257
H	-2.5367	-0.7909	-2.4337	H	-2.4675	-1.2746	-2.3447
H	-2.93	-2.4994	-2.4722	H	-2.8455	-2.962	-2.0593
H	-2.9706	-2.6464	-0.0546	H	-2.9407	-2.6513	0.328
H	-4.0803	-1.42	-0.5959	H	-4.0488	-1.553	-0.4452
H	0.0974	1.0938	0.809	H	0.0727	1.2377	0.5345
H	-0.5299	0.2963	2.2305	H	-0.5671	0.7093	2.0739
H	1.3733	-1.3767	2.1331	H	1.345	-0.9085	2.3409
H	1.7557	0.2788	2.549	H	1.7087	0.8003	2.4189
H	3.7588	0.9903	1.7097	H	3.7305	1.3413	1.497
H	5.2424	-0.3993	-2.0881	H	5.3078	-0.7921	-1.8965
H	3.1776	-1.7026	-2.4034	H	3.2516	-2.1434	-1.9867
H	-4.3341	-0.8659	2.9237	H	-3.432	0.3659	3.3258
H	-0.8434	0.5773	-2.2024	H	-0.7534	0.129	-2.359
H	0.6377	0.9562	-1.3272	H	0.6811	0.6908	-1.5088
H	0.693	-0.0724	-2.7433	H	0.805	-0.5933	-2.6934

H	-2.5446	1.4444	1.0476	H	-2.3016	1.6341	0.3716
H	-1.9279	1.2722	-0.5949	H	-2.2421	0.8642	-1.2168
H	-6.4192	1.841	-0.4275	H	-6.4316	2.0097	0.0291
H	-6.0505	3.3726	-1.2455	H	-6.3389	3.0503	-1.4058
H	-5.7703	1.843	-2.1037	H	-6.3688	1.2868	-1.6154
H	6.5521	1.0743	-0.8732	H	6.5859	0.9078	-0.9808
H	6.5763	3.2779	0.1786	H	6.5829	3.2809	-0.4049
H	5.0727	3.0733	-0.7337	H	5.1021	2.8843	-1.2908
H	5.0622	2.953	1.0319	H	5.0487	3.1299	0.461
H	7.6798	1.2415	1.2909	H	7.6603	1.5233	1.1287
H	6.9383	-0.3581	1.1285	H	6.9243	-0.0798	1.2815
H	6.1866	0.8664	2.1615	H	6.1467	1.3267	2.0222
**Conf 9**	**X axis(Å)**	**Y axis(Å)**	**Z axis(Å)**	**Conf 10**	**X axis(Å)**	**Y axis(Å)**	**Z axis(Å)**
C	2.3229	-1.1304	2.3269	C	-2.258	-1.5888	-2.1417
C	3.0003	-1.4298	1.0019	C	-2.9659	-1.6127	-0.797
C	2.3446	-0.7219	-0.2087	C	-2.337	-0.6571	0.2541
C	0.7834	-0.9826	-0.2155	C	-0.7792	-0.8975	0.3488
C	0.0588	-0.7858	1.1619	C	-0.0196	-0.9978	-1.0191
C	0.8547	-1.5216	2.2681	C	-0.7901	-1.9481	-1.9691
C	0.0729	-0.2412	-1.3768	C	-0.0928	0.0709	1.3445
C	-1.3008	-0.8194	-1.7133	C	1.2769	-0.4106	1.8227
C	-2.3715	-0.4781	-0.7161	C	2.3699	-0.2936	0.7997
C	-2.281	-0.8569	0.6226	C	2.3074	-0.9591	-0.4235
C	-3.5369	0.181	-1.148	C	3.529	0.4439	1.1022
C	-4.6014	0.4482	-0.2704	C	4.6145	0.5091	0.212
C	-4.4926	0.024	1.0593	C	4.5334	-0.1995	-0.9927
C	-3.3442	-0.6337	1.4982	C	3.3913	-0.937	-1.3017
C	2.9588	-1.4145	-1.4363	C	-2.9416	-1.0827	1.6023
O	2.4179	-2.2309	-2.1651	O	-3.0862	-2.2316	1.9937
O	4.2468	-1.0902	-1.6627	O	-3.2758	-0.0735	2.4266
C	-0.1481	0.673	1.635	C	0.2167	0.33	-1.7784
C	2.676	0.7878	-0.3189	C	-2.7139	0.8138	-0.0447
O	3.9716	1.0879	0.226	O	-4.138	0.947	-0.2153
C	4.376	2.3752	0.0519	C	-4.5595	2.2179	-0.4609
O	3.7225	3.2558	-0.4885	O	-3.8349	3.1966	-0.5685
C	5.745	2.5647	0.6285	C	-6.0512	2.2467	-0.5907
C	-5.8395	1.1641	-0.7811	C	5.8449	1.3197	0.5798
C	-7.0786	0.2693	-0.6993	C	7.0804	0.4283	0.7273
C	-6.0709	2.4855	-0.0438	C	6.1005	2.4444	-0.4267
O	-1.2017	-1.5127	1.1389	O	1.2335	-1.7084	-0.8063
H	0.3872	-1.3468	3.2462	H	-0.2996	-1.9751	-2.9512
H	0.7789	-2.6069	2.1103	H	-0.717	-2.9777	-1.5905
H	0.6621	-2.0548	-0.4403	H	-0.6684	-1.8946	0.8068
H	2.4458	-0.0752	2.59	H	-2.3767	-0.614	-2.6244
H	2.8149	-1.7056	3.1202	H	-2.7292	-2.3211	-2.8081
H	2.9694	-2.5179	0.8434	H	-2.9336	-2.6479	-0.4298
H	4.0675	-1.1882	1.0776	H	-4.0315	-1.3966	-0.9405
H	-0.0057	0.8325	-1.1747	H	-0.0185	1.0812	0.9279
H	0.6788	-0.3276	-2.2863	H	-0.7119	0.1613	2.2437
H	-1.2481	-1.9098	-1.8234	H	1.2222	-1.4505	2.1686
H	-1.589	-0.4352	-2.7004	H	1.5445	0.1817	2.7075
H	-3.6223	0.4797	-2.1918	H	3.593	0.9662	2.0555
H	-5.3013	0.1961	1.7651	H	5.3587	-0.1887	-1.7001
H	-3.2714	-0.9649	2.5305	H	3.3393	-1.4882	-2.2367
H	4.4967	-1.633	-2.4404	H	-3.6196	-0.5271	3.2257
H	0.7832	1.1733	1.9046	H	-0.6951	0.7615	-2.1904
H	-0.6493	1.2928	0.8865	H	0.6888	1.0952	-1.1563
H	-0.7855	0.6977	2.5277	H	0.8918	0.1679	-2.6281
H	2.6491	1.1058	-1.3699	H	-2.3918	1.4825	0.7625
H	1.9522	1.3957	0.2233	H	-2.2473	1.1565	-0.9673
H	6.4534	1.9	0.1276	H	-6.5107	1.8928	0.3356

H	6.0655	3.5982	0.4693	H	-6.3795	3.2743	-0.7712
H	5.725	2.3668	1.7031	H	-6.3614	1.6272	-1.4358
H	-5.6887	1.4106	-1.8405	H	5.6718	1.7953	1.5543
H	-7.9447	0.7695	-1.1462	H	7.9391	1.0147	1.0721
H	-6.919	-0.6707	-1.2392	H	6.9033	-0.3684	1.4584
H	-7.3364	0.02	0.3358	H	7.3599	-0.0447	-0.2204
H	-6.9155	3.0278	-0.4826	H	6.9381	3.07	-0.0997
H	-5.1876	3.1305	-0.11	H	5.2197	3.0886	-0.5257
H	-6.2919	2.33	1.0177	H	6.3444	2.057	-1.4219
**Conf 11**	**X axis(Å)**	**Y axis(Å)**	**Z axis(Å)**	**Conf 12**	**X axis(Å)**	**Y axis(Å)**	**Z axis(Å)**
C	-2.5275	-0.8528	2.112	C	-2.4509	-0.7113	2.1957
C	-3.0847	0.3578	1.3814	C	-3.0798	0.4153	1.3926
C	-2.2898	0.7259	0.1013	C	-2.3456	0.7059	0.0575
C	-0.7453	0.8238	0.4155	C	-0.7959	0.8836	0.303
C	-0.1494	-0.3477	1.2705	C	-0.1255	-0.1926	1.2255
C	-1.0713	-0.6171	2.486	C	-0.9925	-0.3946	2.4933
C	0.0907	1.131	-0.8518	C	-0.0177	1.1159	-1.016
C	1.4783	1.6943	-0.5465	C	1.3572	1.751	-0.8104
C	2.4568	0.6858	-0.0166	C	2.392	0.8243	-0.2396
C	2.2272	-0.0139	1.1672	C	2.2315	0.2155	1.0043
C	3.6787	0.4874	-0.6851	C	3.5963	0.6157	-0.9364
C	4.6627	-0.3803	-0.181	C	4.6301	-0.1726	-0.4028
C	4.4144	-1.0405	1.0279	C	4.4501	-0.7411	0.8638
C	3.2089	-0.8491	1.7017	C	3.2627	-0.5382	1.5658
C	-2.7695	2.1164	-0.3475	C	-2.8939	2.0374	-0.4824
O	-3.1324	2.4462	-1.4653	O	-3.3085	2.262	-1.6083
O	-2.7154	3.0521	0.622	O	-2.8416	3.0501	0.4063
C	0.0698	-1.6978	0.5464	C	0.1196	-1.5886	0.6039
C	-2.6016	-0.2455	-1.0665	C	-2.6613	-0.3679	-1.0159
O	-4.0222	-0.4586	-1.203	O	-4.0766	-0.6414	-1.0801
C	-4.4676	-1.7426	-1.1609	C	-4.4707	-1.9327	-0.9186
O	-3.776	-2.735	-0.9868	O	-3.7358	-2.8826	-0.6926
C	-5.9541	-1.7708	-1.3451	C	-5.9606	-2.0282	-1.0424
C	5.9814	-0.5973	-0.9022	C	5.9119	-0.3723	-1.1921
C	5.7702	-1.1858	-2.2994	C	7.1168	0.2342	-0.4687
C	6.8019	0.6929	-0.9715	C	6.1536	-1.8501	-1.5102
O	1.0814	0.1152	1.8968	O	1.1082	0.363	1.7648
H	-0.6948	-1.4749	3.059	H	-0.5633	-1.189	3.1182
H	-1.0136	0.2324	3.1814	H	-0.9428	0.5103	3.1157
H	-0.6297	1.7172	1.0502	H	-0.6921	1.8292	0.8593
H	-2.6424	-1.7567	1.5069	H	-2.5526	-1.6649	1.6698
H	-3.1091	-1.0189	3.0265	H	-2.9929	-0.8229	3.1424
H	-3.0719	1.1959	2.0908	H	-3.0739	1.3084	2.0316
H	-4.1461	0.1981	1.1581	H	-4.1416	0.2006	1.2239
H	0.1699	0.2501	-1.498	H	0.0722	0.1886	-1.5922
H	-0.4253	1.8944	-1.4458	H	-0.5834	1.8097	-1.6489
H	1.4094	2.5298	0.1616	H	1.2814	2.6384	-0.1695
H	1.8708	2.1262	-1.4763	H	1.6996	2.1193	-1.7863
H	3.8721	1.0303	-1.6087	H	3.7371	1.0875	-1.9077
H	5.1578	-1.7072	1.4585	H	5.2323	-1.3436	1.3187
H	3.0266	-1.3626	2.6418	H	3.1336	-0.9798	2.5502
H	-3.0286	3.8708	0.1823	H	-3.2013	3.8188	-0.0852
H	-0.8592	-2.2135	0.3028	H	-0.7971	-2.155	0.4384
H	0.6401	-1.5925	-0.3806	H	0.652	-1.5391	-0.3498
H	0.6439	-2.3829	1.1828	H	0.7417	-2.1988	1.2705
H	-2.2614	0.1642	-2.0247	H	-2.3713	-0.0258	-2.0162
H	-2.0785	-1.1913	-0.9469	H	-2.0984	-1.2817	-0.8411
H	-6.4387	-1.2357	-0.5247	H	-6.2751	-1.6691	-2.0256
H	-6.2175	-1.3226	-2.3065	H	-6.2665	-3.0734	-0.9407
H	-6.3004	-2.8082	-1.3396	H	-6.4358	-1.4457	-0.2492
H	6.5738	-1.3266	-0.3337	H	5.8157	0.1505	-2.153

H	6.7327	-1.4166	-2.7688	H	8.0172	0.1547	-1.0874
H	5.1904	-2.1143	-2.2495	H	6.9505	1.2957	-0.2536
H	5.2364	-0.4941	-2.96	H	7.3208	-0.2708	0.4817
H	7.7829	0.5014	-1.4197	H	7.0337	-1.9664	-2.1518
H	6.9659	1.1069	0.0297	H	5.2954	-2.2825	-2.0366
H	6.3061	1.4622	-1.5734	H	6.3226	-2.4425	-0.6044

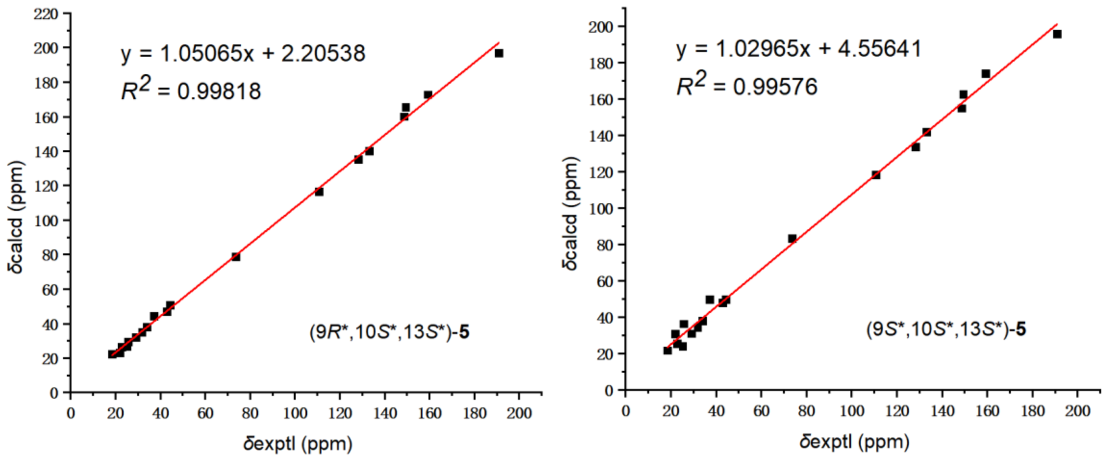

Figure S5. Regression analysis of experimental versus calculated ^{13}C NMR chemical shifts of (9*R*,10*S*,13*S*)-**5** and (9*S*,10*S*,13*S*)-**5** at the mPW1PW91/6-311+G(d,p) level.

Functional	Solvent?	Basis Set	Type of Data			
mPW1PW91	PCM	6-311+G(d,p)	Unscaled Shifts			
	Isomer 1	Isomer 2	Isomer 3	Isomer 4	Isomer 5	Isomer 6
sDP4+ (H data)	100.00%	0.00%	–	–	–	–
sDP4+ (C data)	100.00%	0.00%	–	–	–	–
sDP4+ (all data)	100.00%	0.00%	–	–	–	–
uDP4+ (H data)	10.19%	89.81%	–	–	–	–
uDP4+ (C data)	100.00%	0.00%	–	–	–	–
uDP4+ (all data)	100.00%	0.00%	–	–	–	–
DP4+ (H data)	100.00%	0.00%	–	–	–	–
DP4+ (C data)	100.00%	0.00%	–	–	–	–
DP4+ (all data)	100.00%	0.00%	–	–	–	–

Figure S6. DP4+ results of candidate (9*R*,10*S*,13*S*)-**5** (Isomer 1) and (9*S*,10*S*,13*S*)-**5** (Isomer 2).

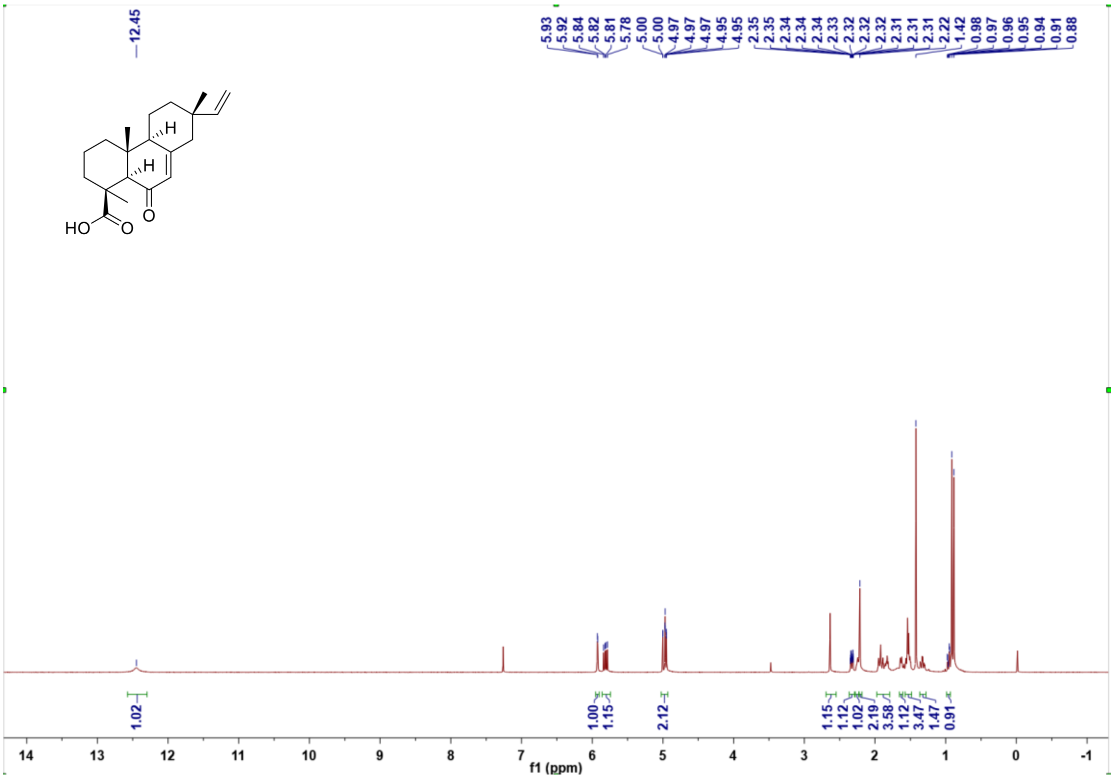

Figure S7. ^{1}H NMR (600 MHz) spectrum of **1** in $CDCl_3$.

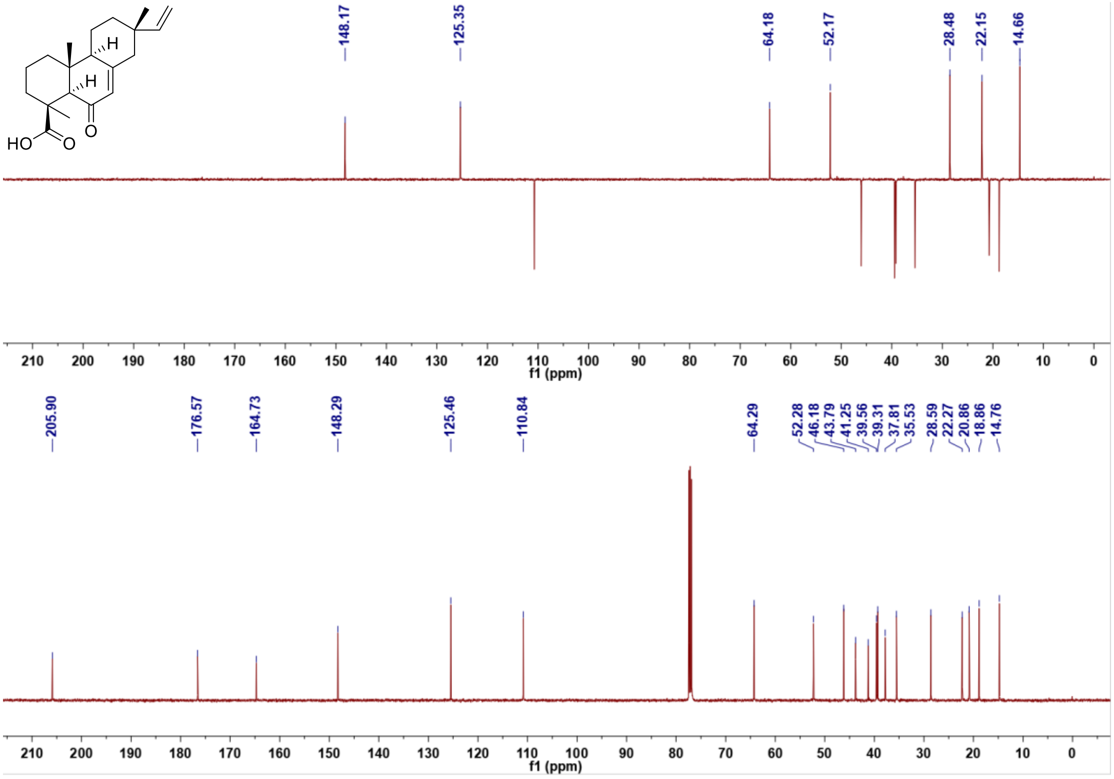

Figure S8. ^{13}C NMR and DEPT (150 MHz) spectra of **1** in $CDCl_3$.

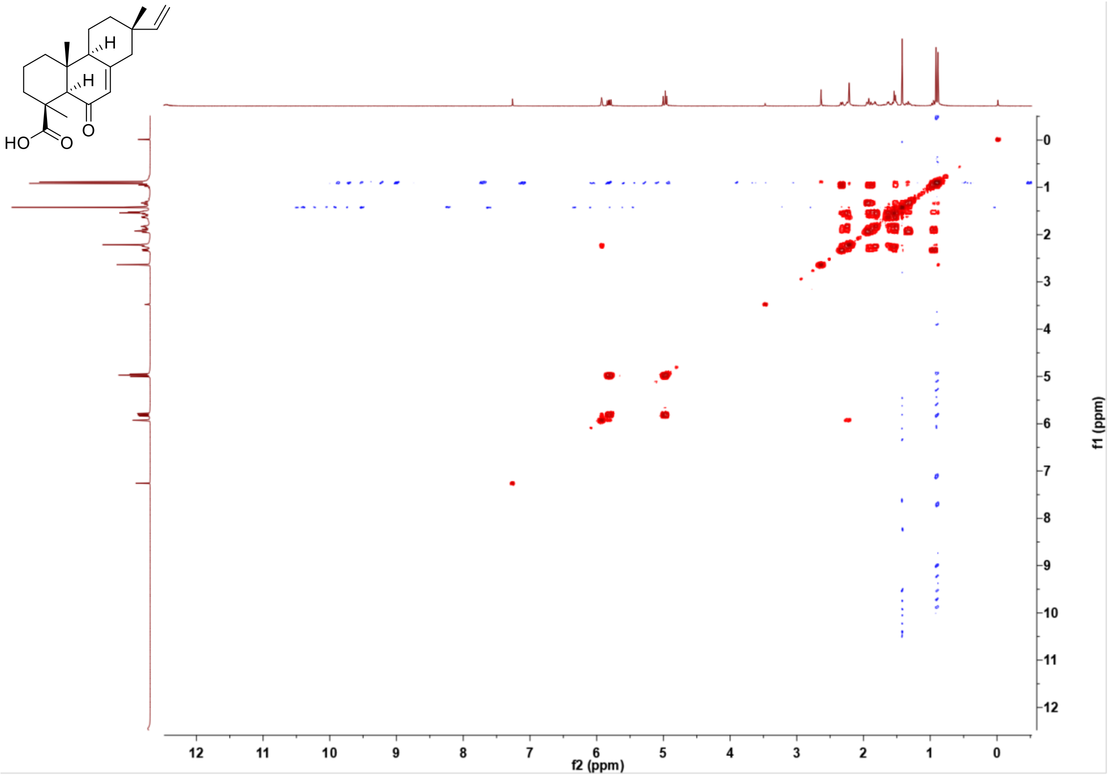

Figure S9. ^{1}H-^{1}H COSY (600 MHz) spectrum of **1** in $CDCl_3$.

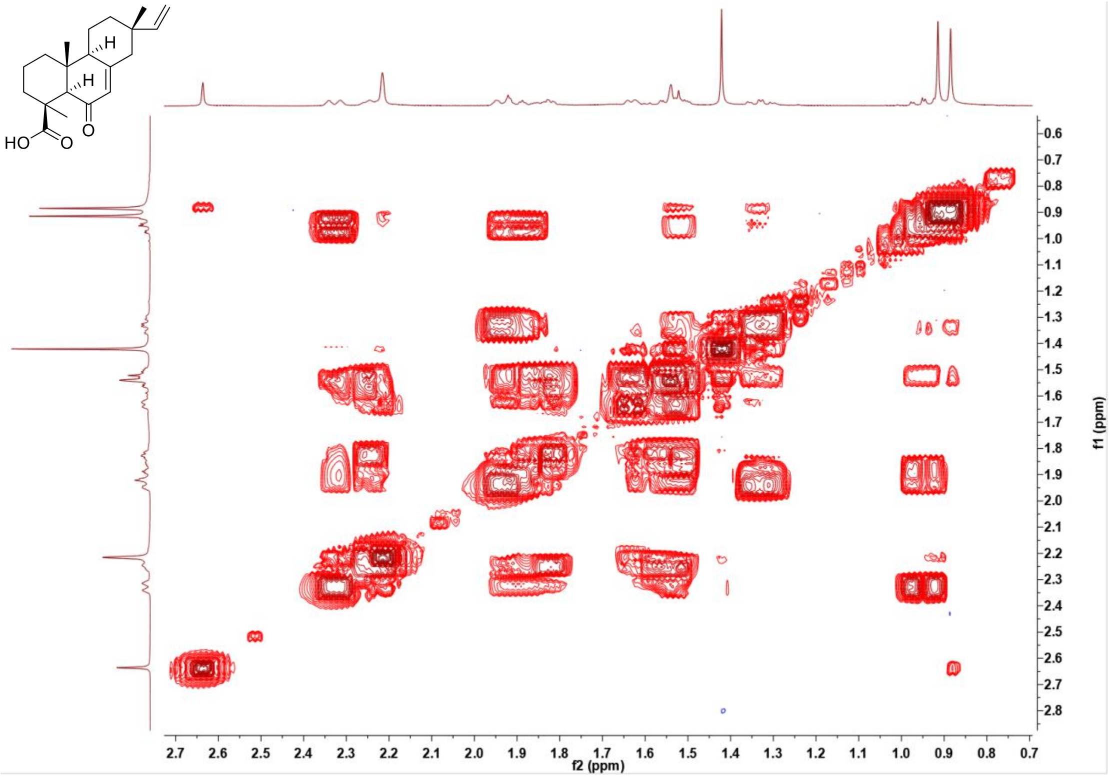

Figure S10. Enlarged ^{1}H-^{1}H COSY (600 MHz) spectrum of **1** in $CDCl_3$.

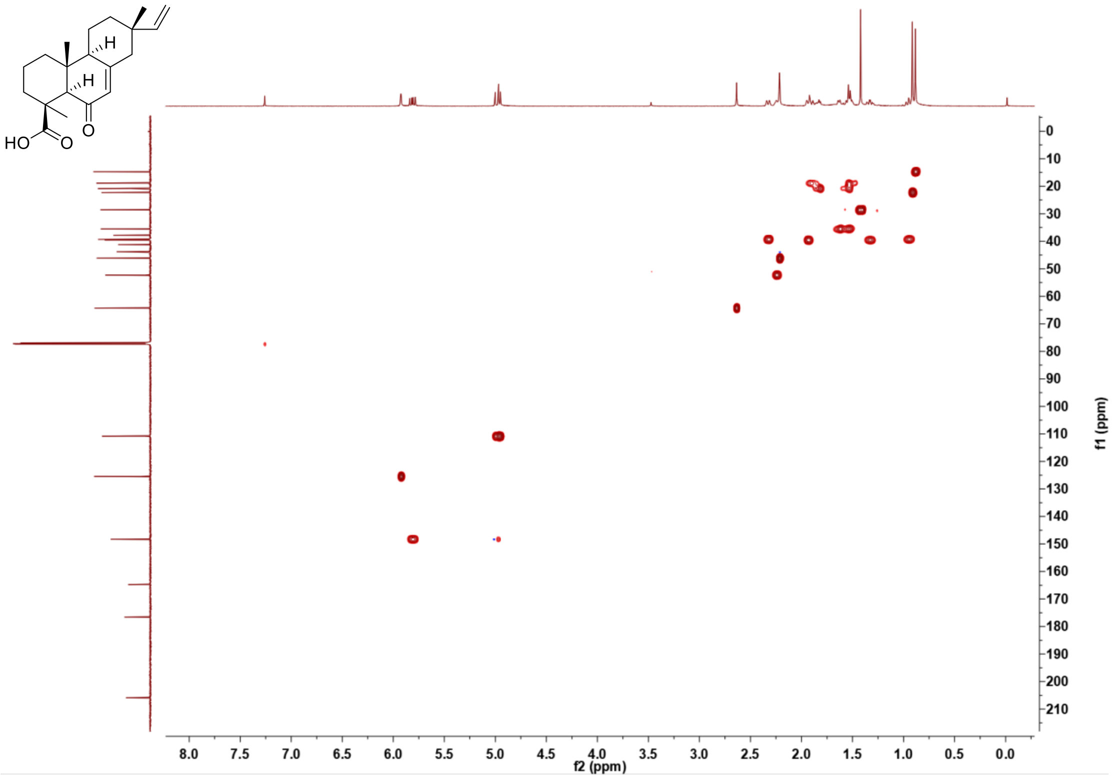

Figure S11. HSQC (600 MHz) spectrum of **1** in $CDCl_3$.

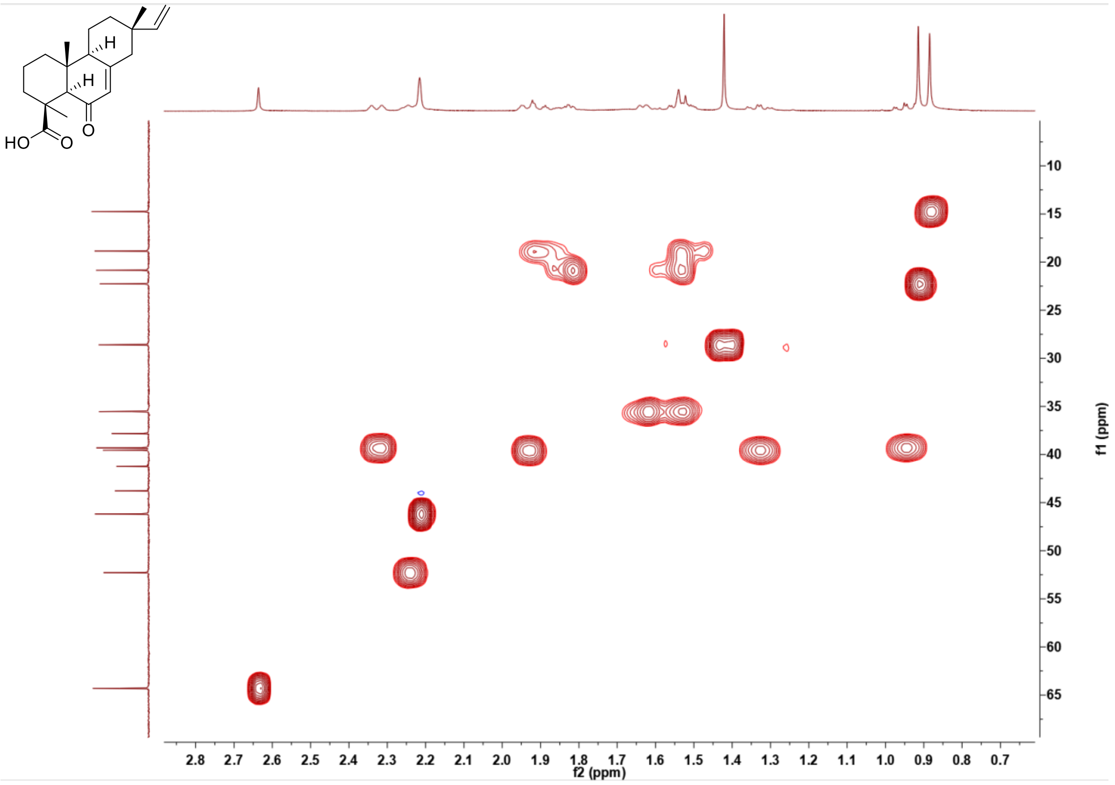

Figure S12. Enlarged HSQC (600 MHz) spectrum of **1** in $CDCl_3$.

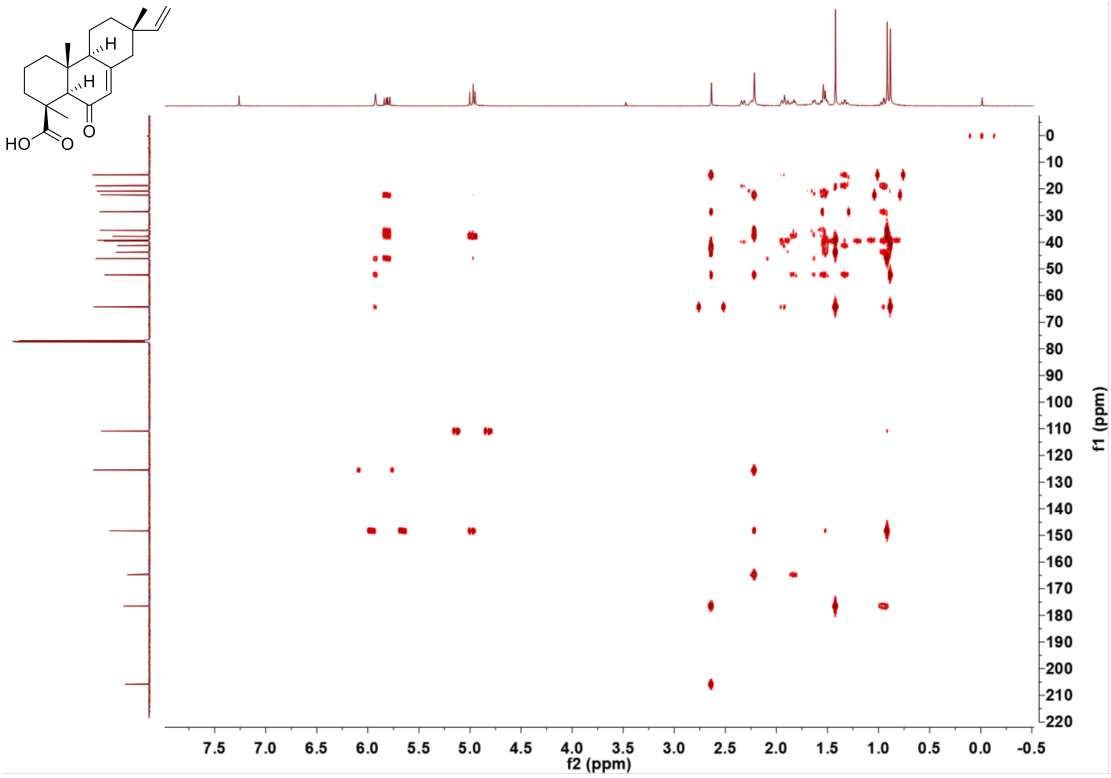

Figure S13. HMBC (600 MHz) spectrum of **1** in $CDCl_3$.

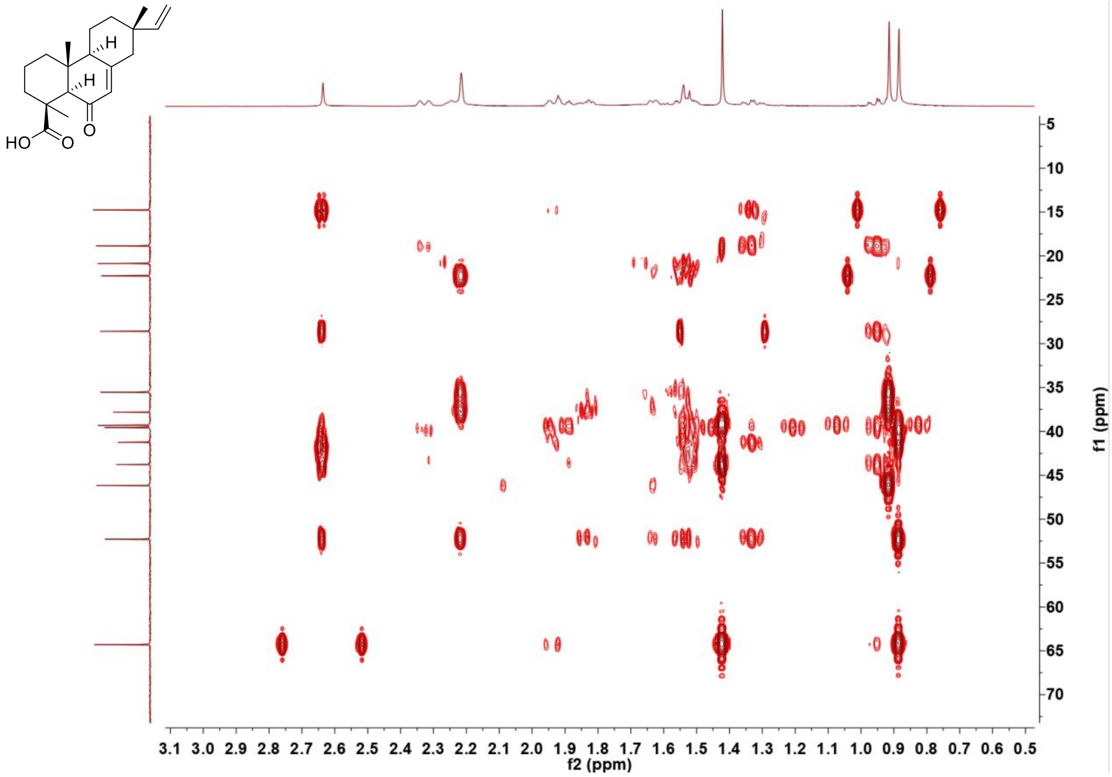

Figure S14. Enlarged HMBC (600 MHz) spectrum of **1** in $CDCl_3$.

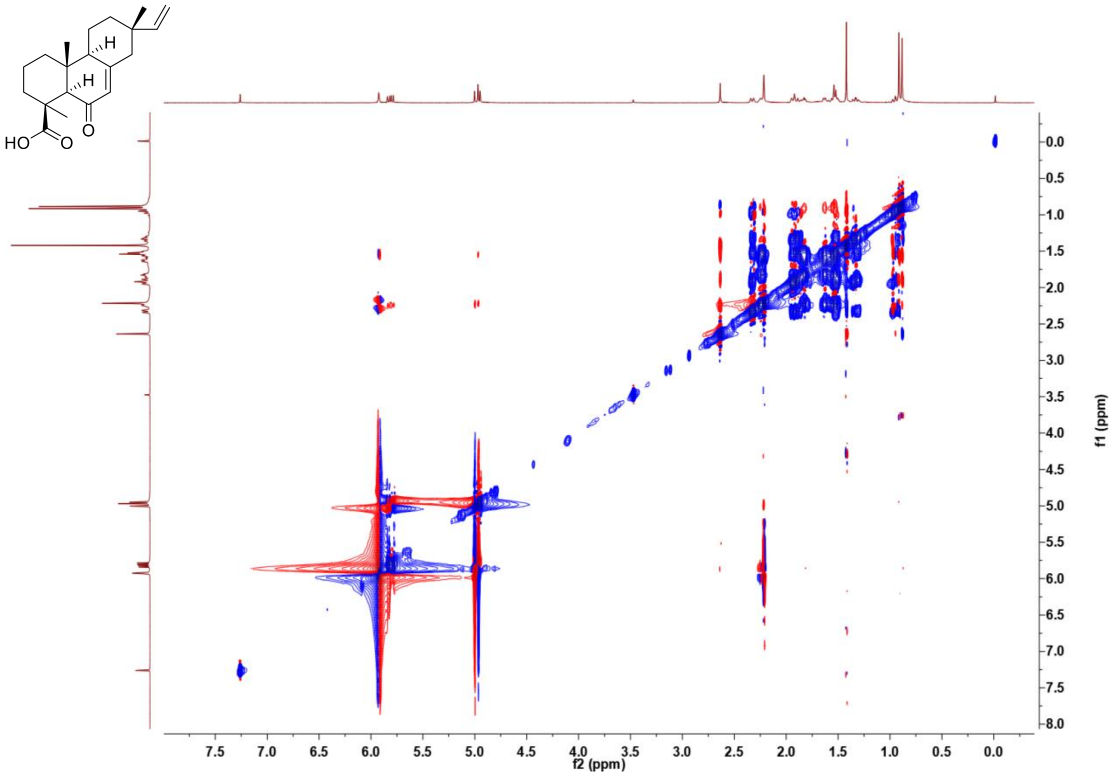

Figure S15. ROESY (600 MHz) spectrum of **1** in $CDCl_3$.

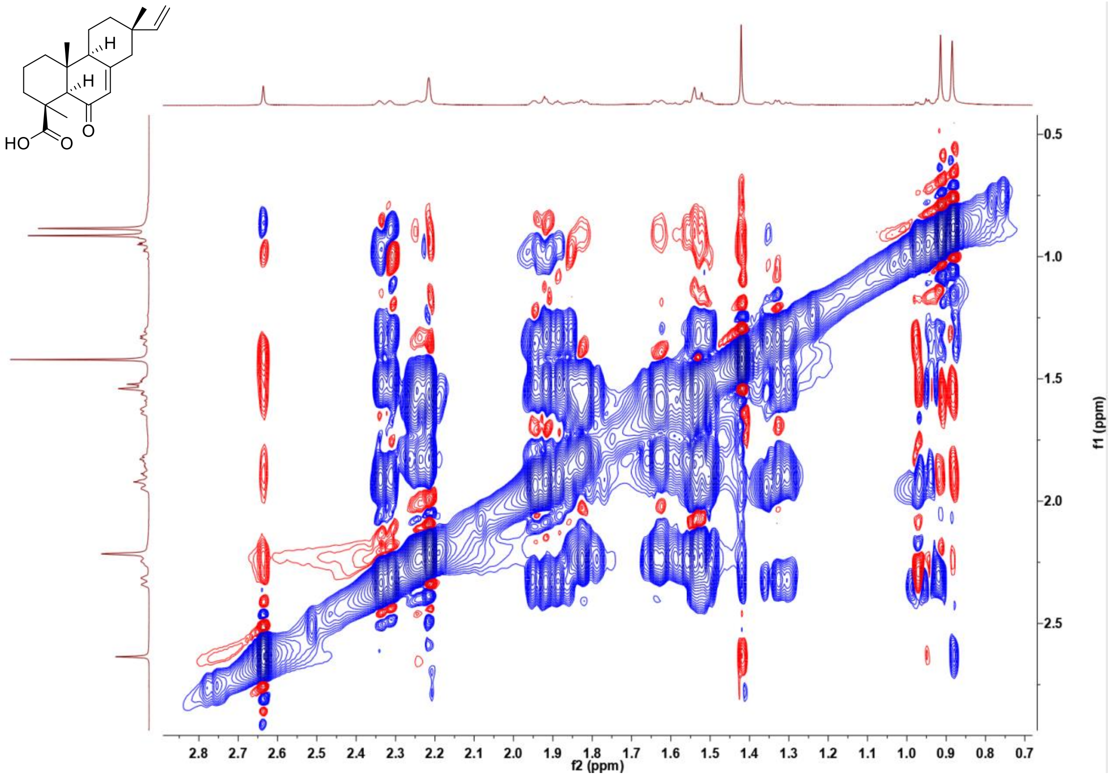

Figure S16. Enlarged ROESY (600 MHz) spectrum of **1** in $CDCl_3$.

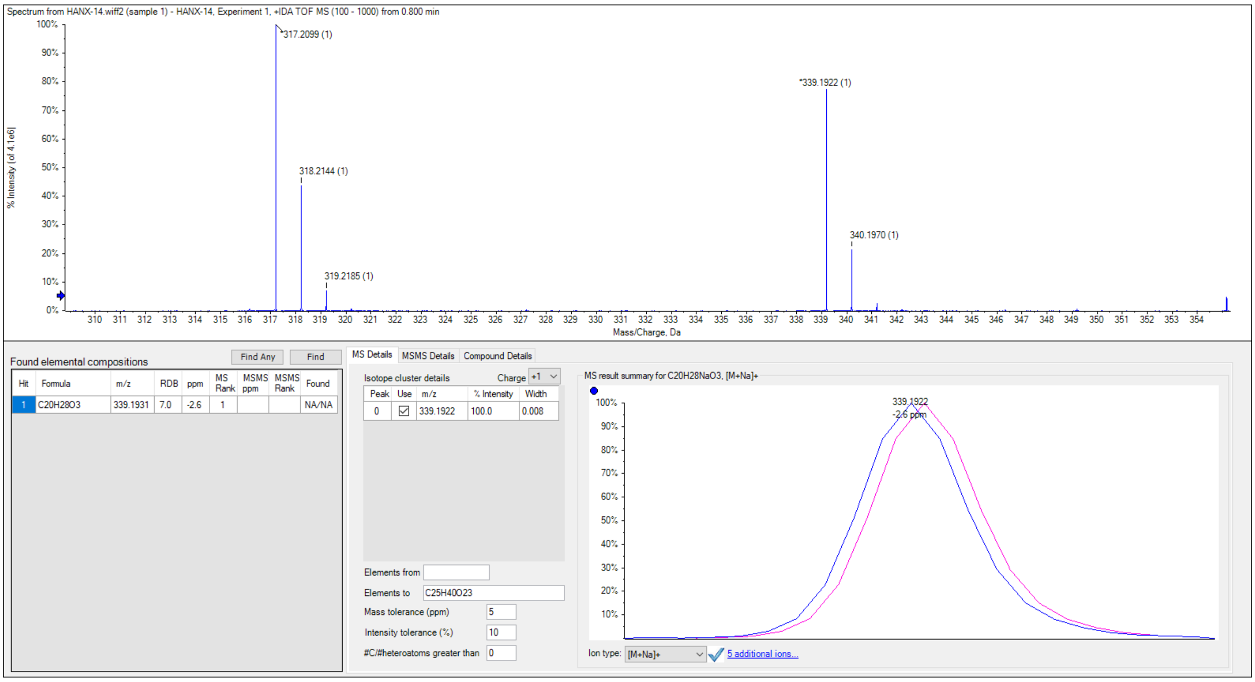

Figure S17. HRESIMS of **1**.

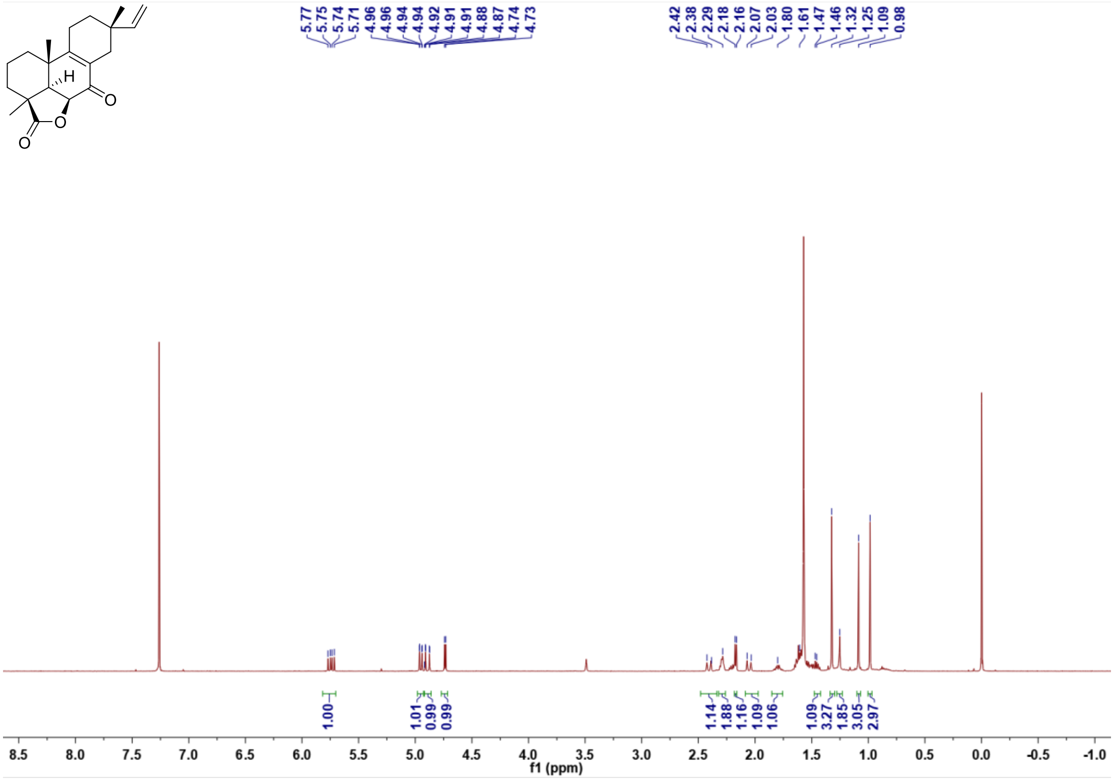

Figure S18. ^{1}H NMR (600 MHz) spectrum of **2** in $CDCl_3$.

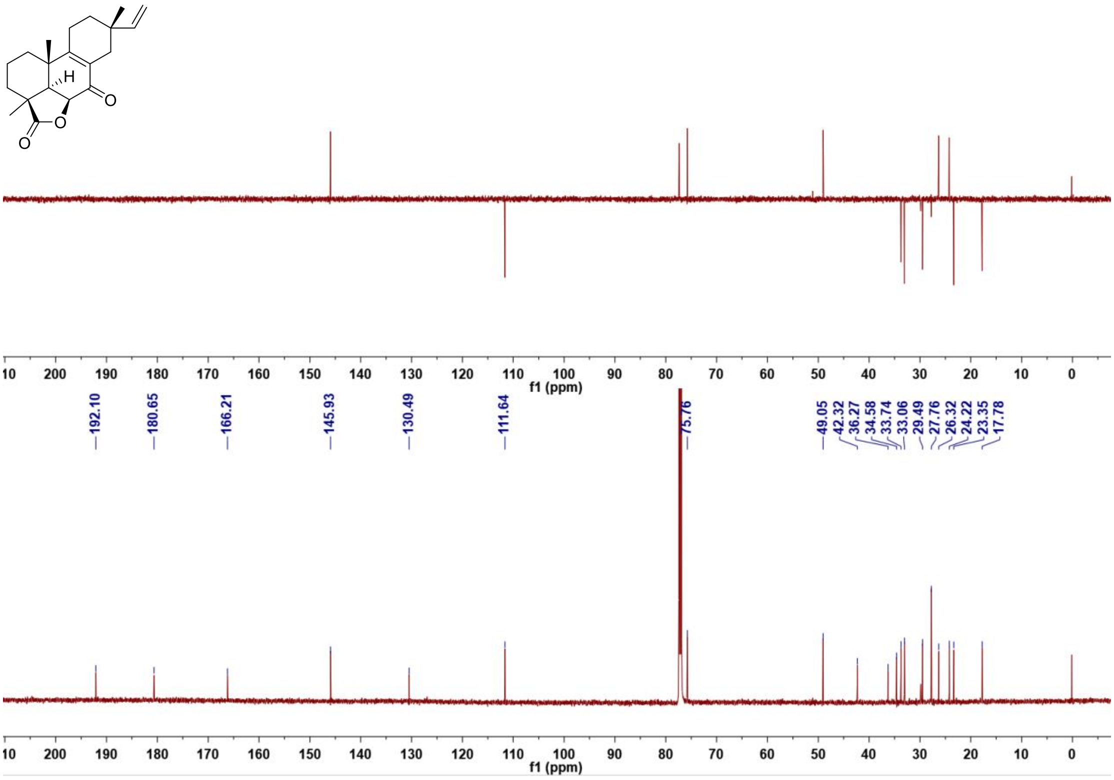

Figure S19. ^{13}C NMR and DEPT (150 MHz) spectra of **2** in $CDCl_3$.

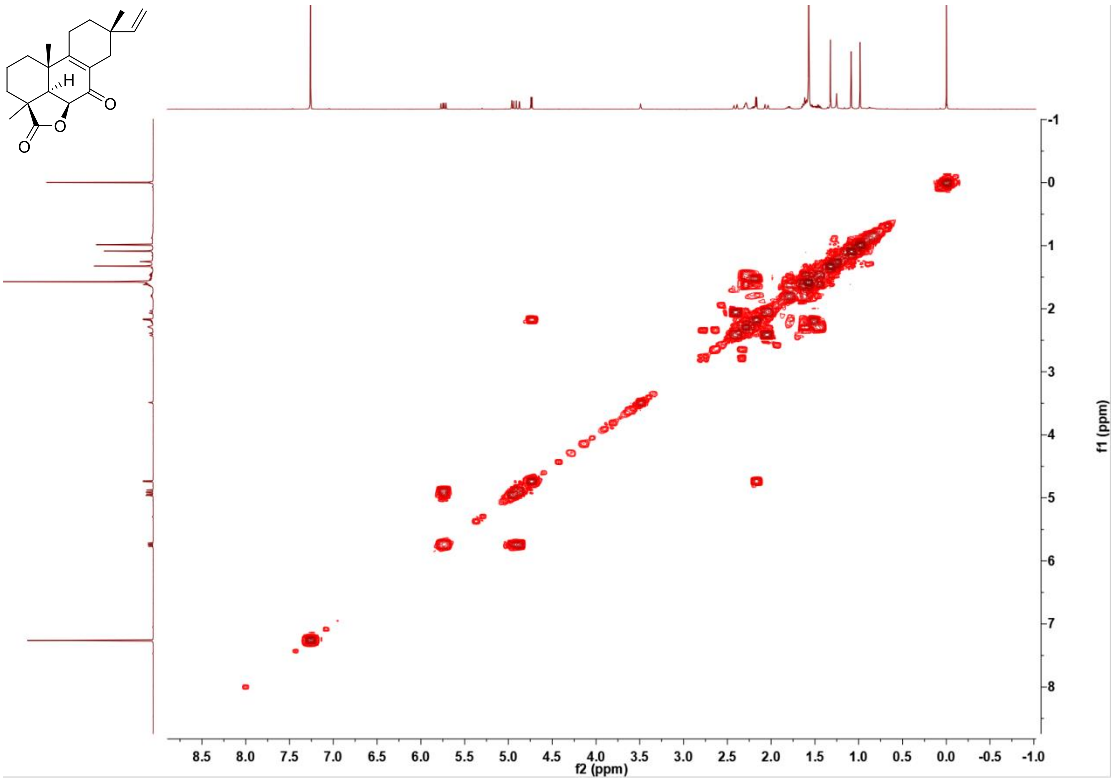

Figure S20. ^{1}H-^{1}H COSY (600 MHz) spectrum of **2** in $CDCl_3$.

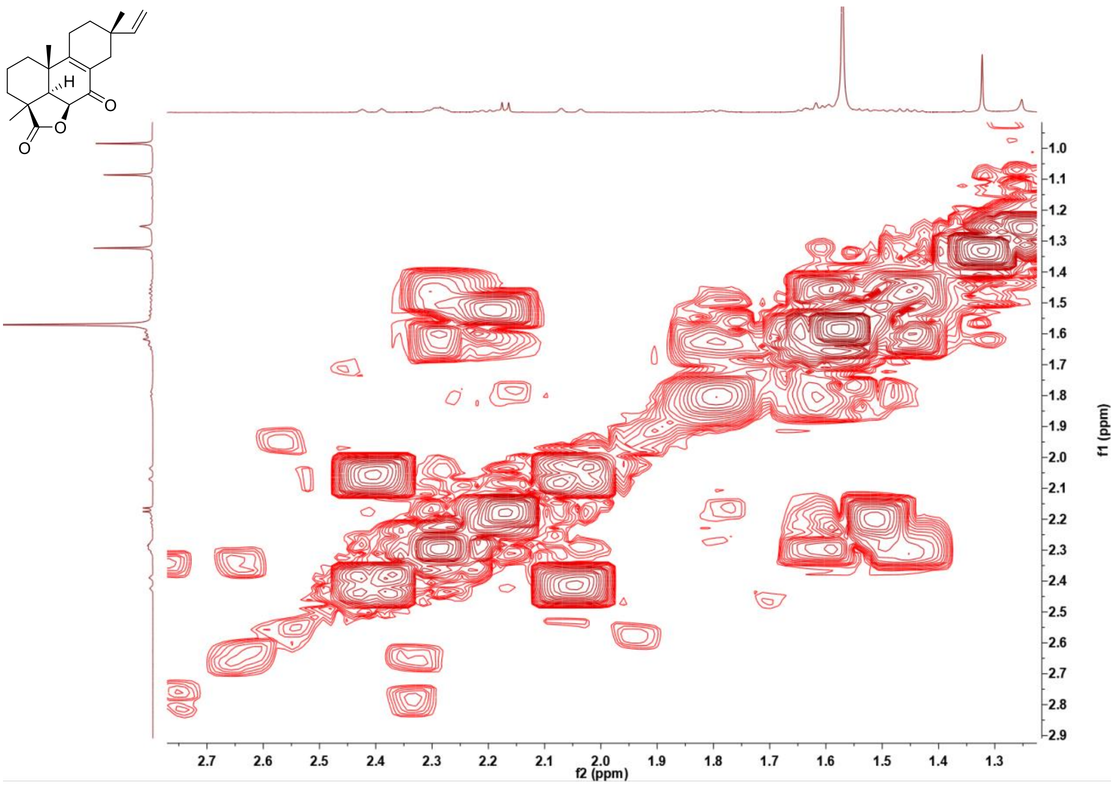

Figure S21. Enlarged ^{1}H-^{1}H COSY (600 MHz) spectrum of **2** in $CDCl_3$.

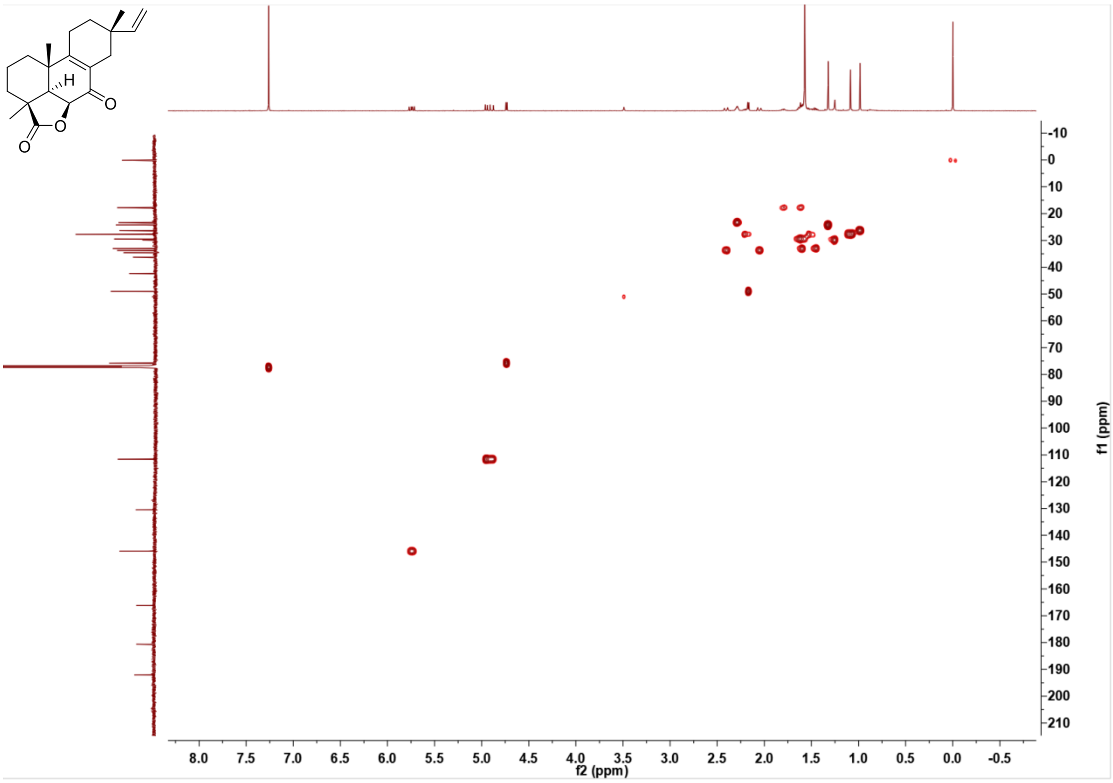

Figure S22. HSQC (600 MHz) spectrum of **2** in $CDCl_3$.

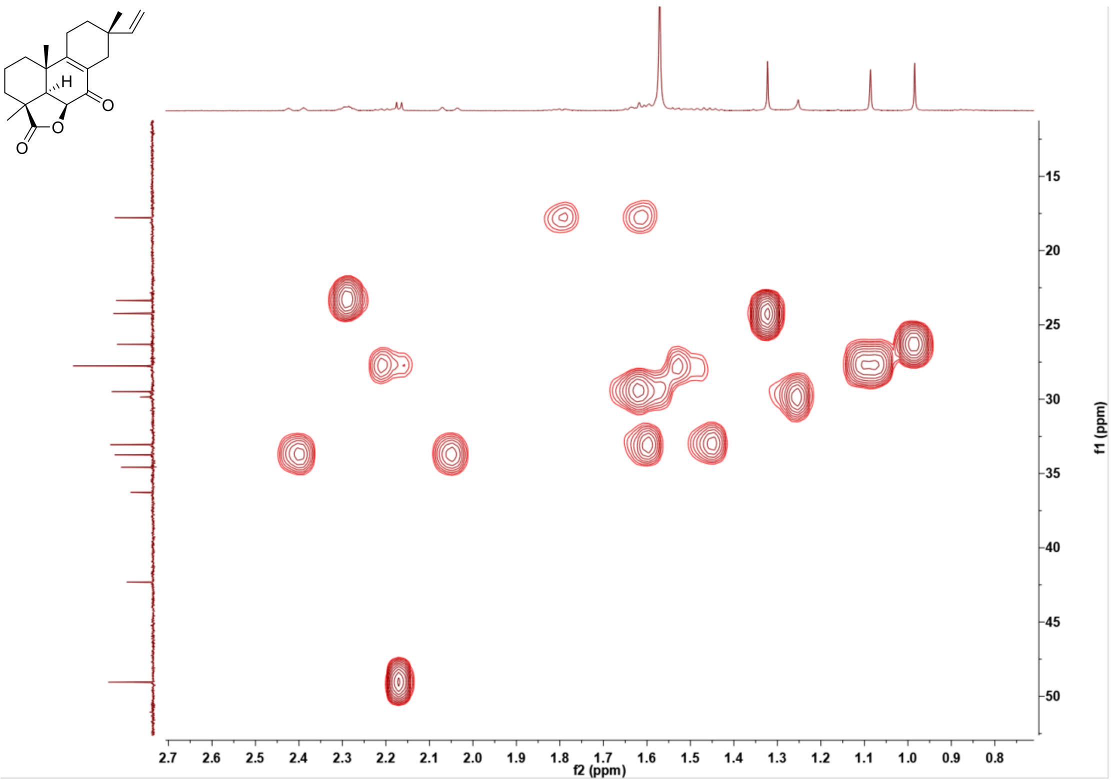

Figure S23. Enlarged HSQC (600 MHz) spectrum of **2** in $CDCl_3$.

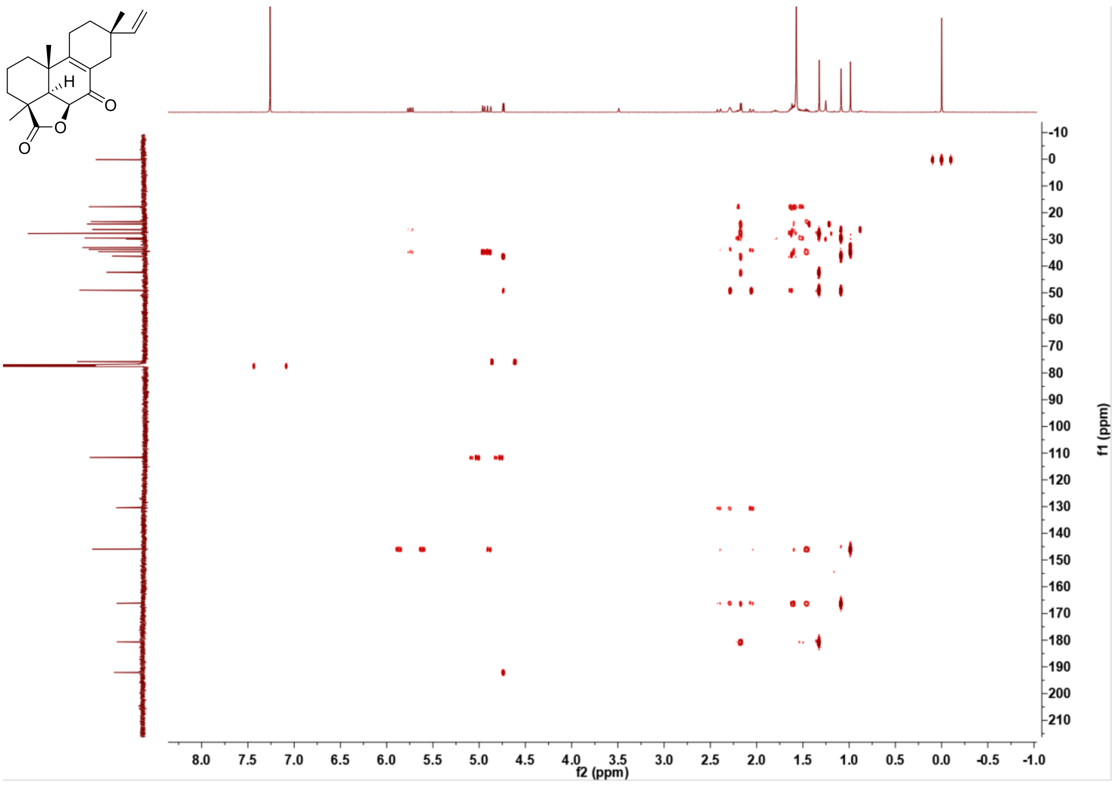

Figure S24. HMBC (600 MHz) spectrum of **2** in $CDCl_3$.

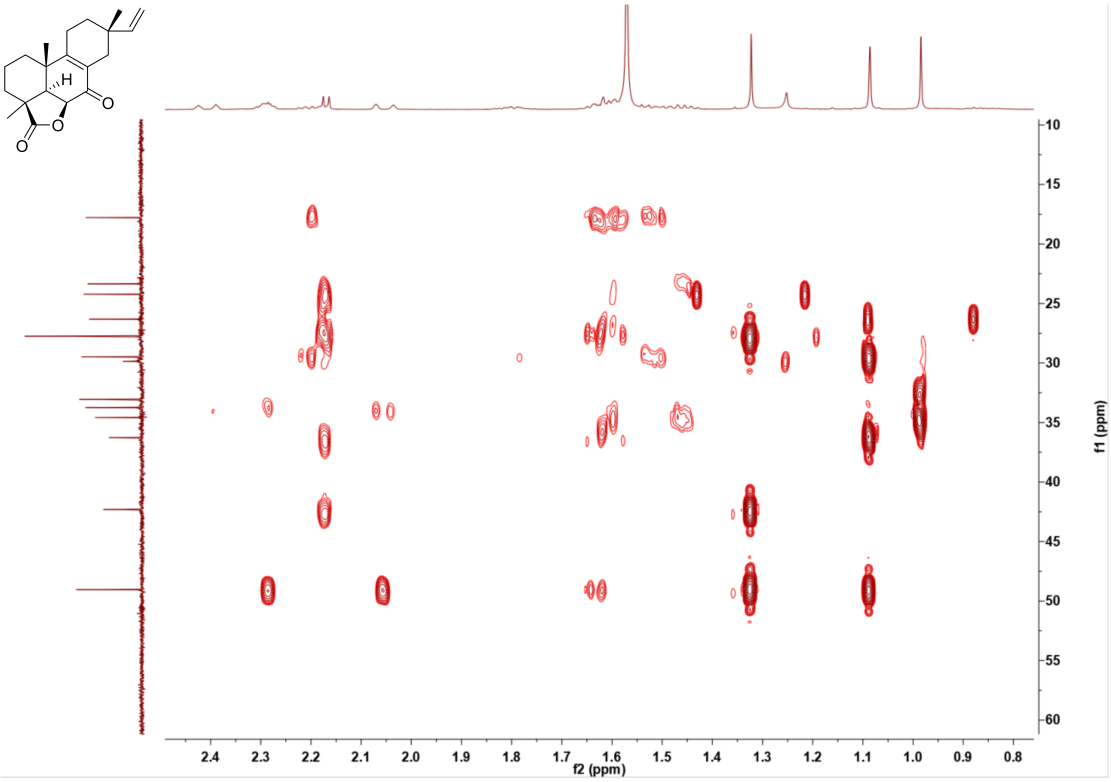

Figure S25. Enlarged HMBC (600 MHz) spectrum of **2** in $CDCl_3$.

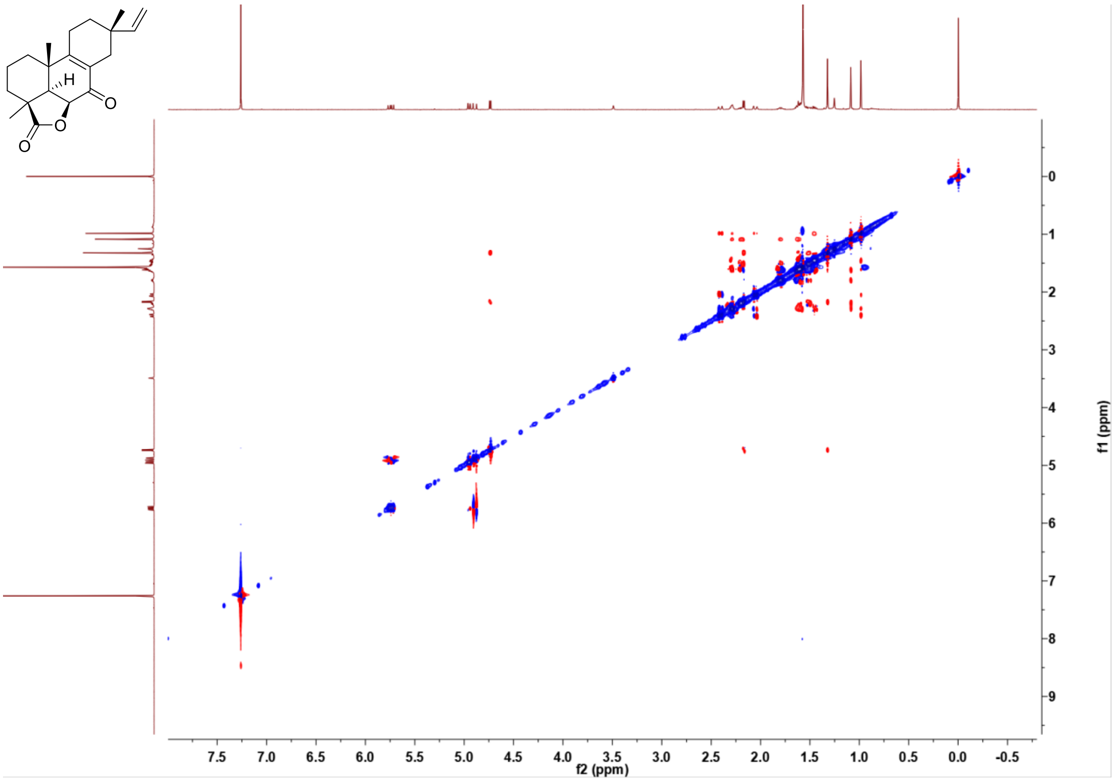

Figure S26. ROESY (600 MHz) spectrum of **2** in $CDCl_3$.

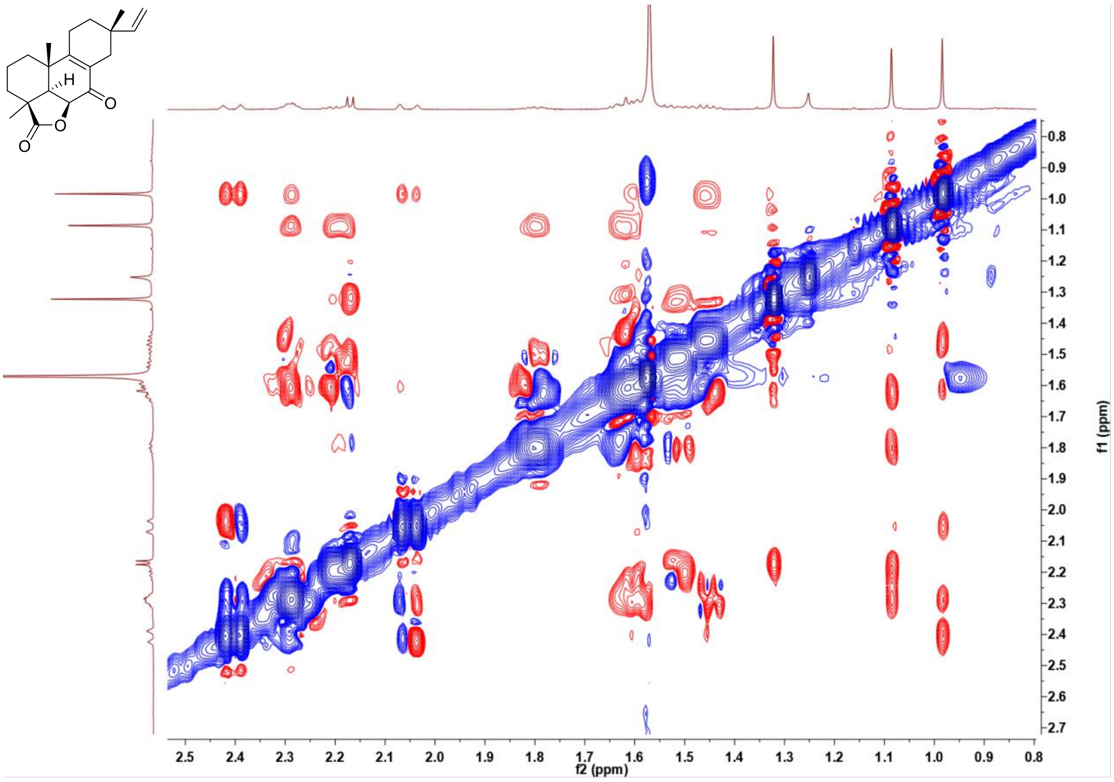

Figure S27. Enlarged ROESY (600 MHz) spectrum of **2** in $CDCl_3$.

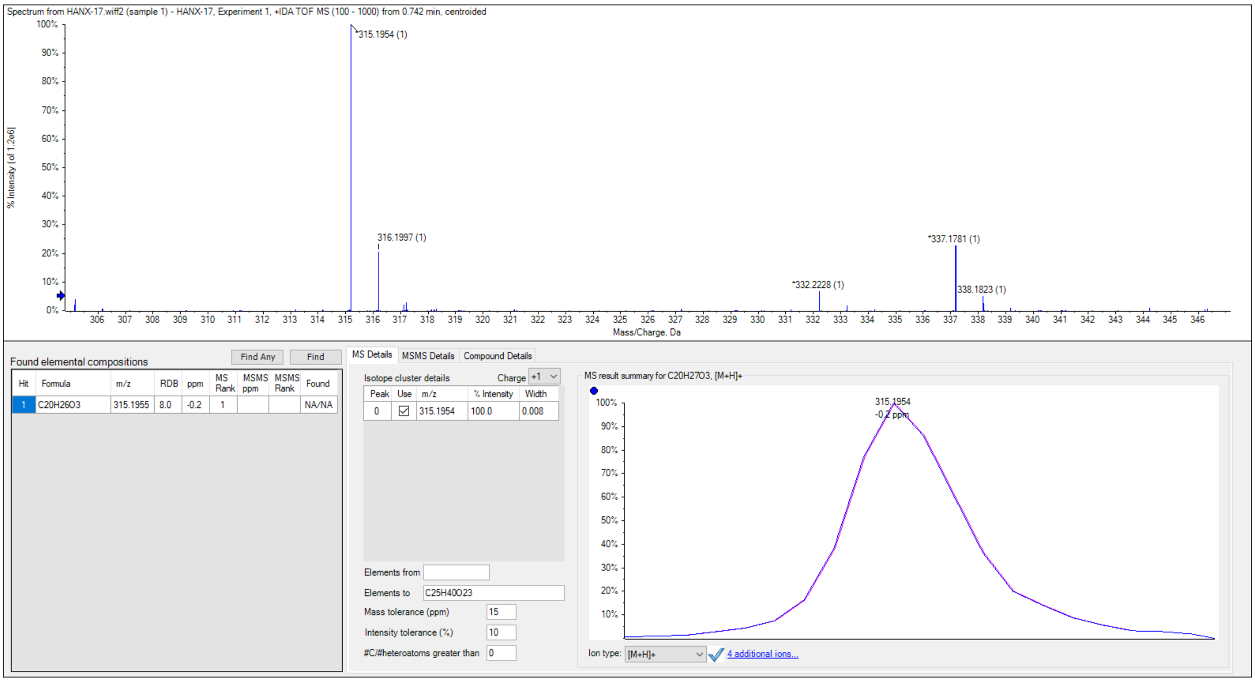

Figure S28. HRESIMS of **2**.

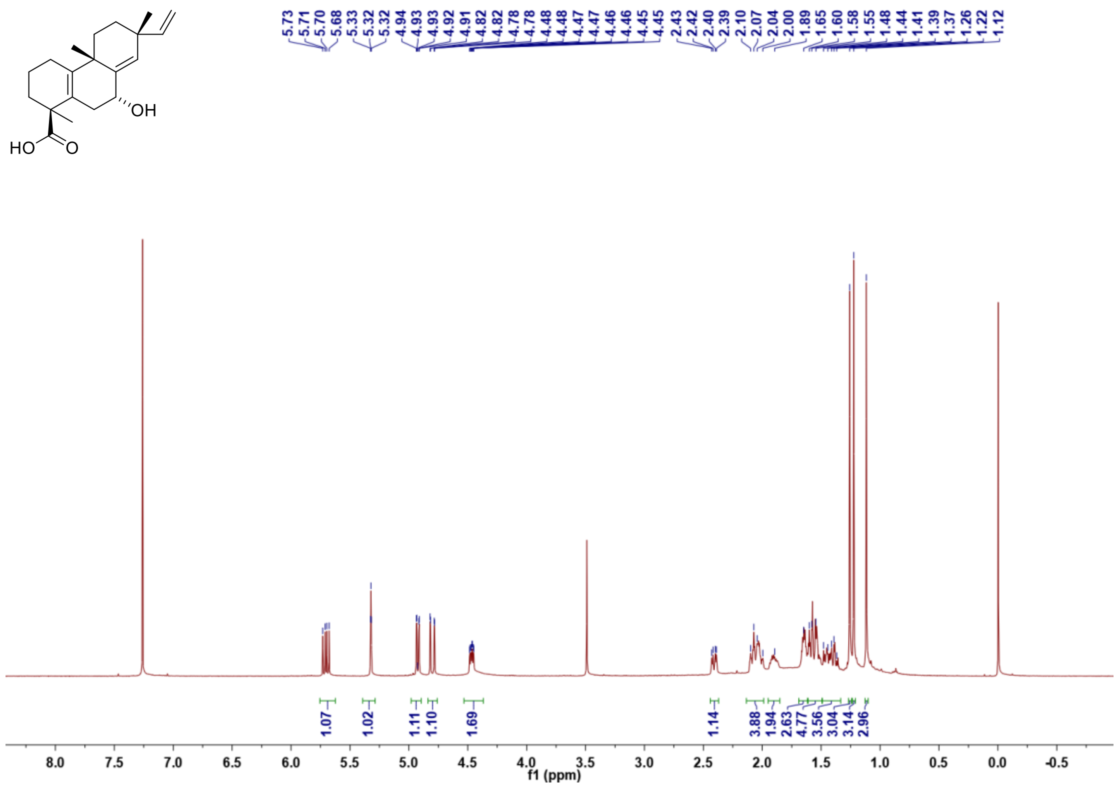

Figure S29. ^{1}H NMR (600 MHz) spectrum of **3** in $CDCl_3$.

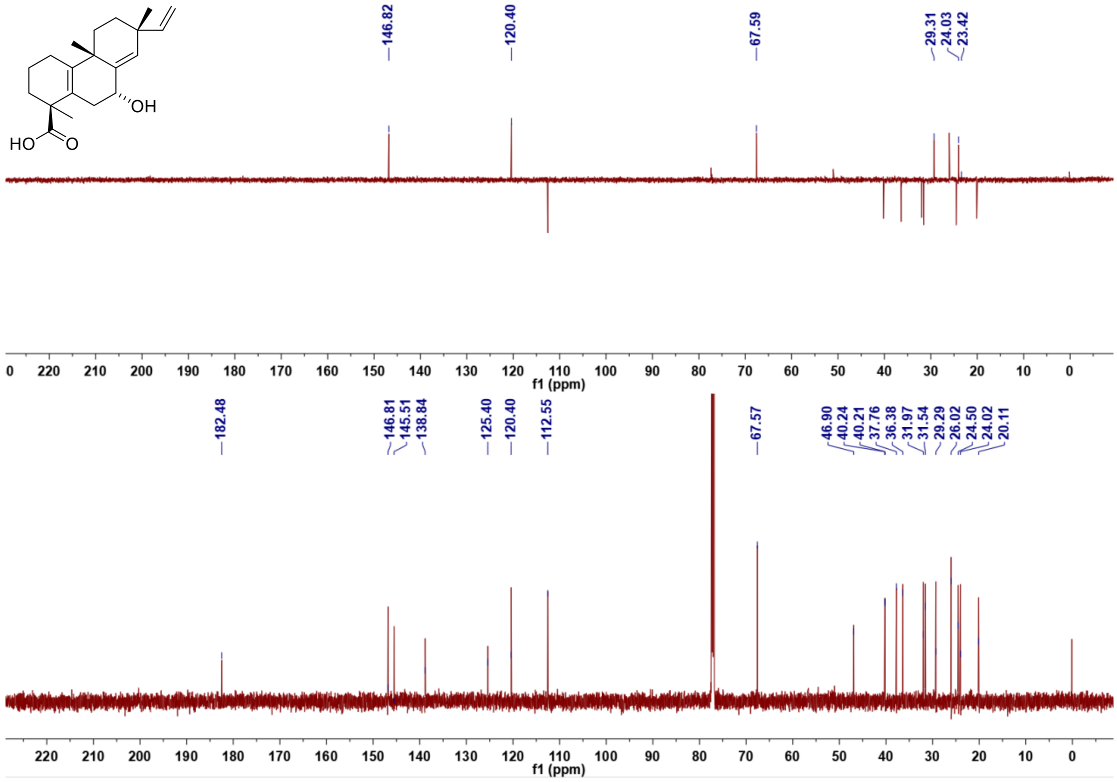

Figure S30. ^{13}C NMR and DEPT (150 MHz) spectra of **3** in $CDCl_3$.

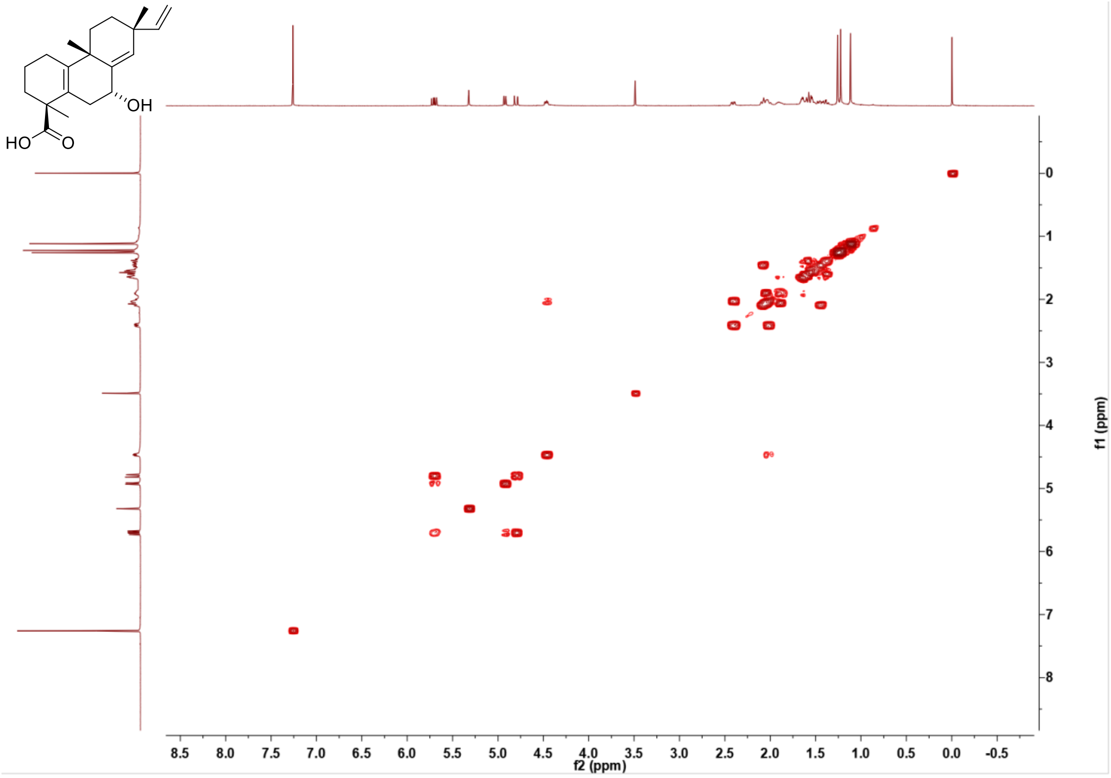

Figure S31. ^{1}H-^{1}H COSY (600 MHz) spectrum of **3** in $CDCl_3$.

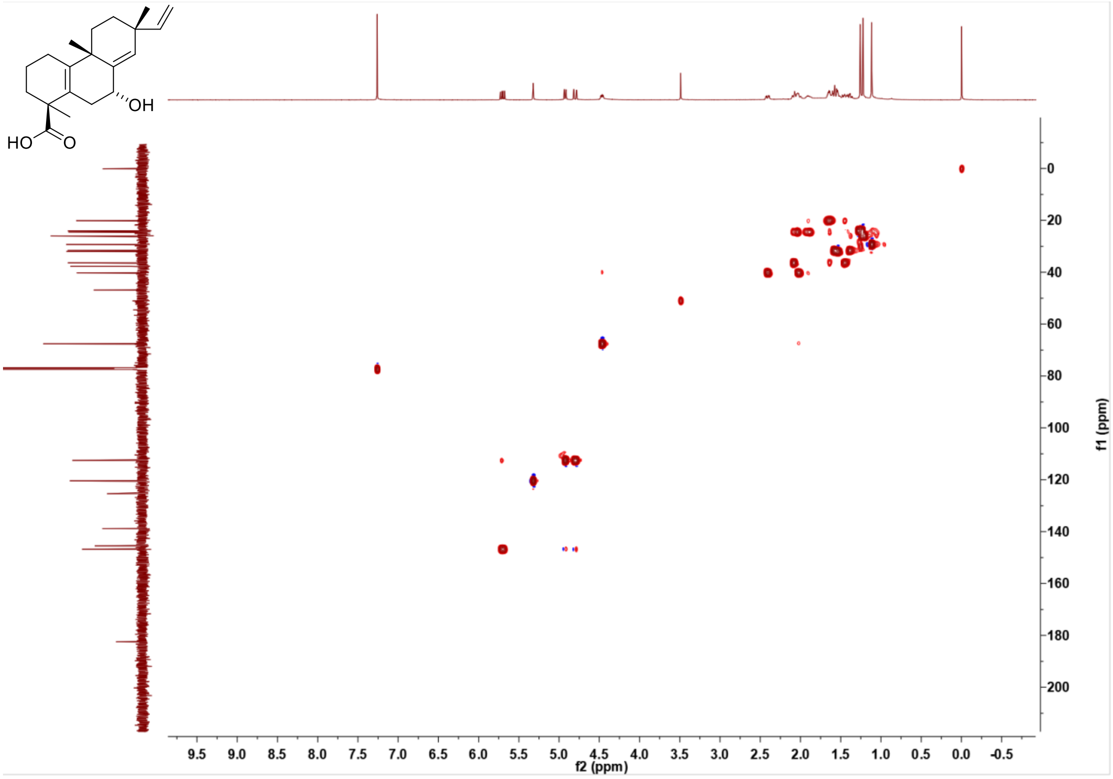

Figure S32. HSQC (600 MHz) spectrum of **3** in $CDCl_3$.

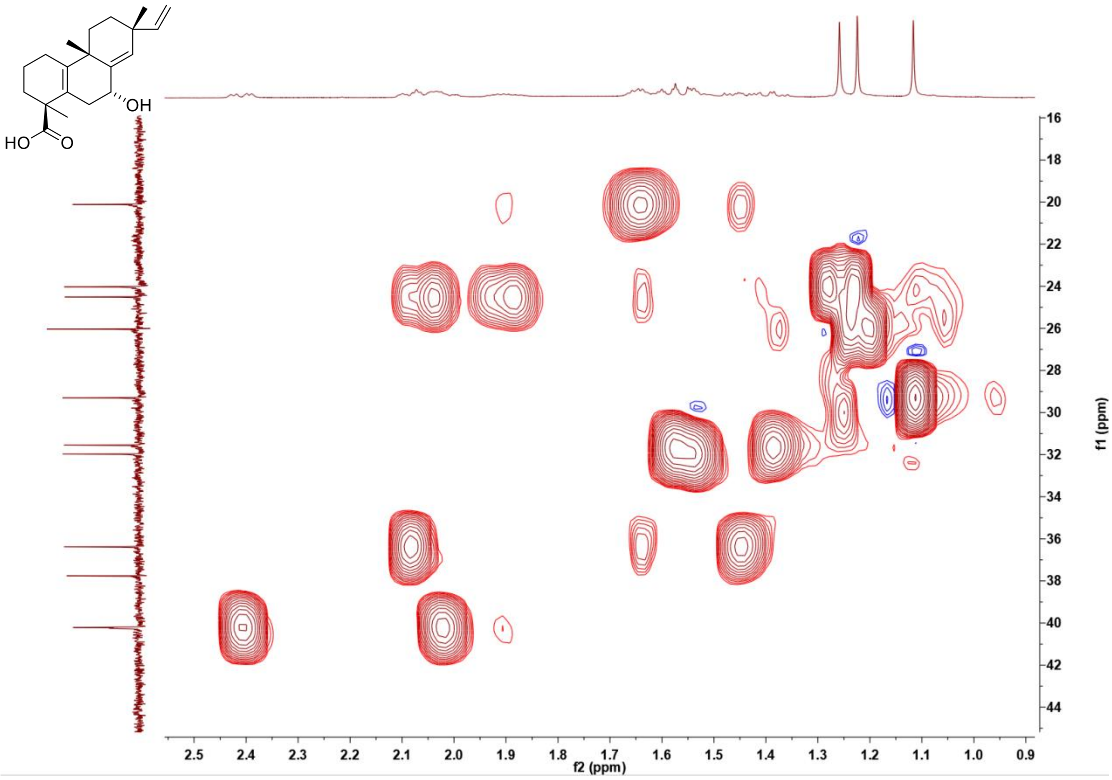

Figure S33. Enlarged HSQC (600 MHz) spectrum of **3** in $CDCl_3$.

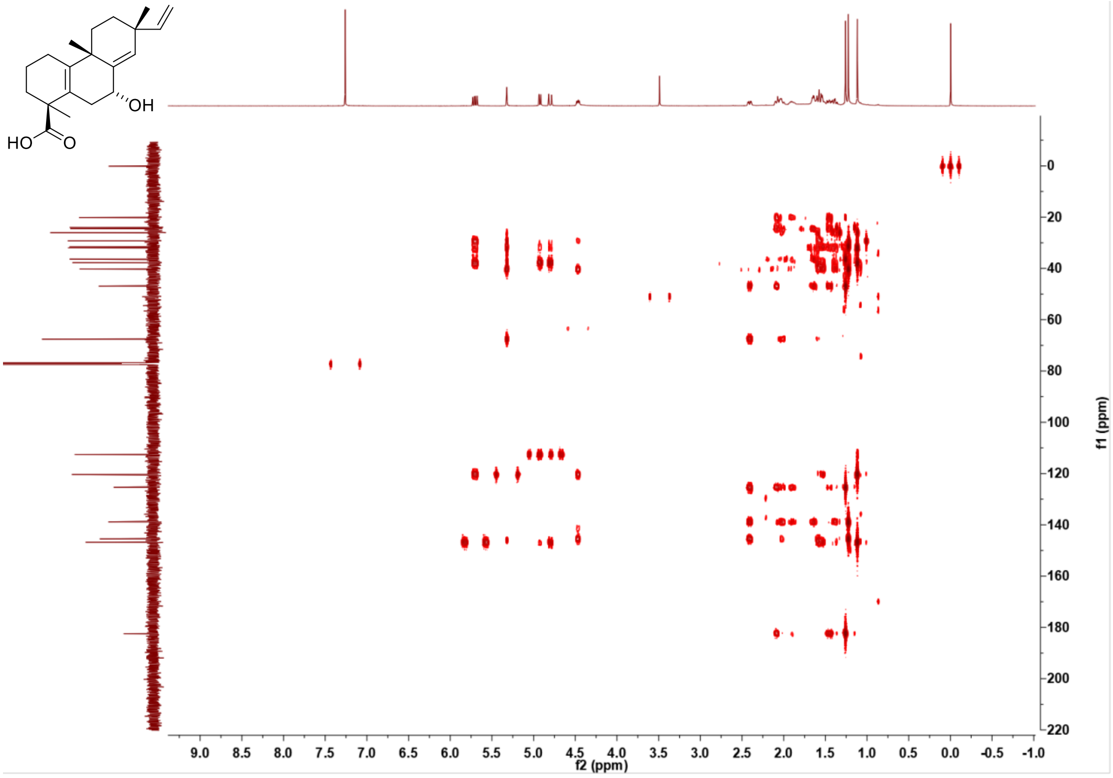

Figure S34. HMBC (600 MHz) spectrum of **3** in $CDCl_3$.

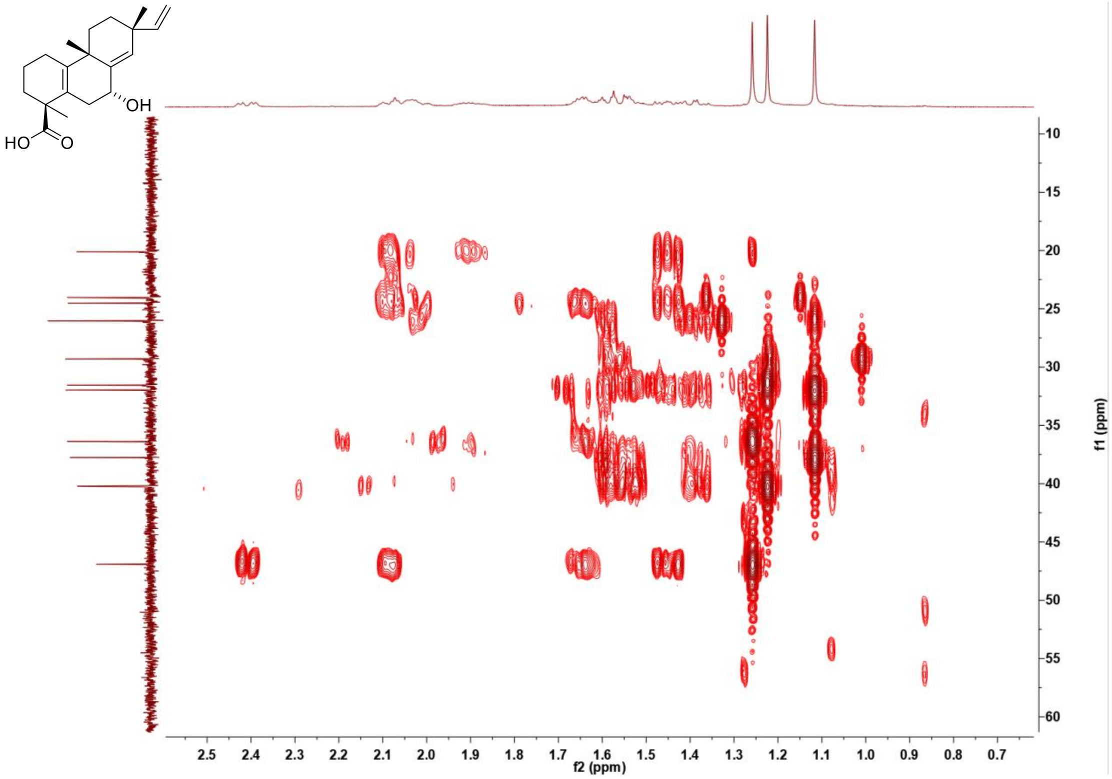

Figure S35. Enlarged HMBC (600 MHz) spectrum of **3** in $CDCl_3$.

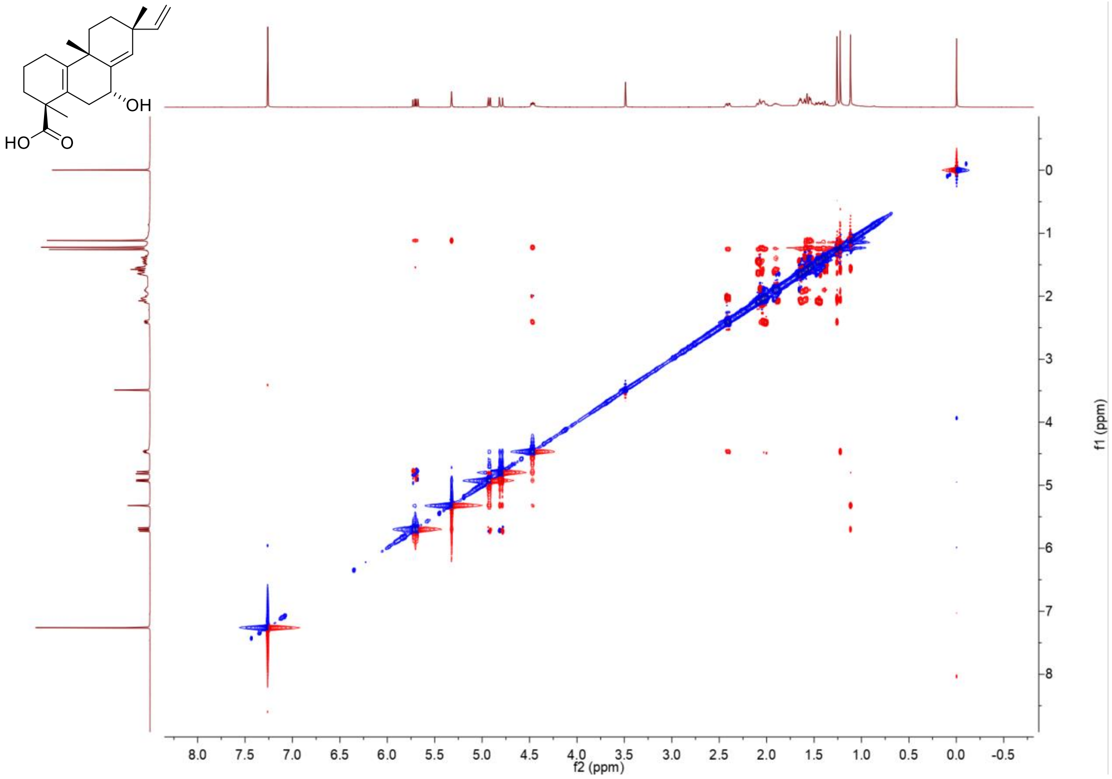

Figure S36. ROESY (600 MHz) spectrum of **3** in $CDCl_3$.

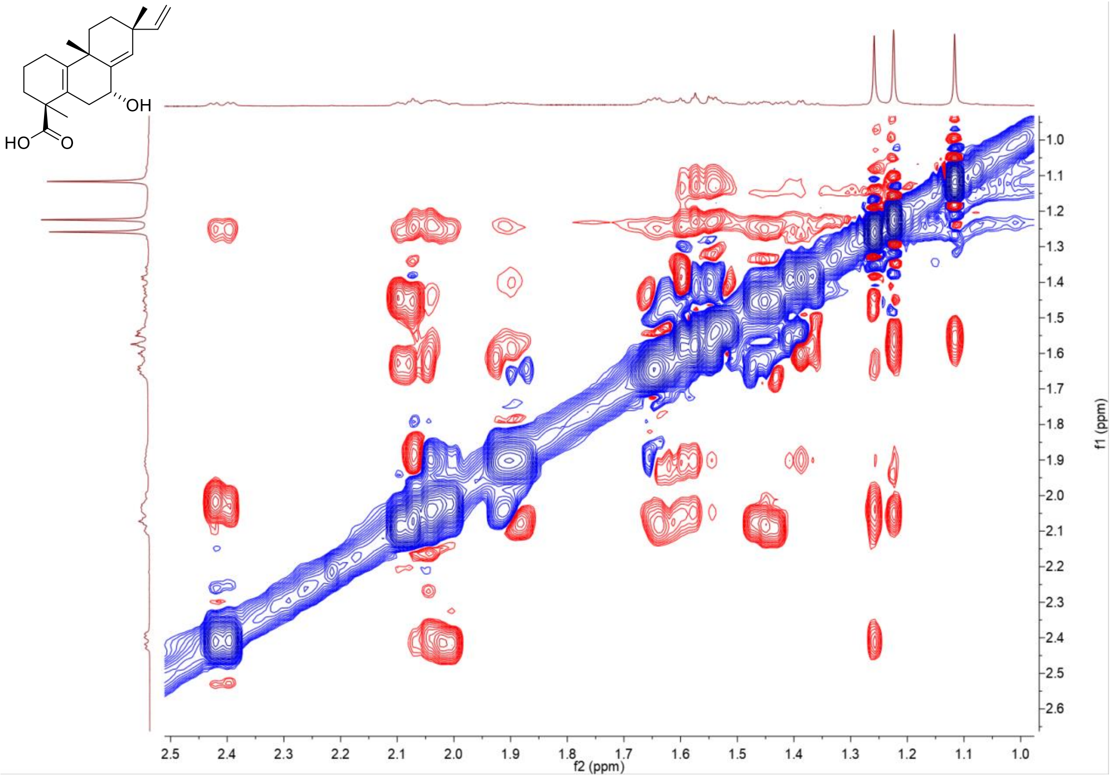

Figure S37. Enlarged ROESY (600 MHz) spectrum of **3** in $CDCl_3$.

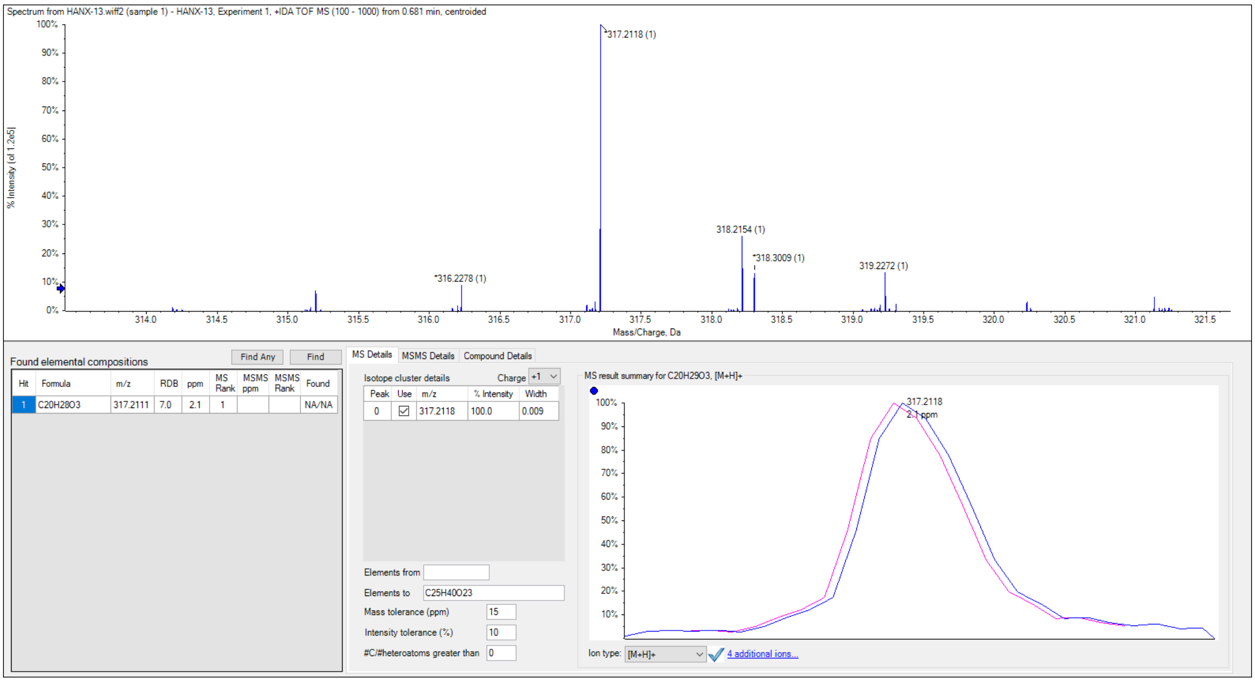

Figure S38. HRESIMS of **3**.

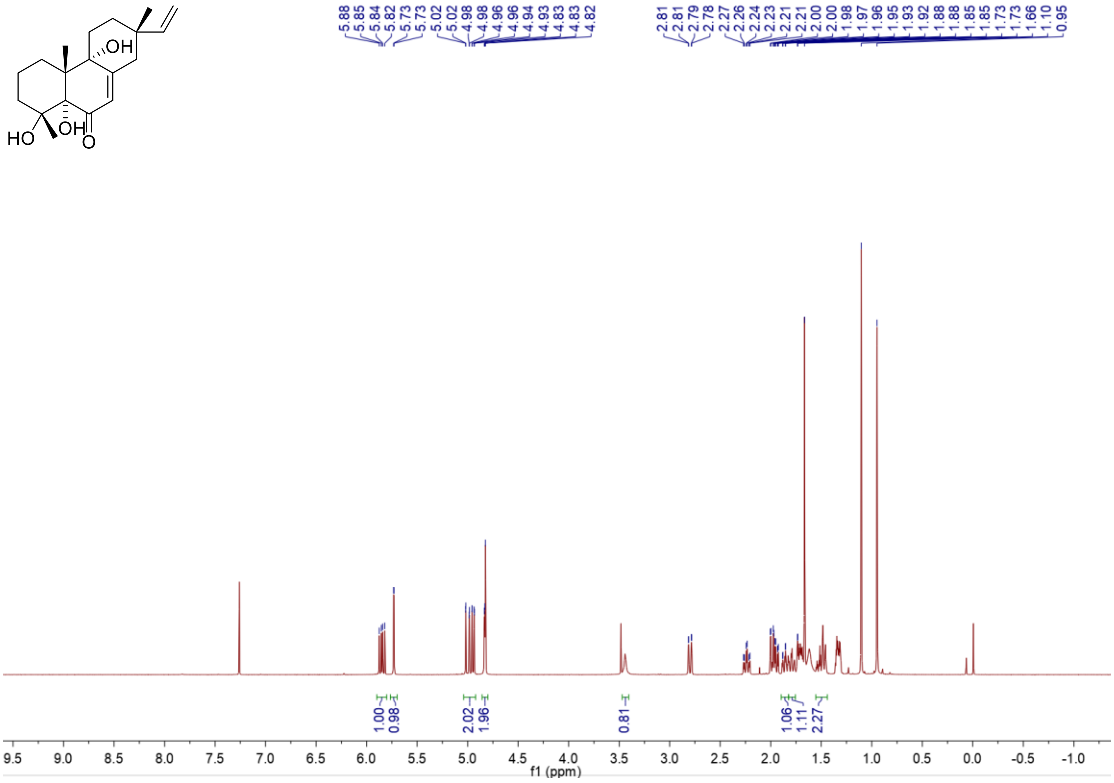

Figure S39. ^{1}H NMR (600 MHz) spectrum of **4** in $CDCl_3$.

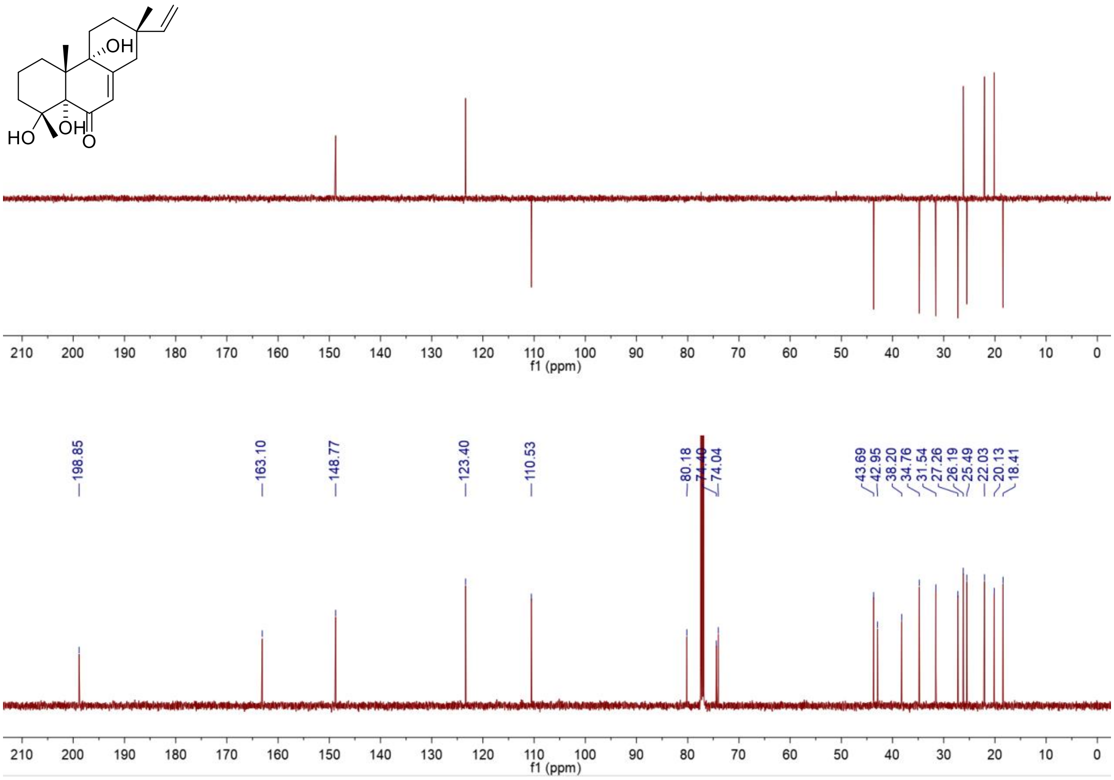

Figure S40. ^{13}C NMR and DEPT (150 MHz) spectra of **4** in $CDCl_3$.

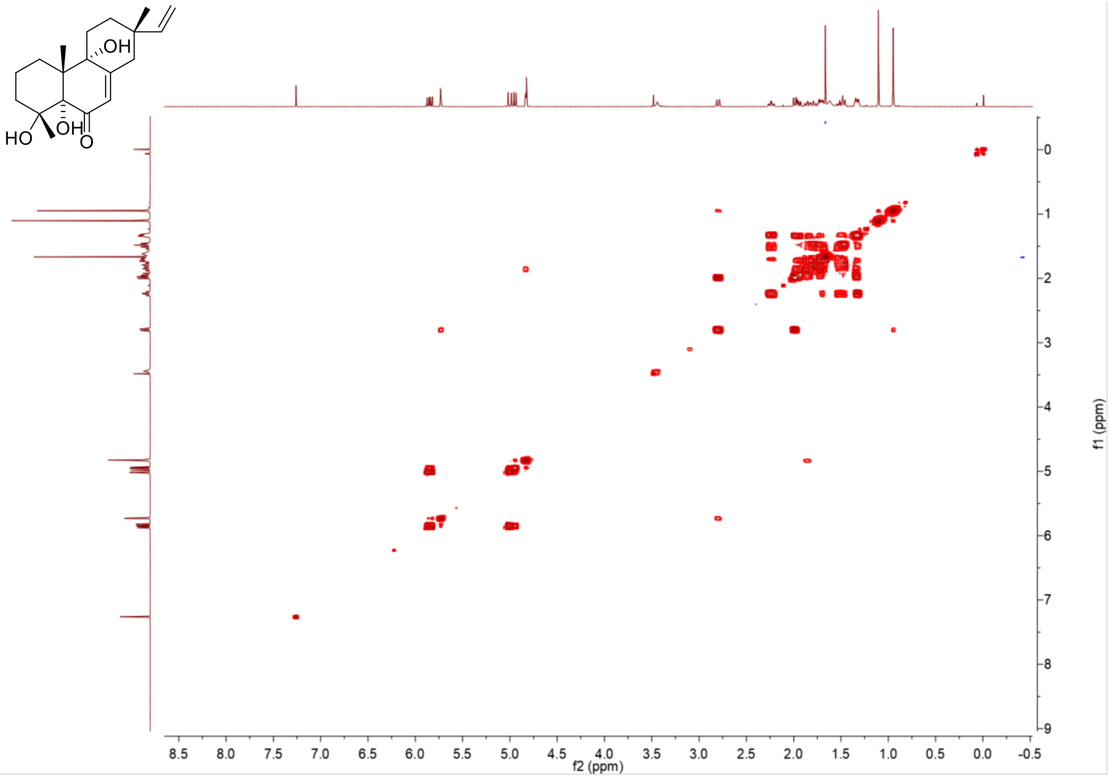

Figure S41. ^{1}H-^{1}H COSY (600 MHz) spectrum of **4** in $CDCl_3$.

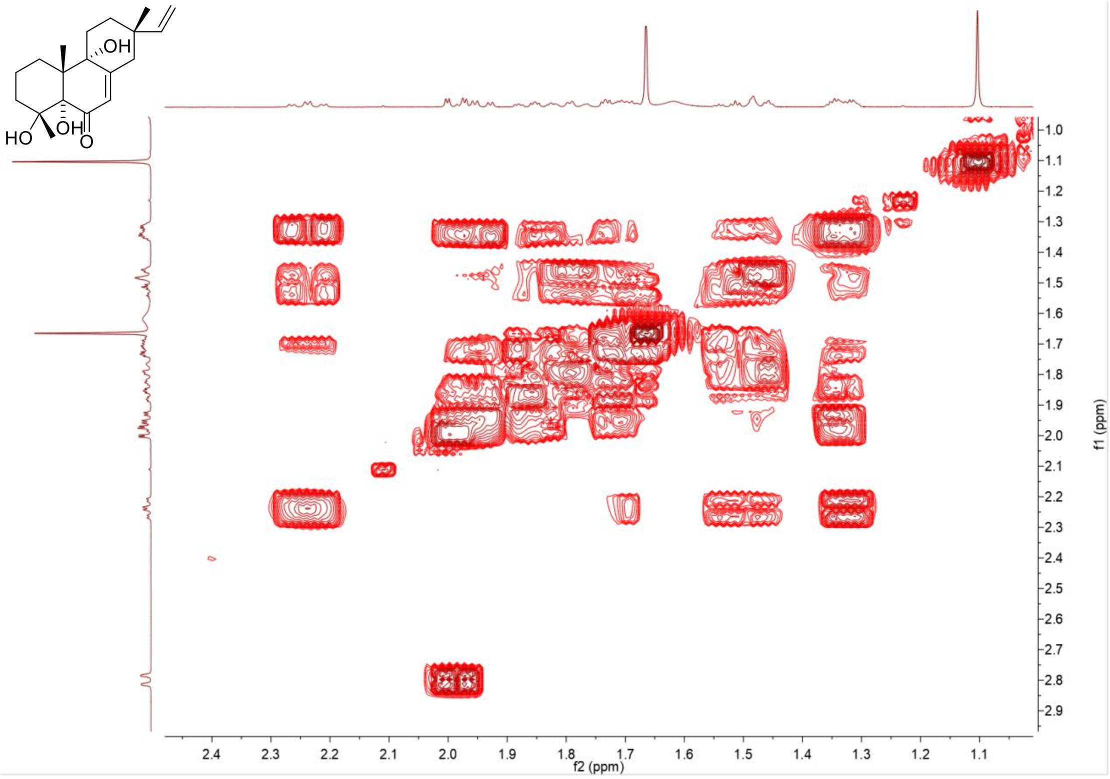

Figure S42. Enlarged ^{1}H-^{1}H COSY (600 MHz) spectrum of **4** in $CDCl_3$.

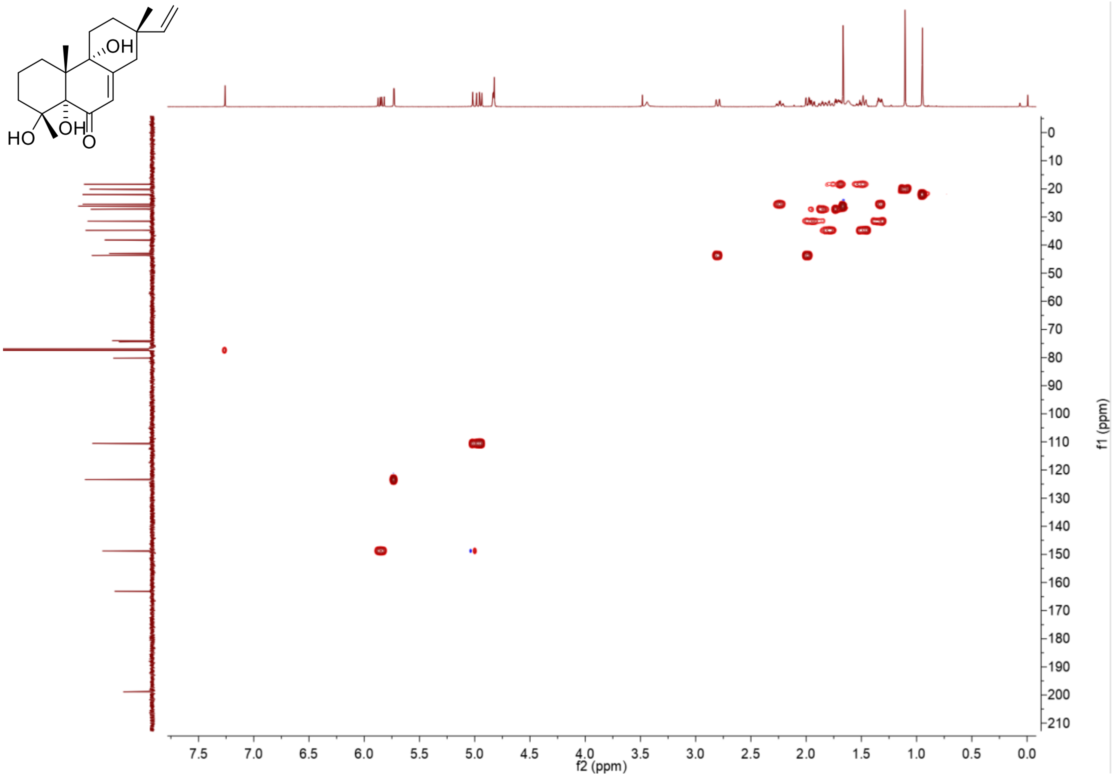

Figure S43. HSQC (600 MHz) spectrum of **4** in $CDCl_3$.

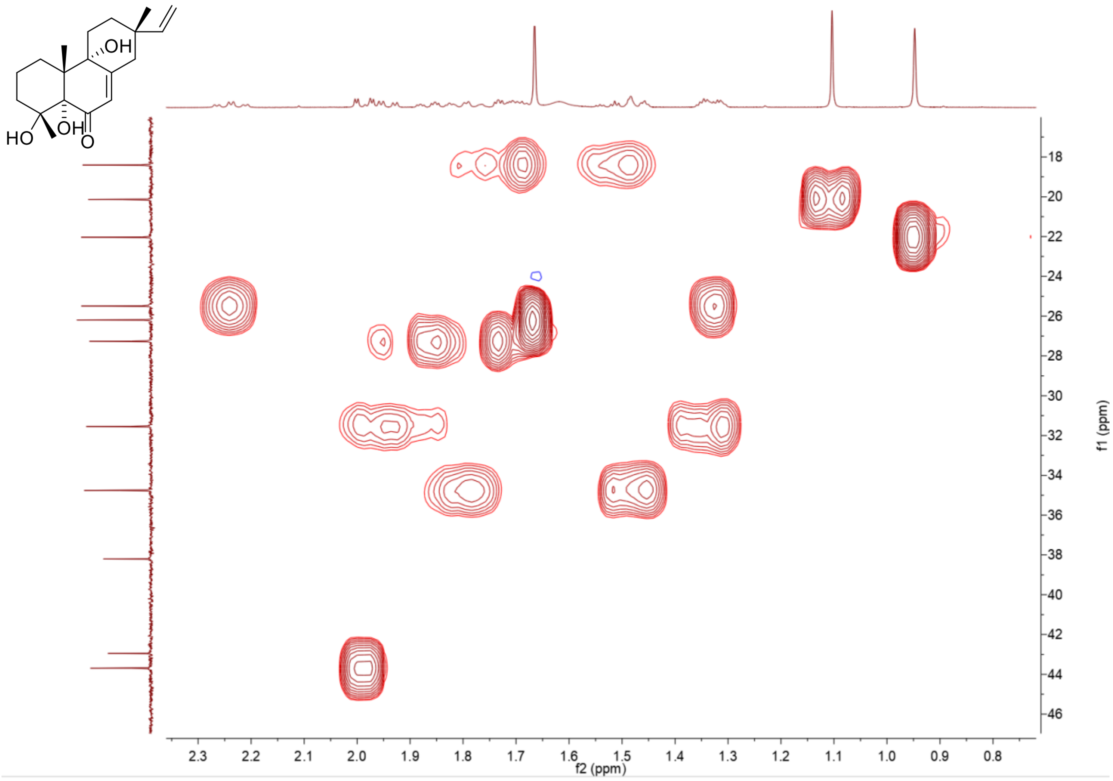

Figure S44. Enlarged HSQC (600 MHz) spectrum of **4** in $CDCl_3$.

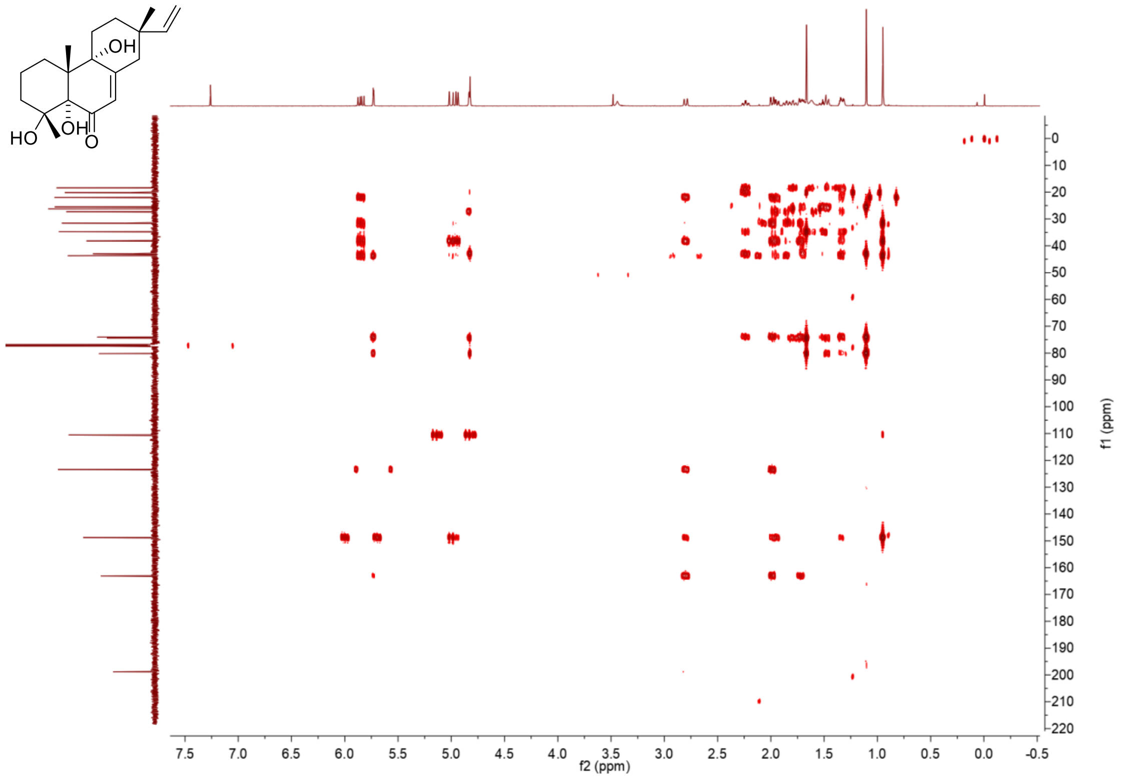

Figure S45. HMBC (600 MHz) spectrum of **4** in $CDCl_3$.

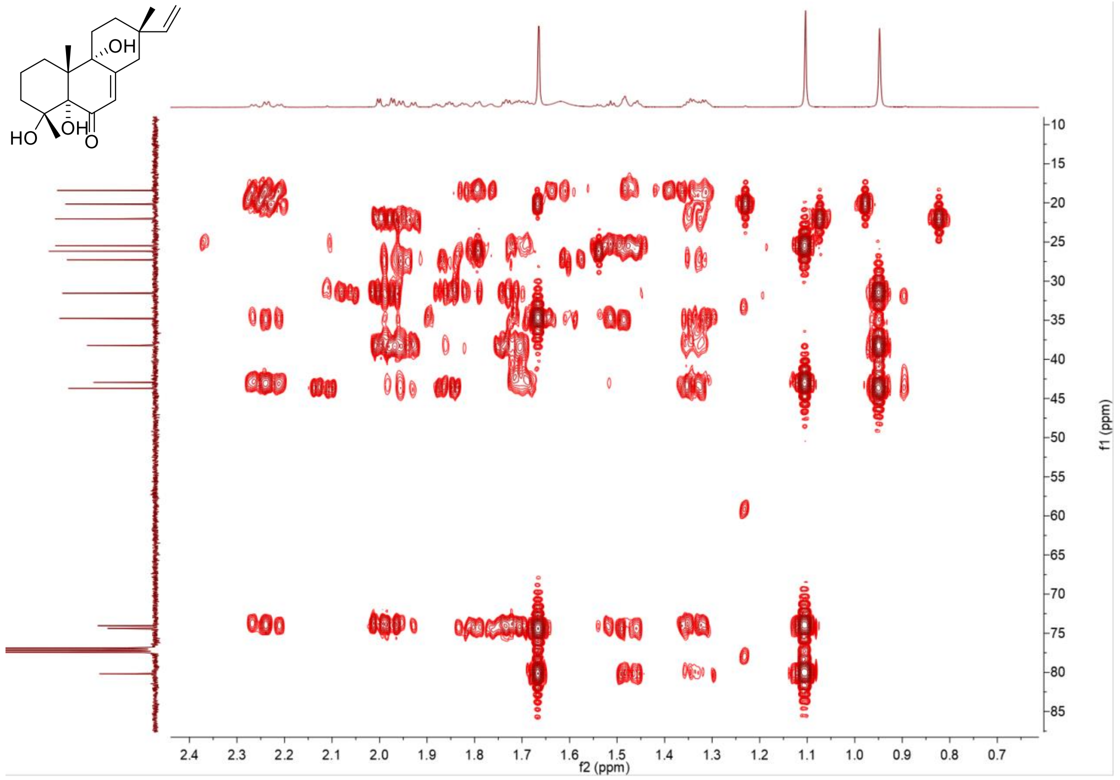

Figure S46. Enlarged HMBC (600 MHz) spectrum of **4** in $CDCl_3$.

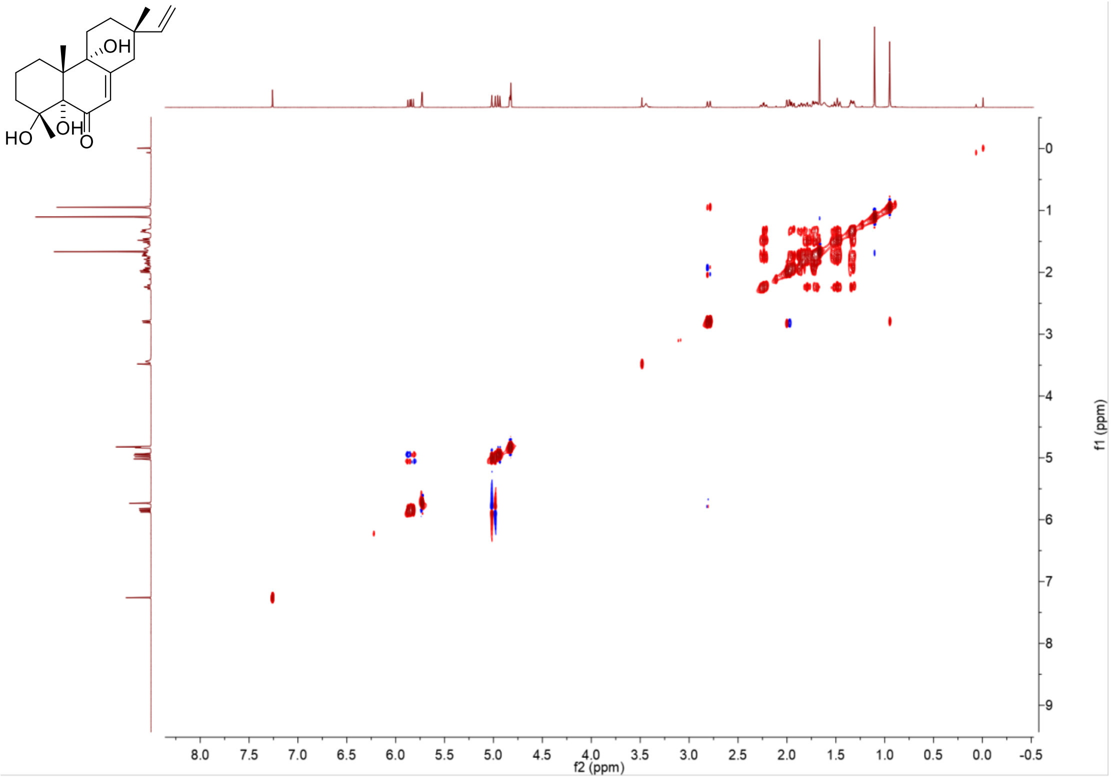

Figure S47. ROESY (600 MHz) spectrum of **4** in $CDCl_3$.

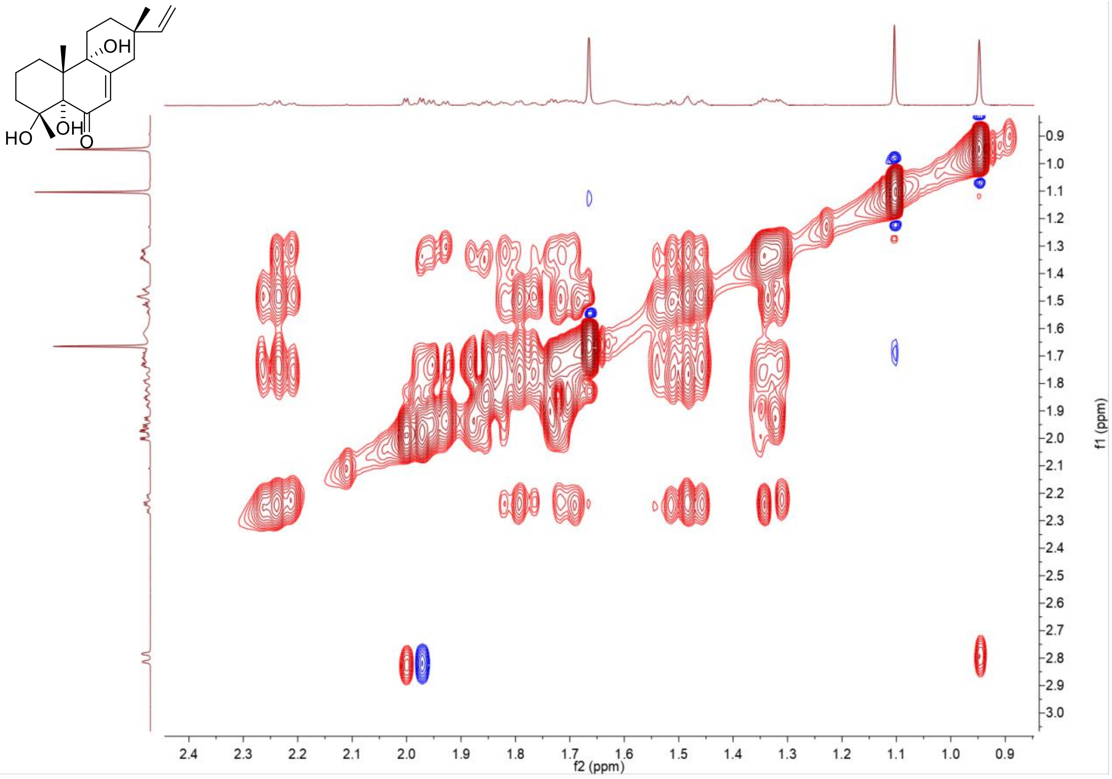

Figure S48. Enlarged ROESY (600 MHz) spectrum of **4** in $CDCl_3$.

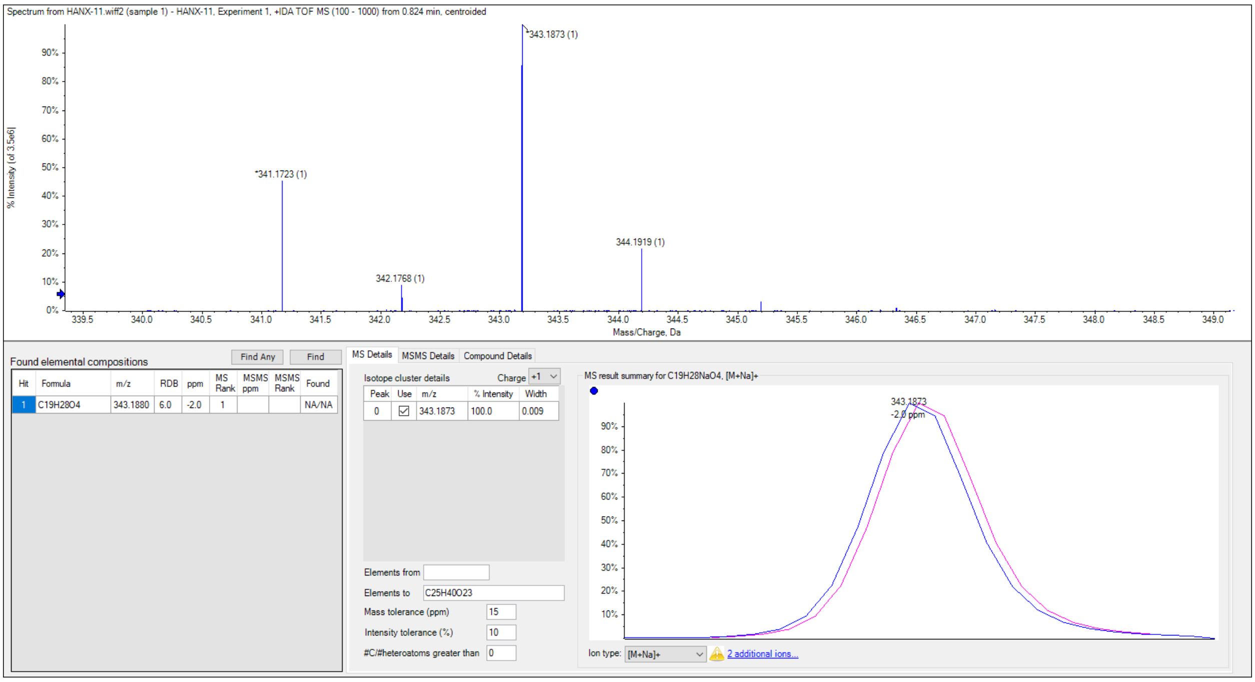

Figure S49. HRESIMS of **4**.

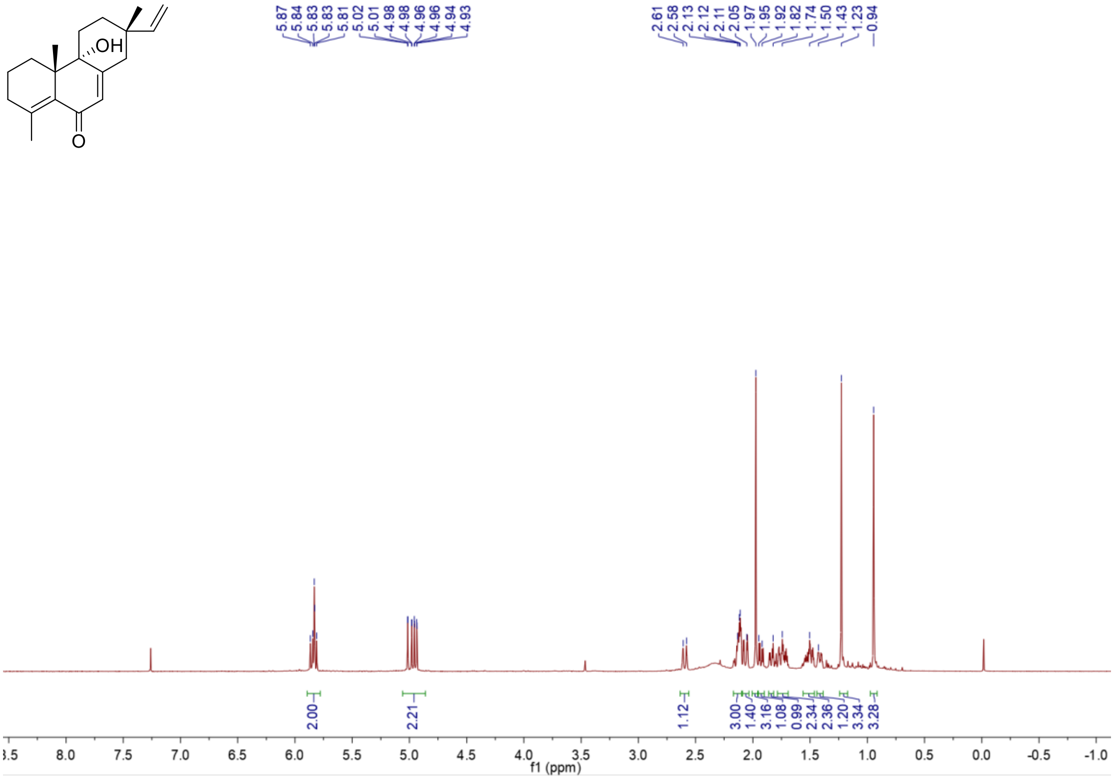

Figure S50. ¹H NMR (600 MHz) spectrum of **5** in $CDCl_3$.

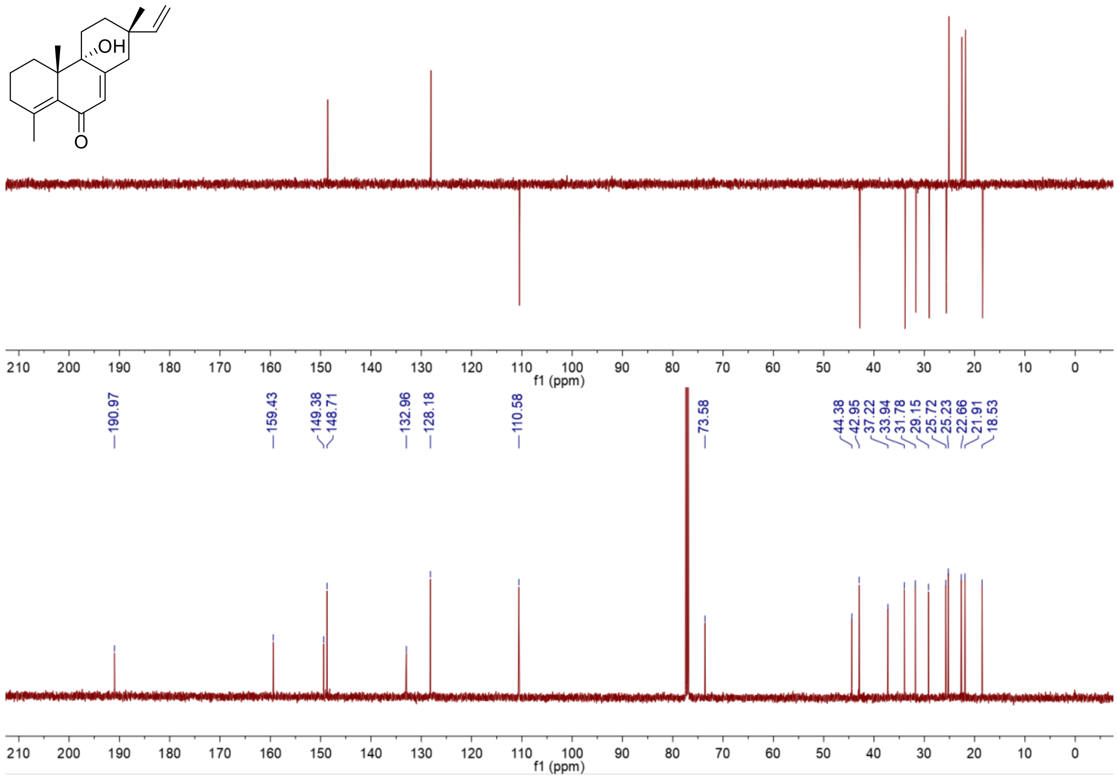

Figure S51. ^{13}C NMR and DEPT (150 MHz) spectra of **5** in $CDCl_3$.

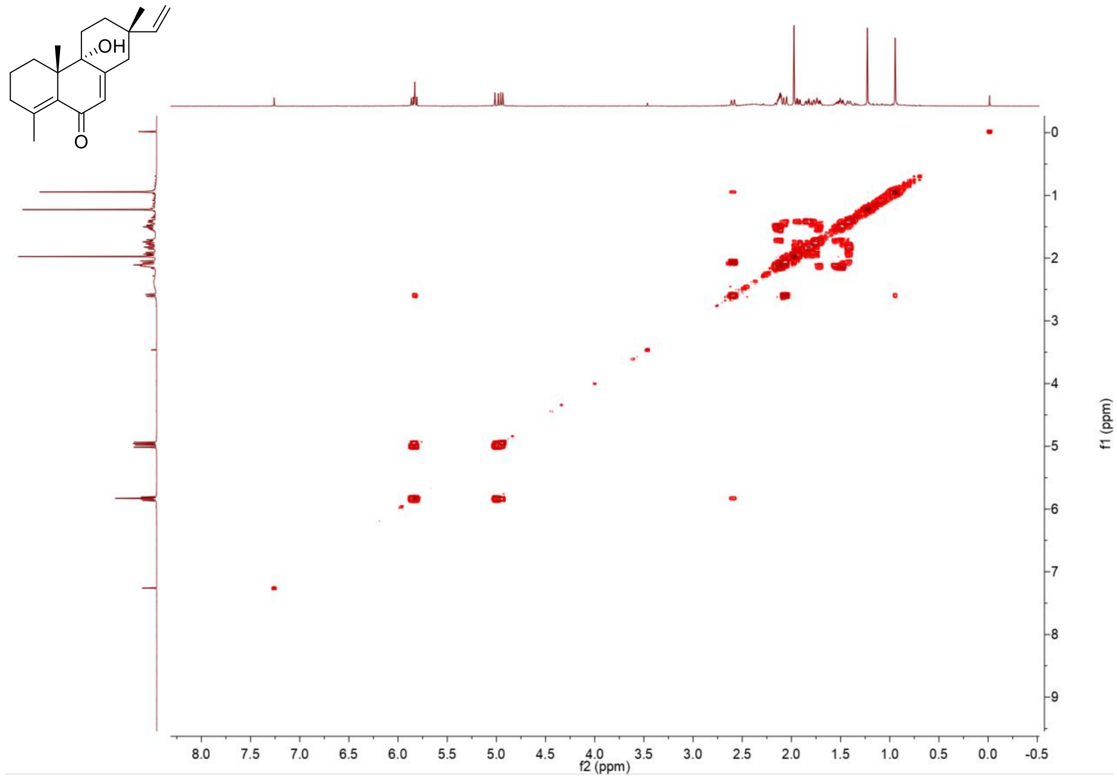

Figure S52. ^{1}H-^{1}H COSY (600 MHz) spectrum of **5** in $CDCl_3$.

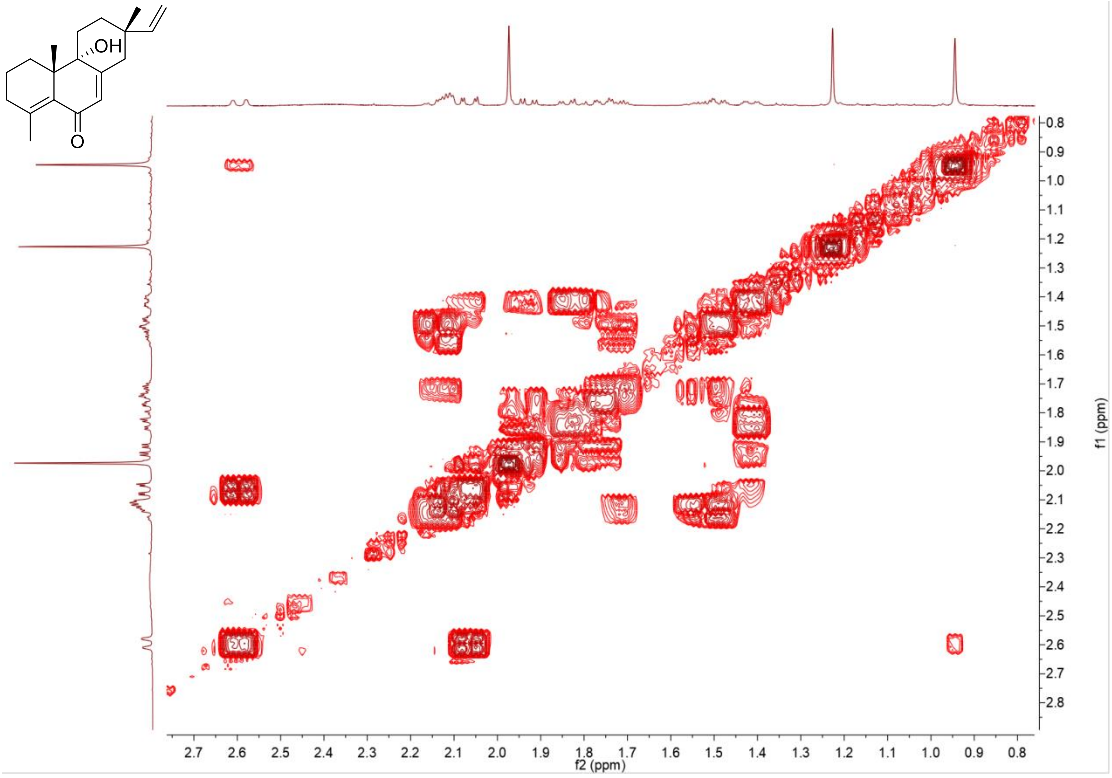

Figure S53. Enlarged ^{1}H-^{1}H COSY (600 MHz) spectrum of **5** in $CDCl_3$.

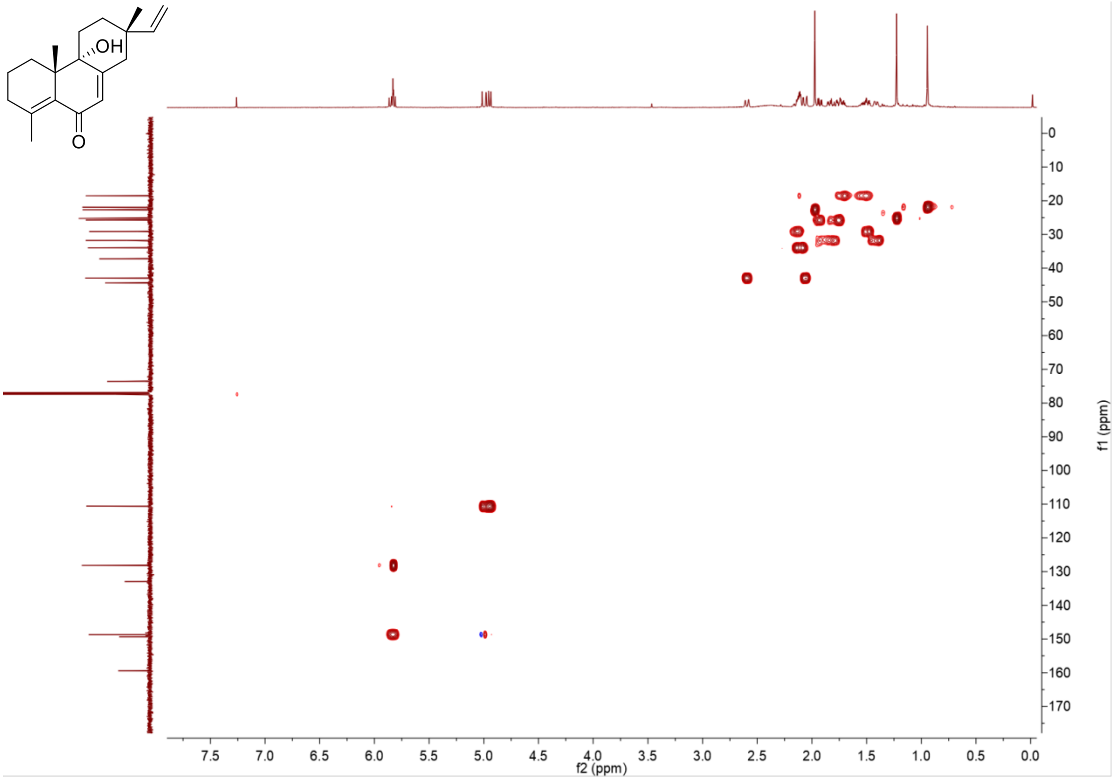

Figure S54. HSQC (600 MHz) spectrum of **5** in $CDCl_3$.

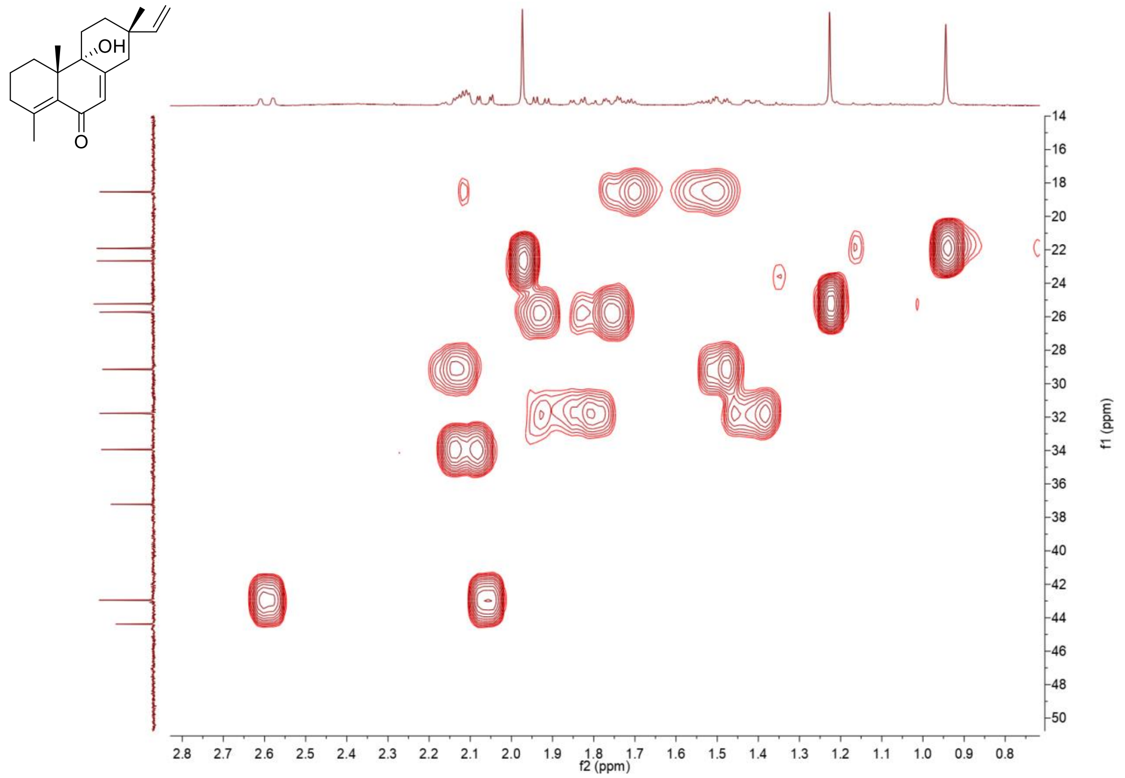

Figure S55. Enlarged HSQC (600 MHz) spectrum of **5** in $CDCl_3$.

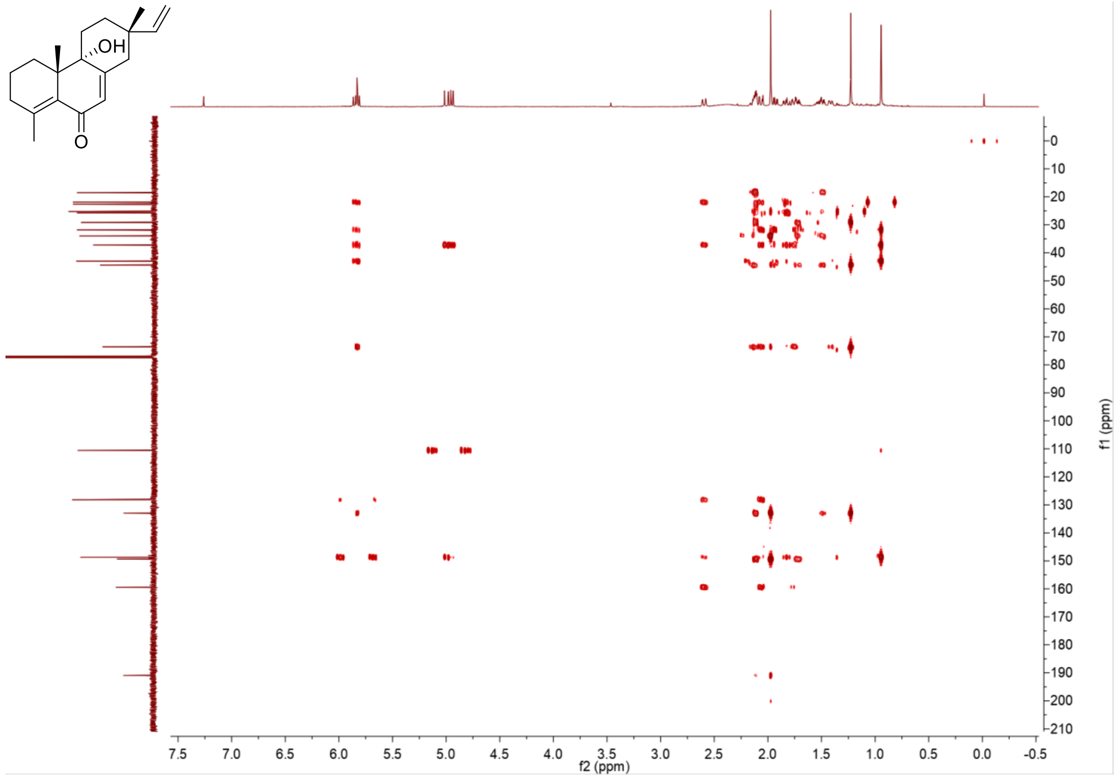

Figure S56. HMBC (600 MHz) spectrum of **5** in $CDCl_3$.

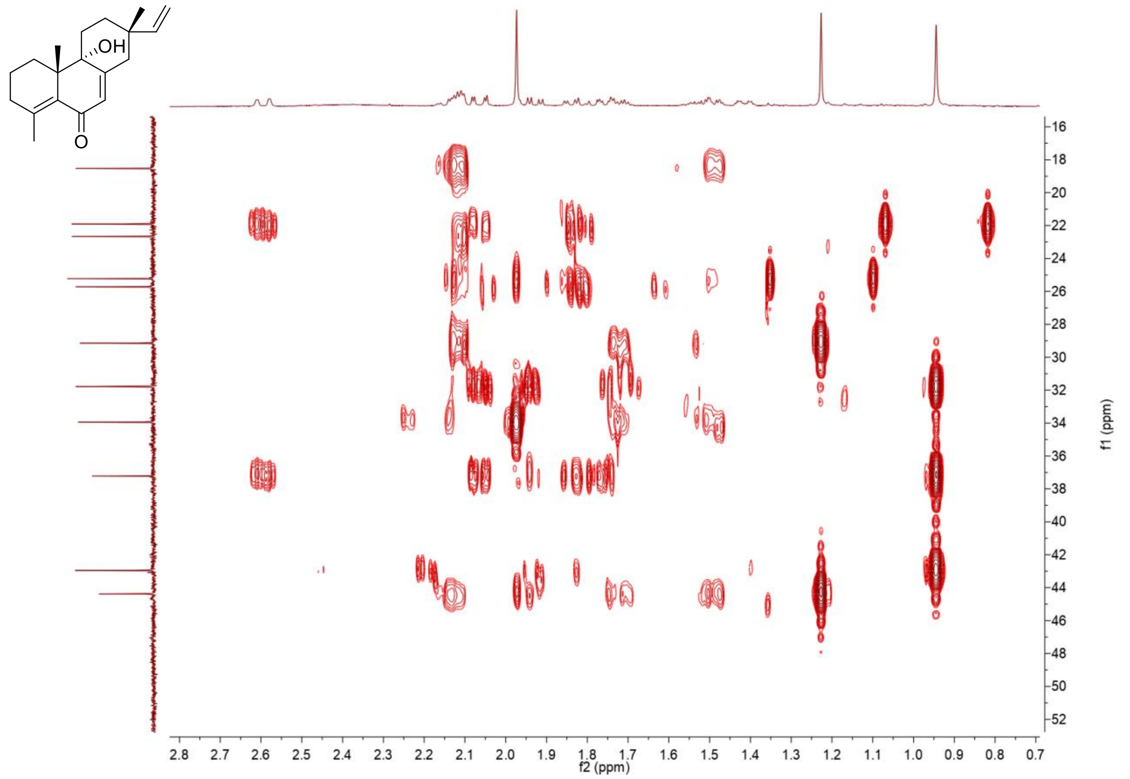

Figure S57. Enlarged HMBC (600 MHz) spectrum of **5** in $CDCl_3$.

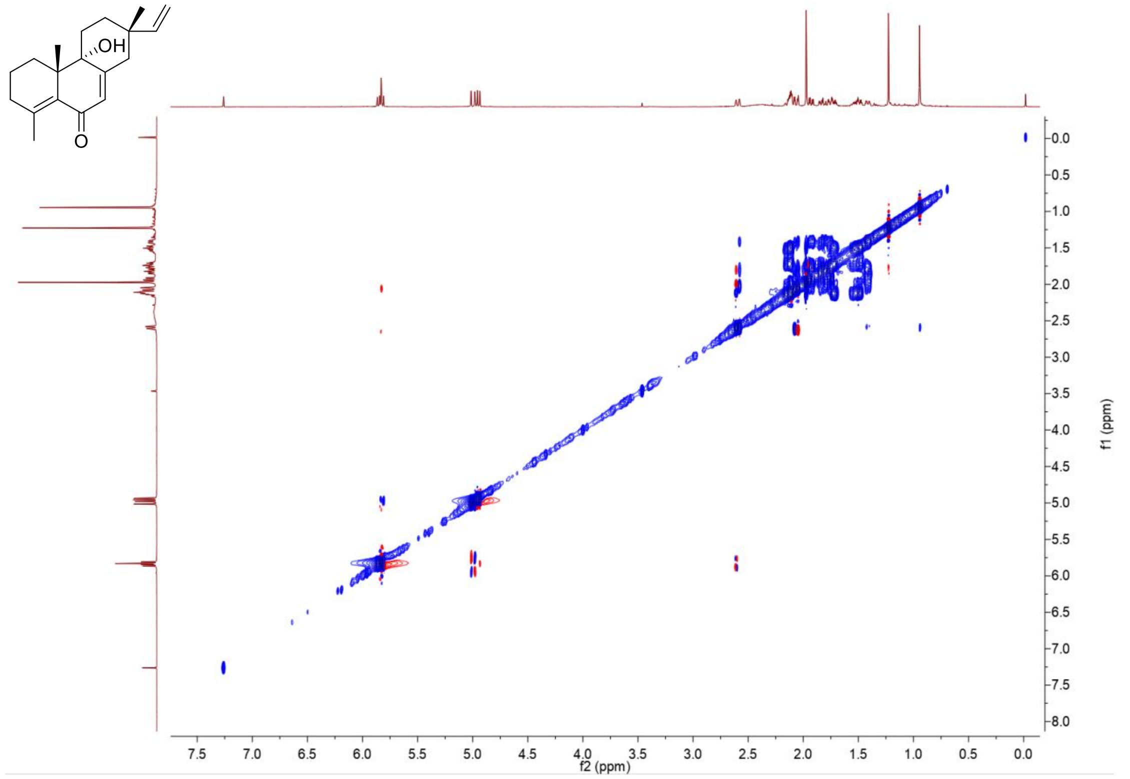

Figure S58. ROESY (600 MHz) spectrum of **5** in $CDCl_3$.

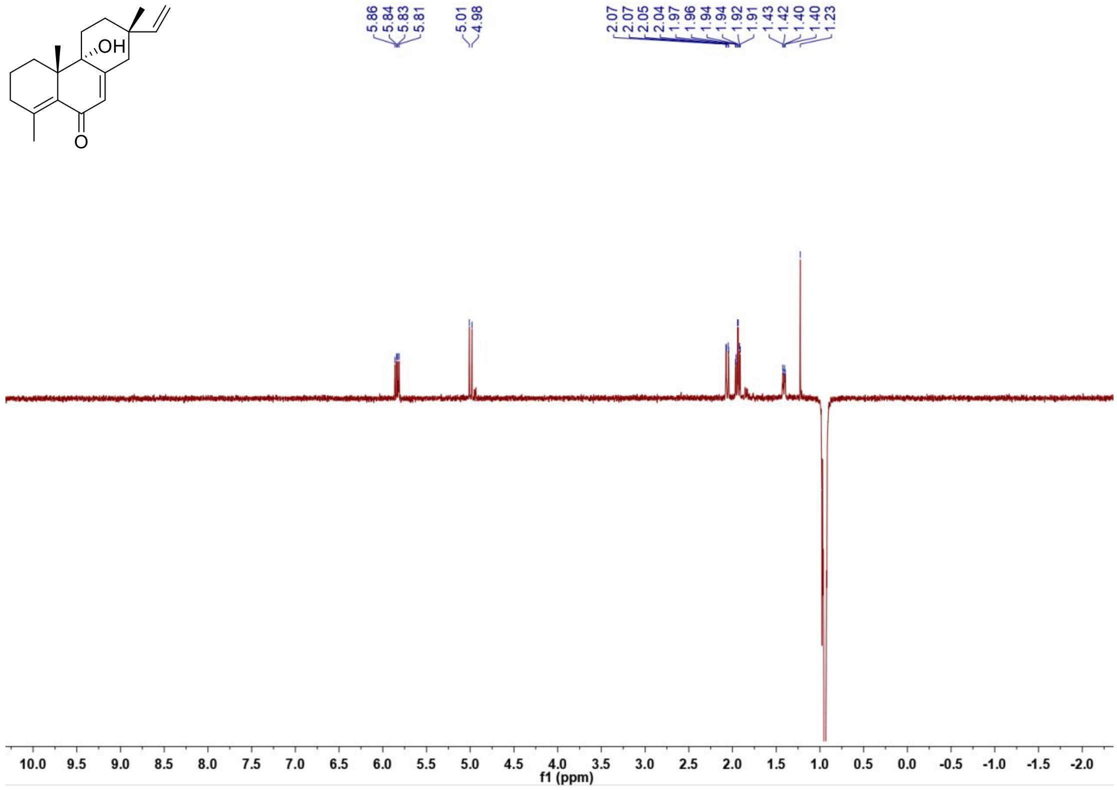

Figure S59. NOE irradiation spectrum (freq: 0.94 ppm) of compound **5** in $CDCl_3$.

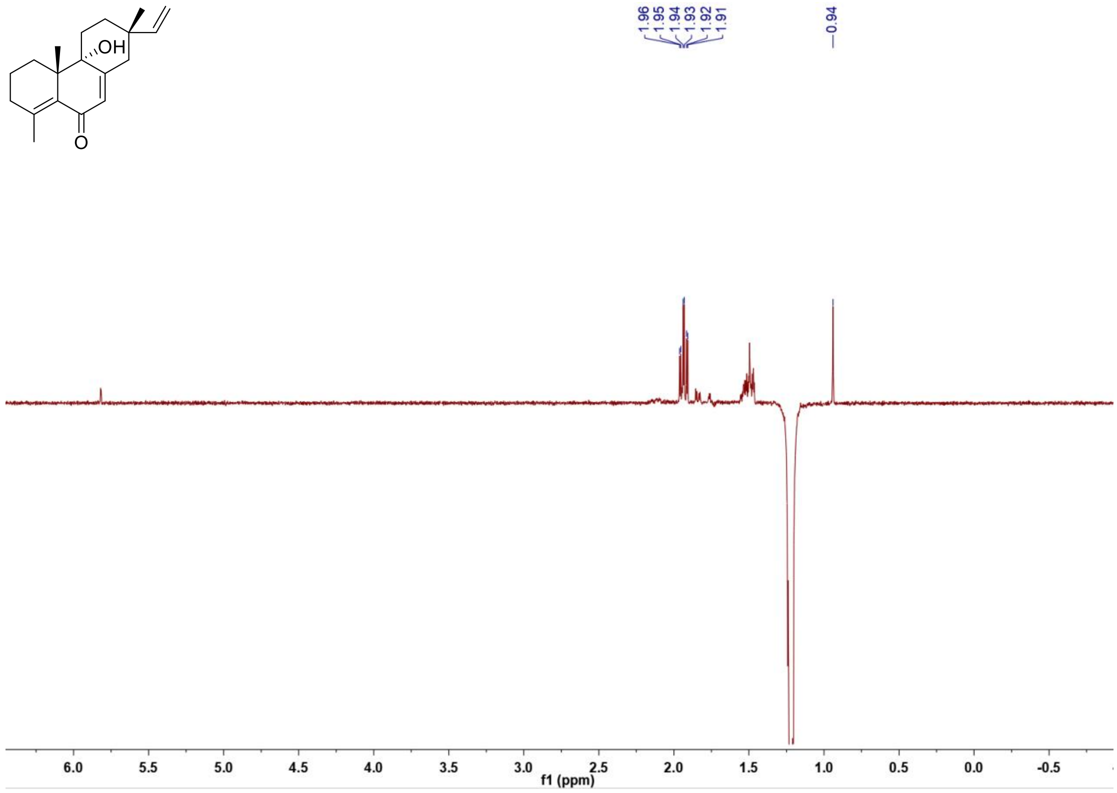

Figure S60. NOE irradiation spectrum (freq: 1.23 ppm) of compound **5** in $CDCl_3$.

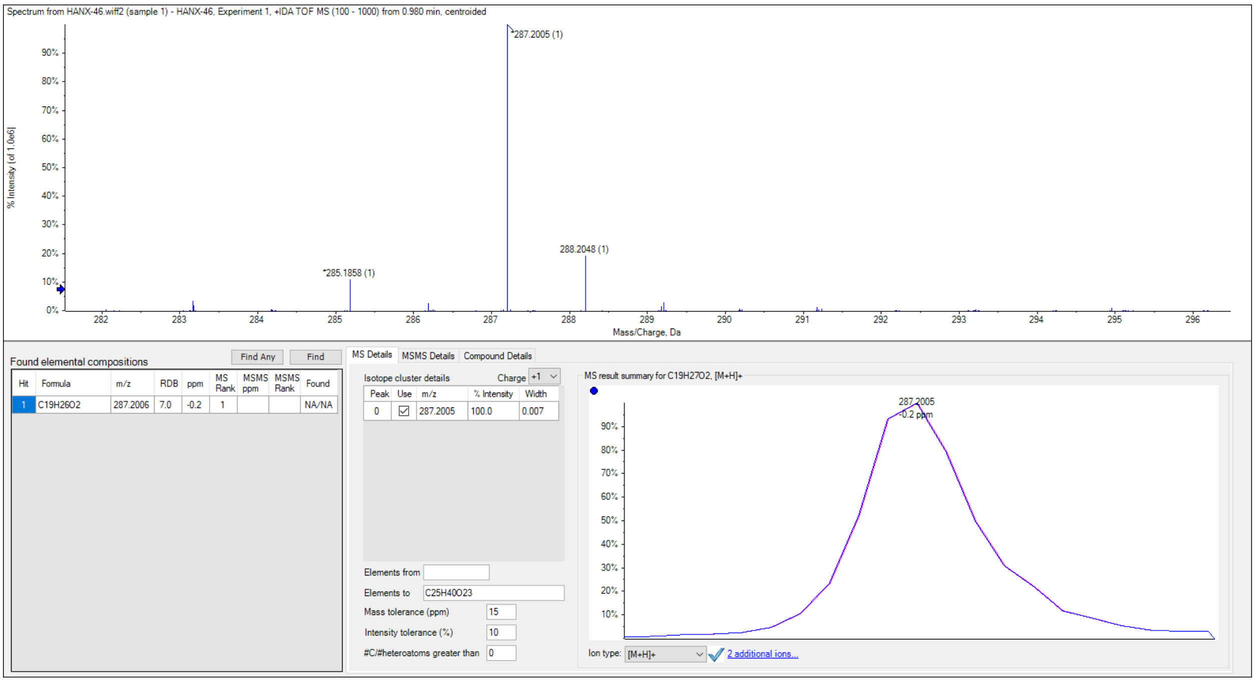

Figure S61. HRESIMS of **5**.

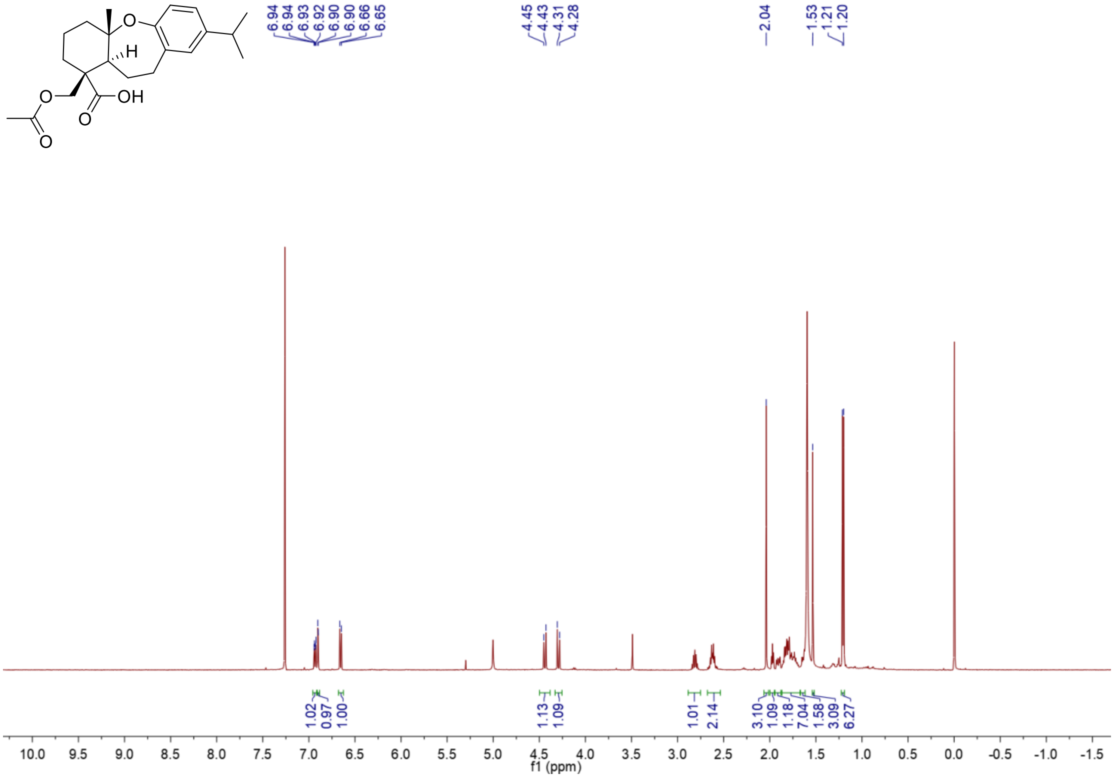

Figure S62. ^{1}H NMR (600 MHz) spectrum of **6** in $CDCl_3$.

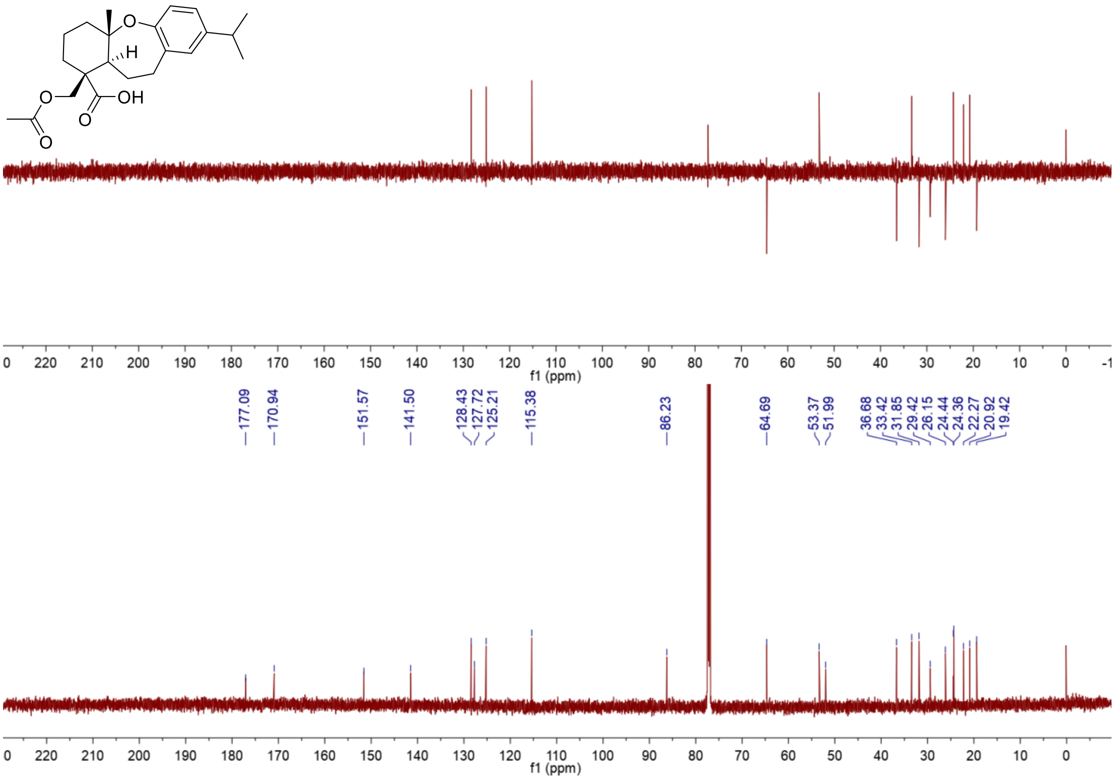

Figure S63. ^{13}C NMR and DEPT (150 MHz) spectra of **6** in $CDCl_3$.

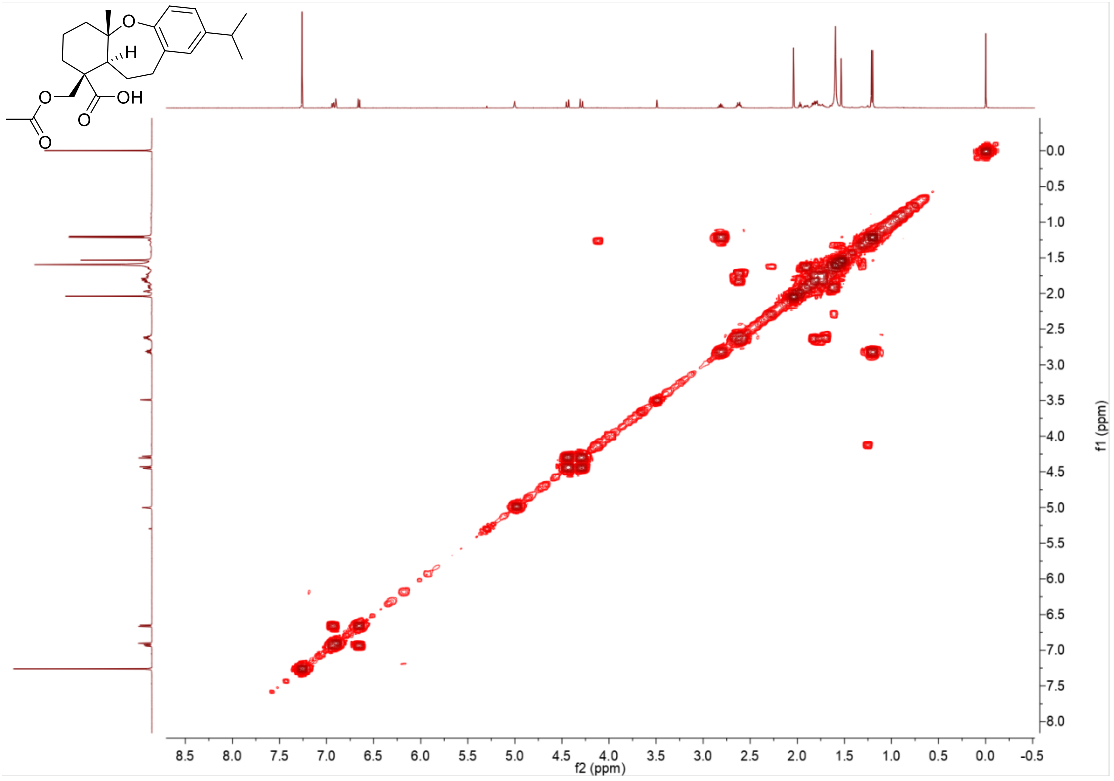

Figure S64. ^{1}H-^{1}H COSY (600 MHz) spectrum of **6** in $CDCl_3$.

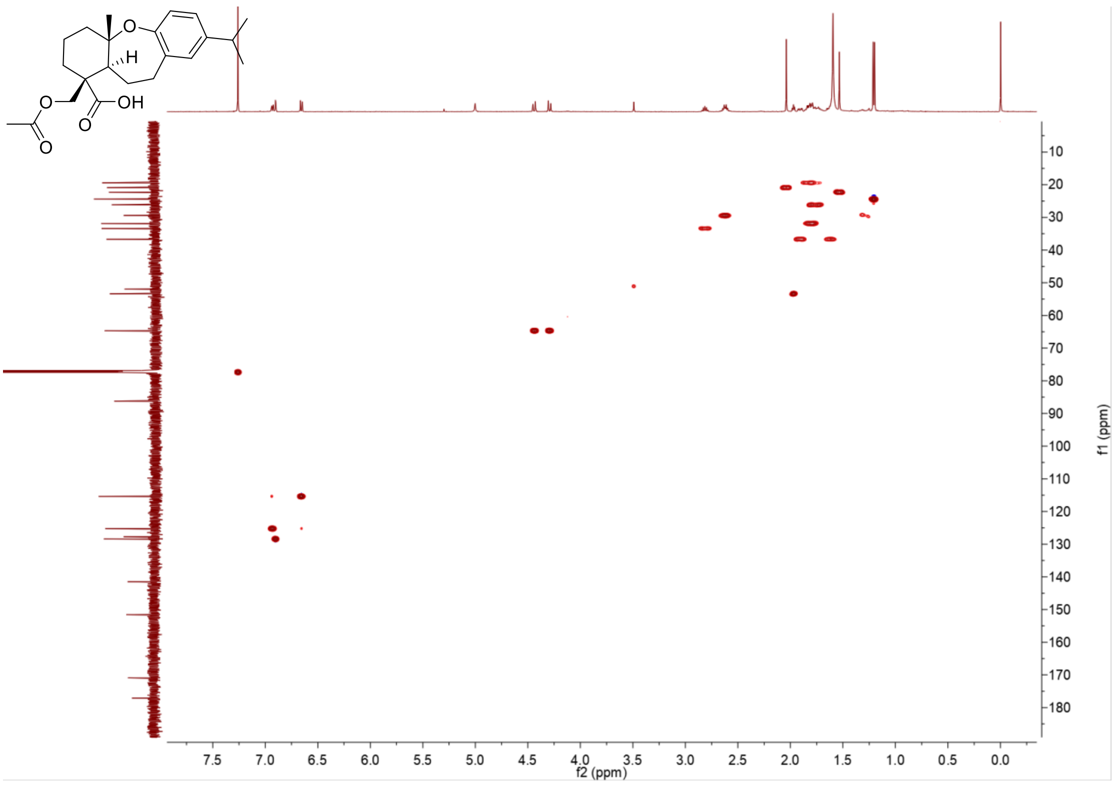

Figure S65. HSQC (600 MHz) spectrum of **6** in $CDCl_3$.

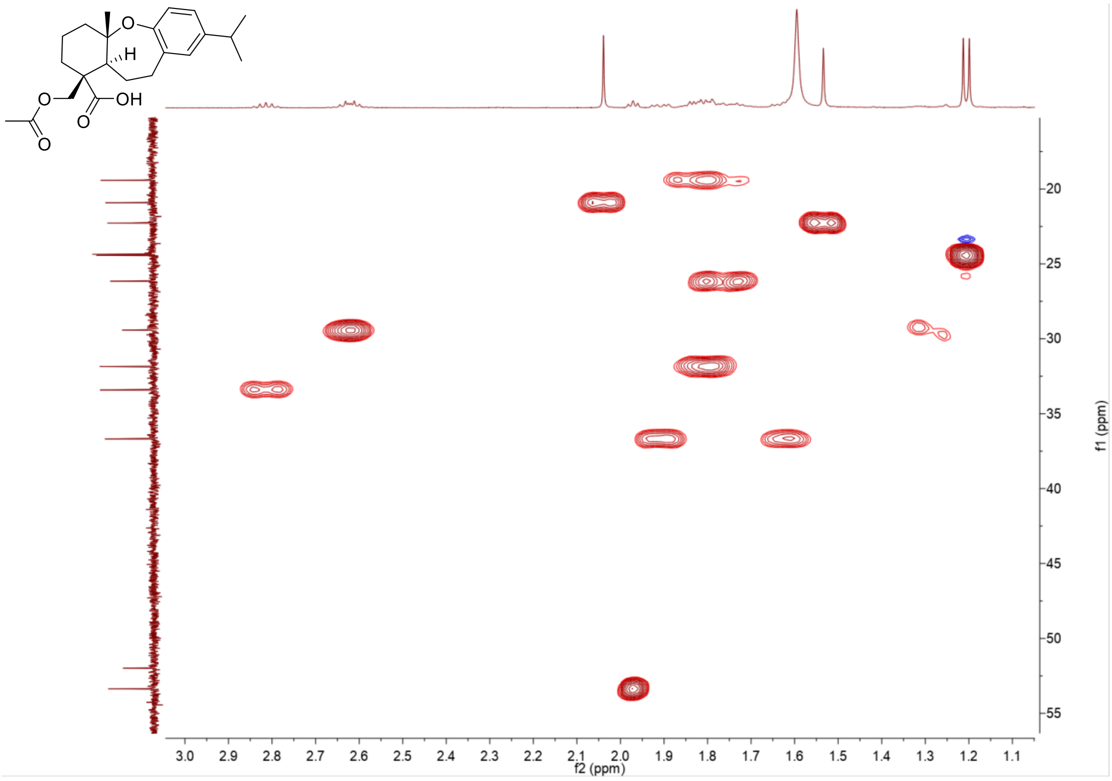

Figure S66. Enlarged HSQC (600 MHz) spectrum of **6** in $CDCl_3$.

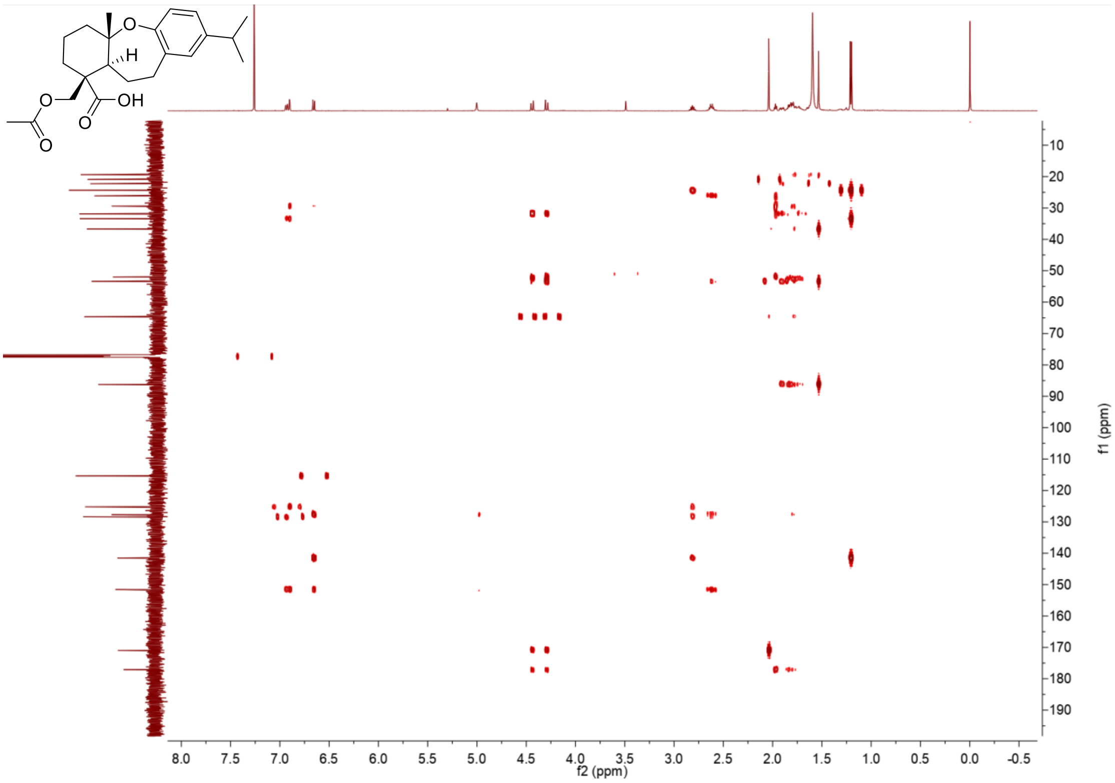

Figure S67. HMBC (600 MHz) spectrum of **6** in $CDCl_3$.

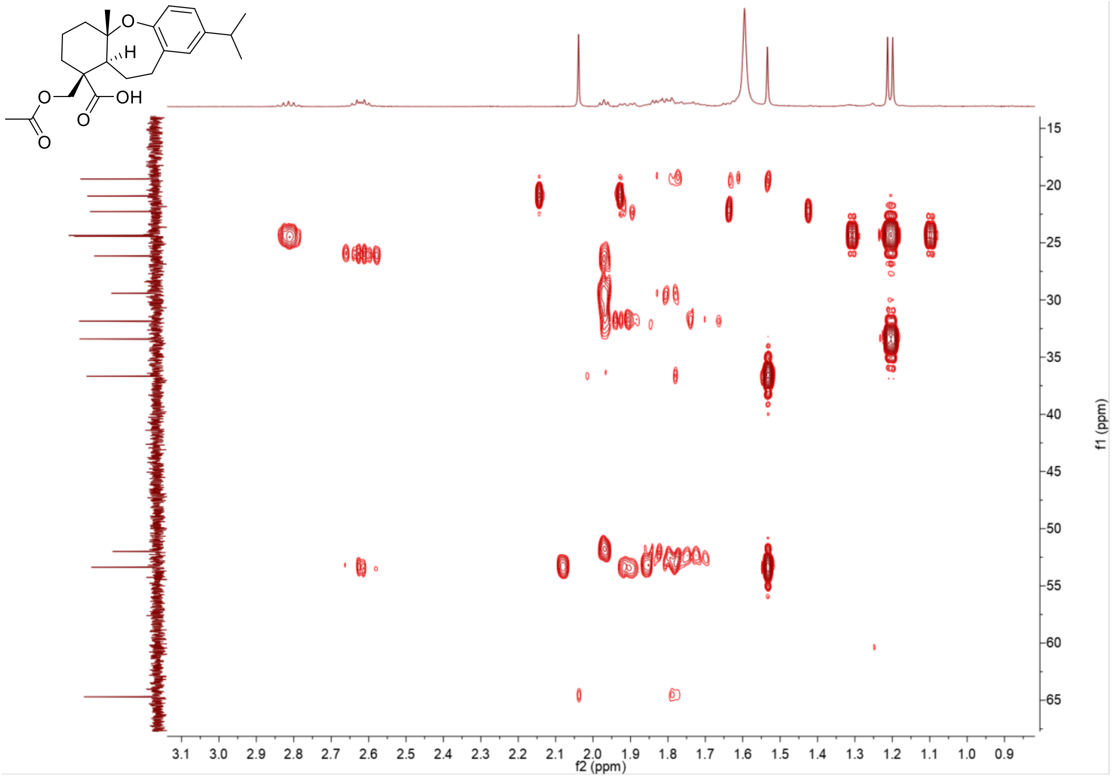

Figure S68. Enlarged HMBC (600 MHz) spectrum of **6** in $CDCl_3$.

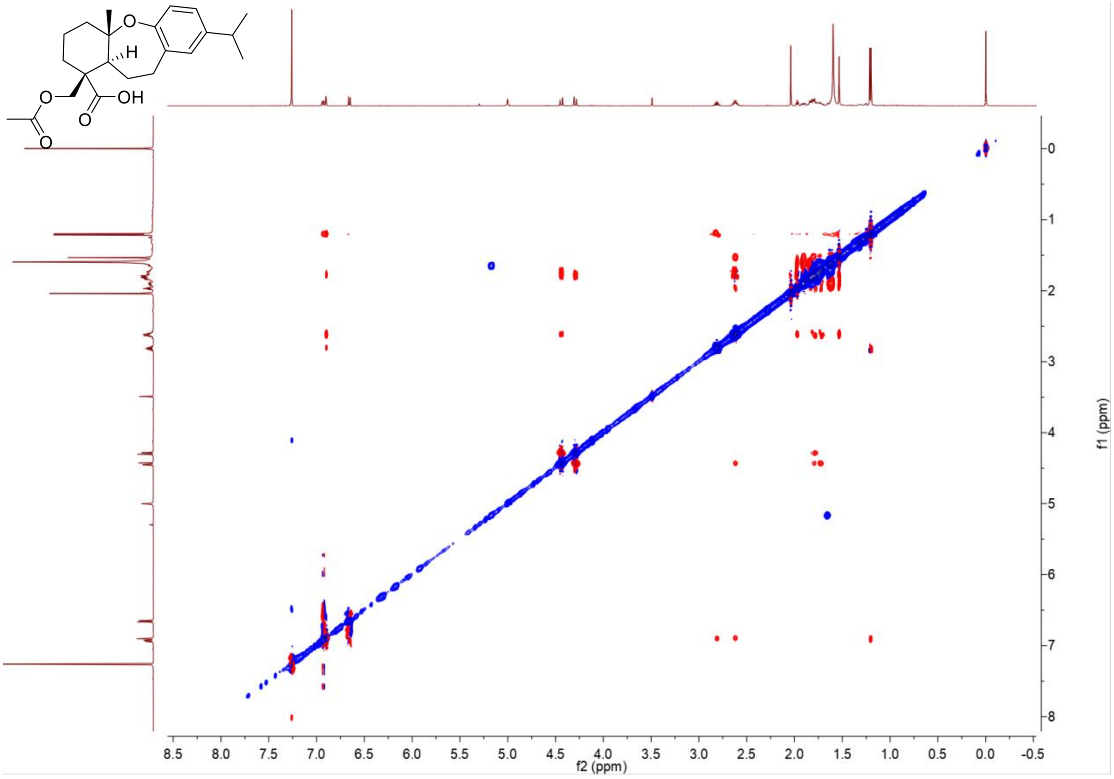

Figure S69. ROESY (600 MHz) spectrum of **6** in $CDCl_3$.

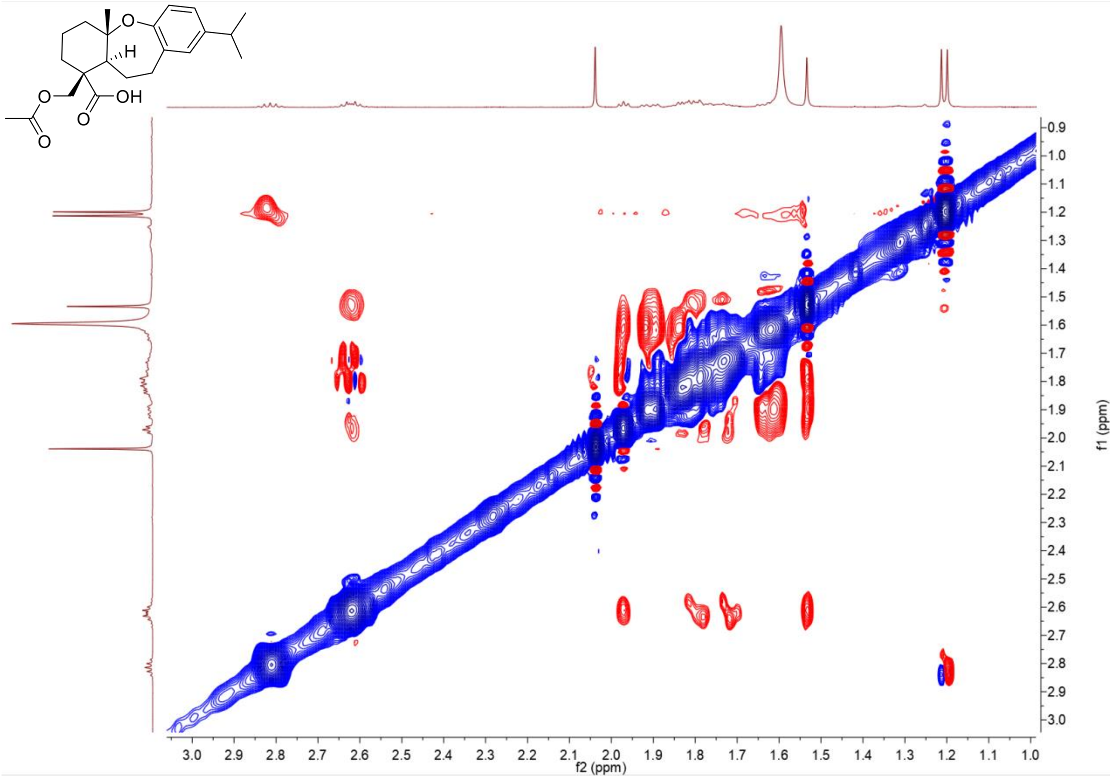

Figure S70. Enlarged ROESY (600 MHz) spectrum of **6** in $CDCl_3$.

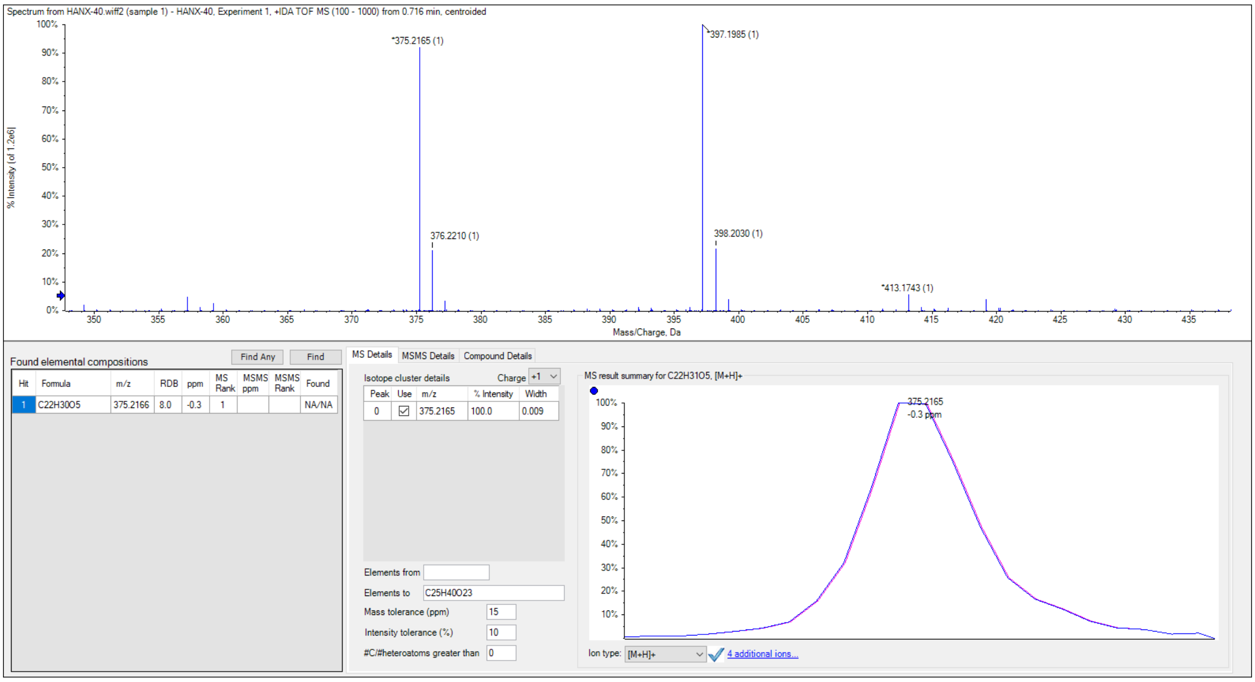

Figure S71. HRESIMS of **6**.

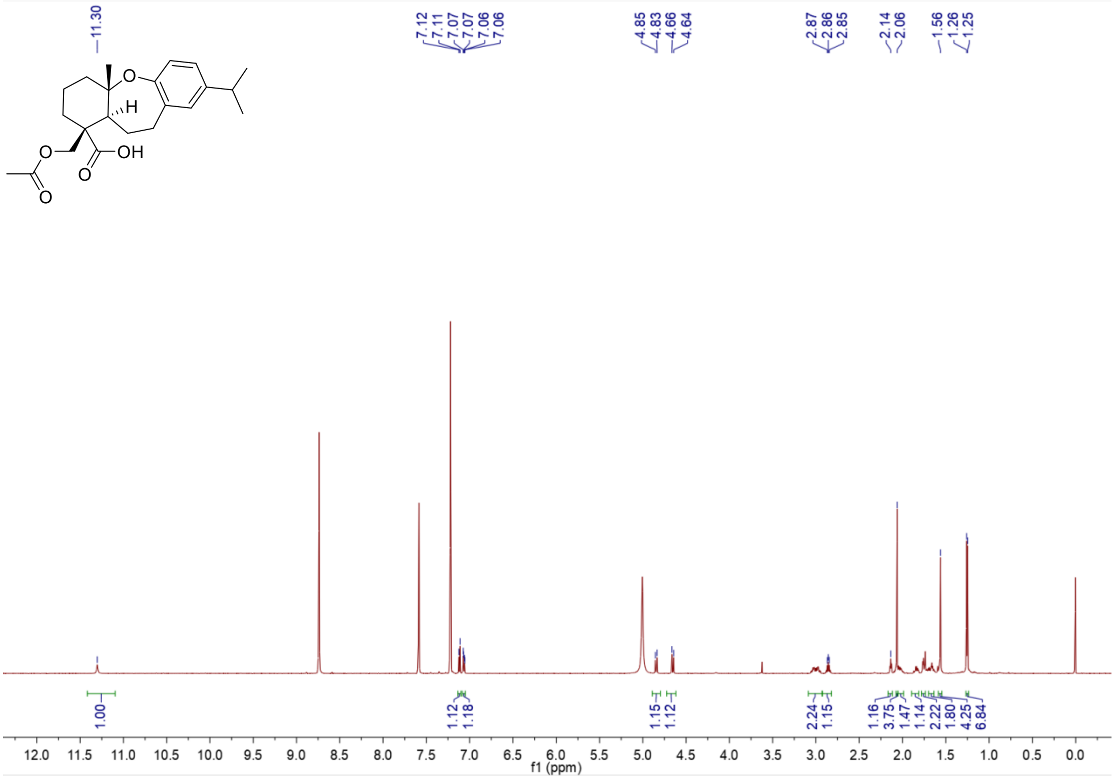

Figure S72. ^{1}H NMR (600 MHz) spectrum of **6** in pyridine-d_5.

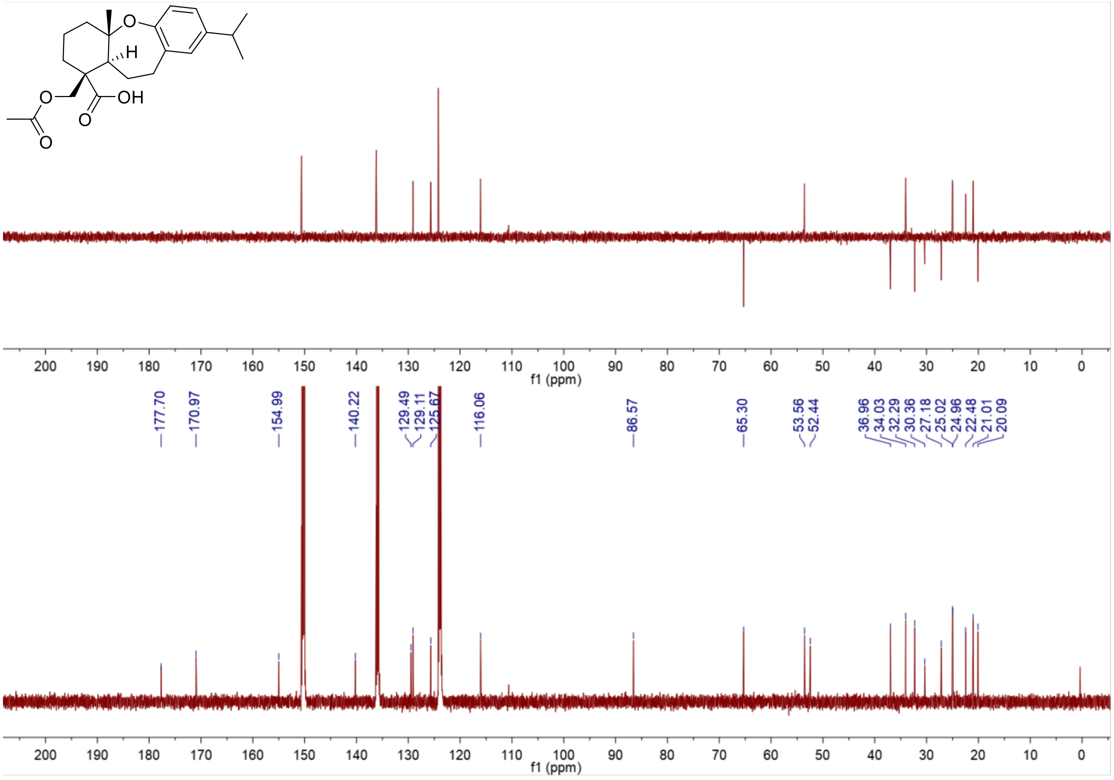

Figure S73. ^{13}C NMR and DEPT (150 MHz) spectra of **6** in pyridine-d_5.

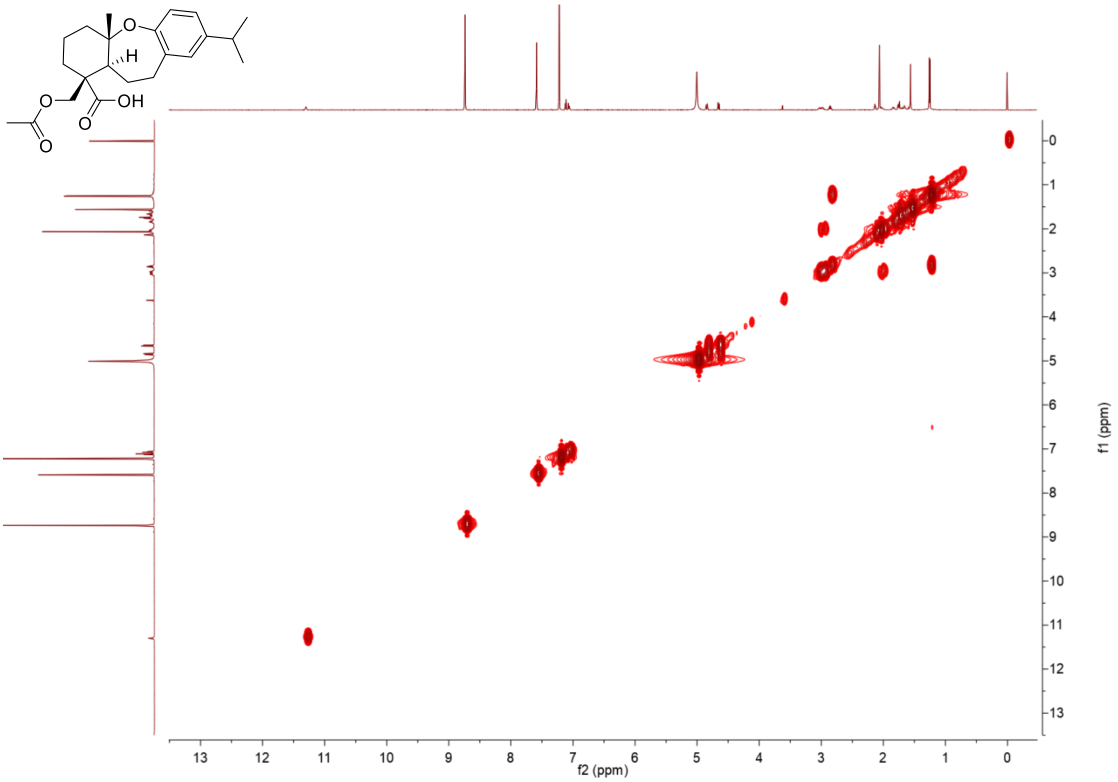

Figure S74. ^{1}H-^{1}H COSY (600 MHz) spectrum of **6** in pyridine-d_5.

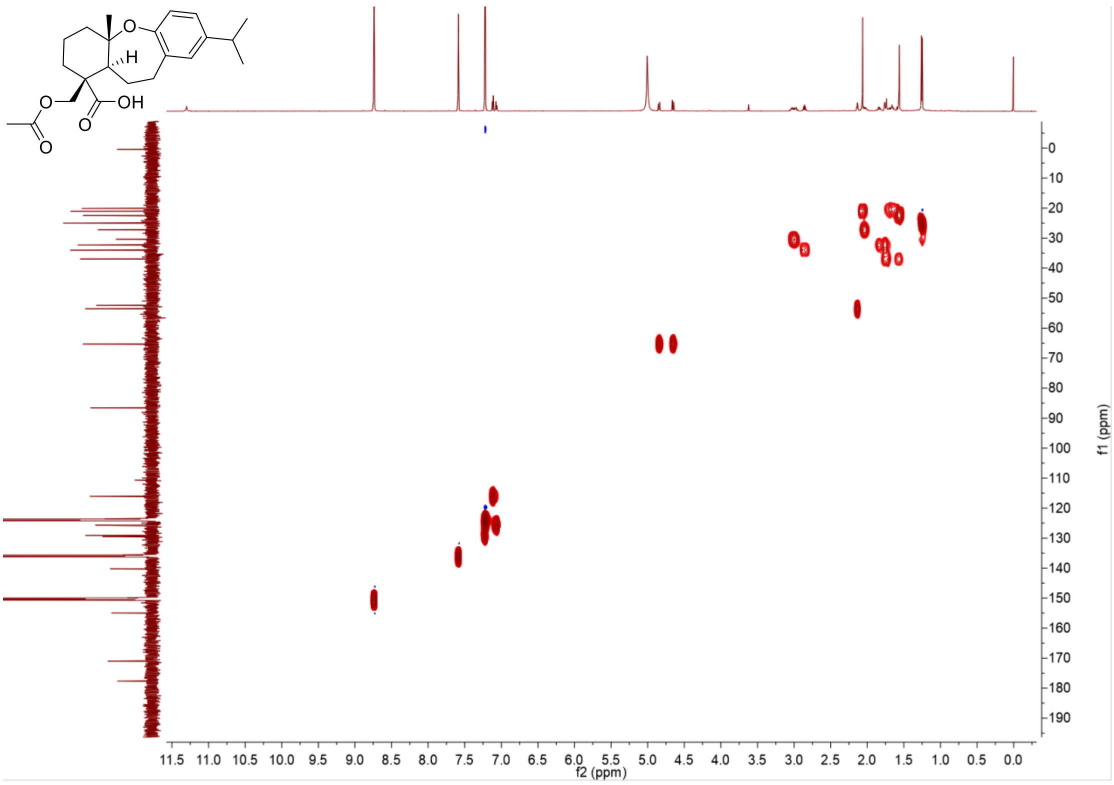

Figure S75. HSQC (600 MHz) spectrum of **6** in pyridine-d_5.

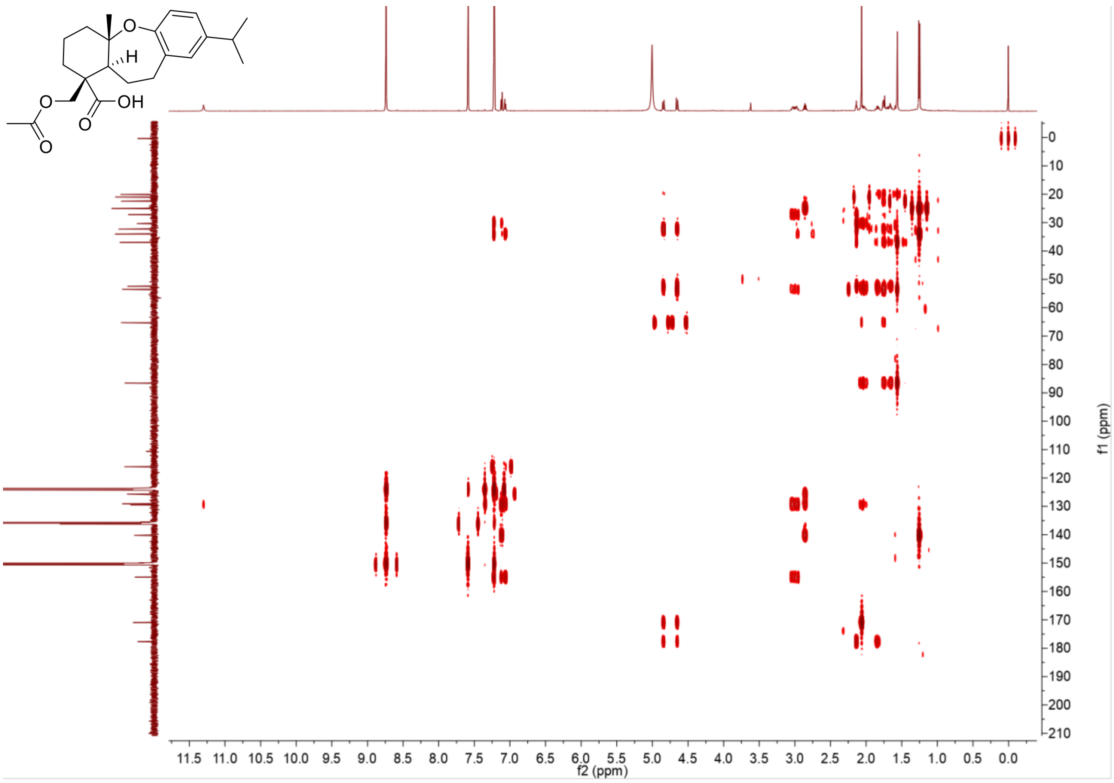

Figure S76. HMBC (600 MHz) spectrum of **6** in pyridine-d_5.

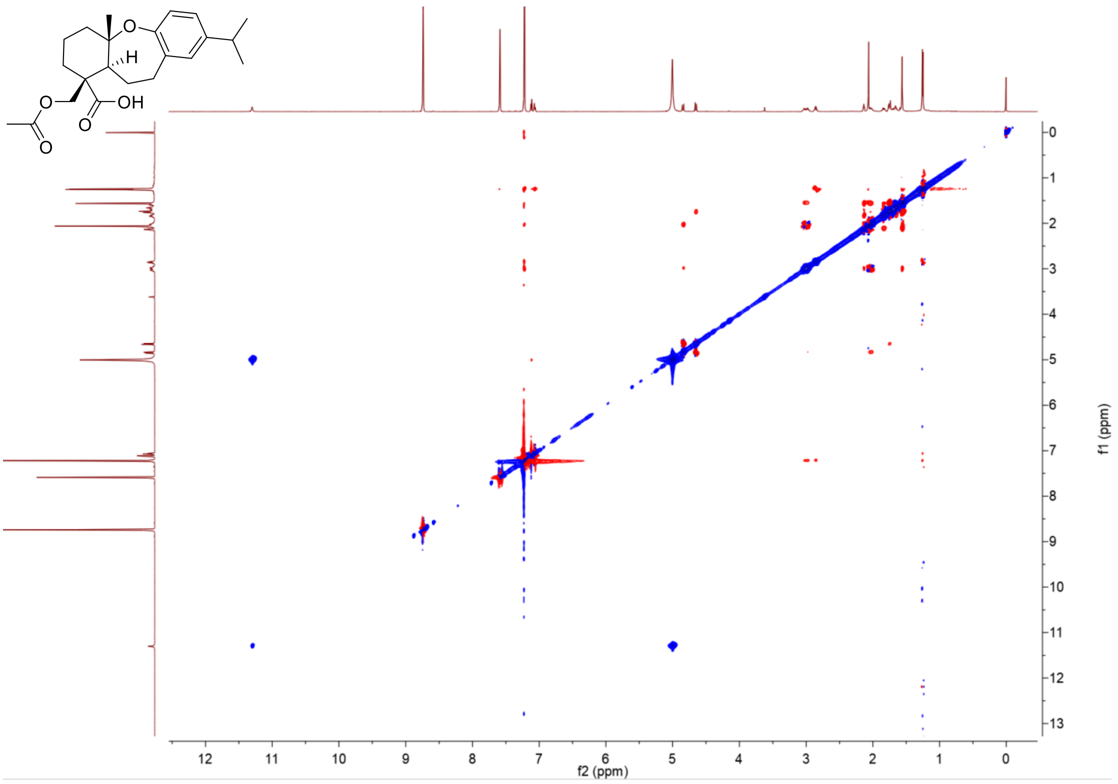

Figure S77. ROESY (600 MHz) spectrum of **6** in pyridine-d_5.

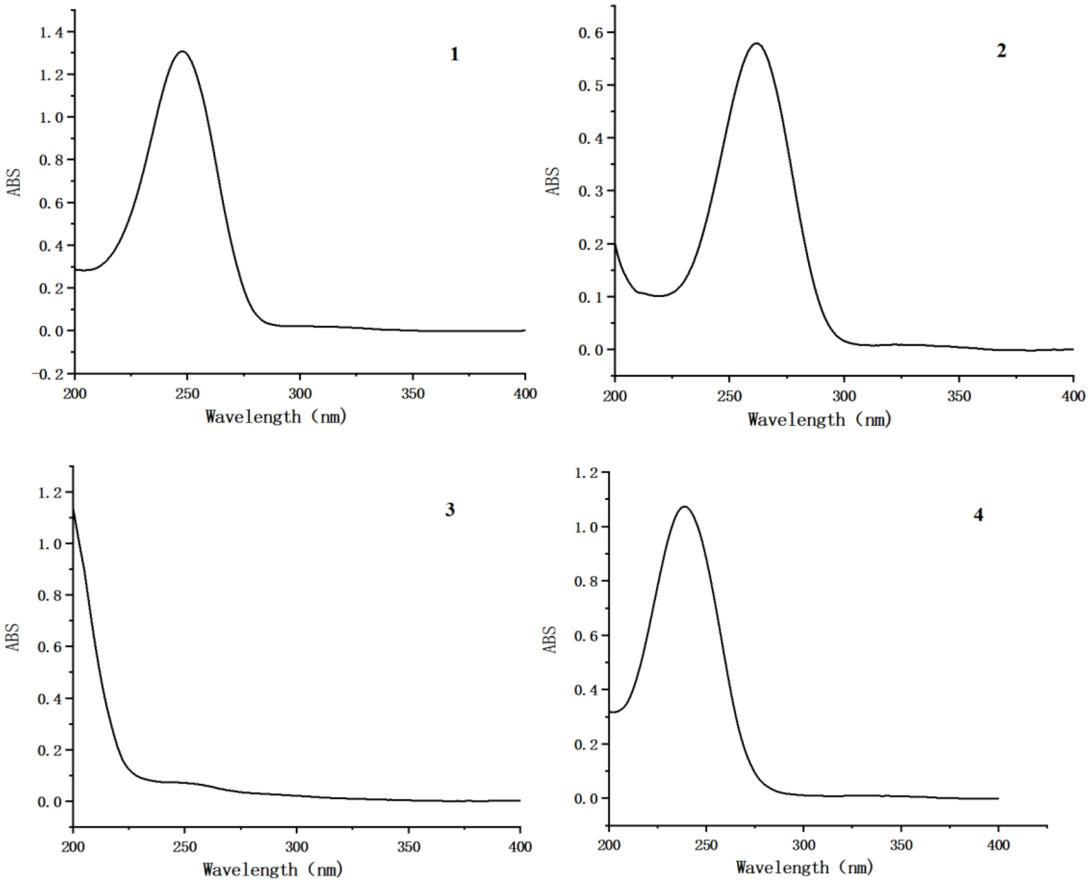

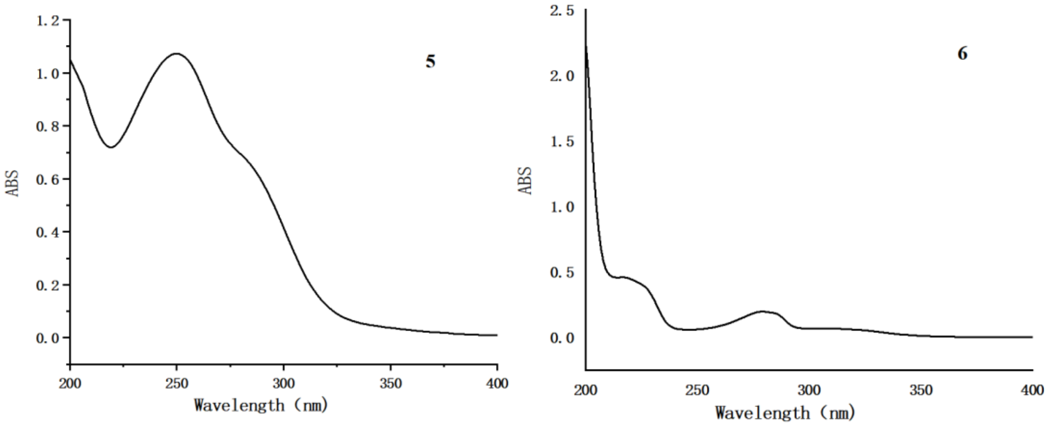

Figure S78. UV Spectra for compounds **1**–**6**.

Table S10. Crystal data and structure refinement for **1**

Identification code	1
Empirical formula	C40H56O6
Formula weight	632.84
Temperature/K	100.00(10)
Crystal system	monoclinic
Space group	P21
a/Å	6.35285(4)
b/Å	16.48619(7)
c/Å	16.53352(10)
α/°	90
β/°	95.4460(5)
γ/°	90
Volume/Å3	1723.810(16)
Z	2
ρcalcg/cm3	1.219
μ/mm-1	0.633
F(000)	688.0
Crystal size/mm3	0.15 × 0.12 × 0.1
Radiation	Cu Kα (λ = 1.54184)
2Θ range for data collection/°	5.37 to 148.784
Index ranges	-7 ≤ h ≤ 7, -20 ≤ k ≤ 20, -20 ≤ l ≤ 20
Reflections collected	32528
Independent reflections	6888 [Rint = 0.0241, Rsigma = 0.0184]
Data/restraints/parameters	6888/1/423
Goodness-of-fit on F2	1.070
Final R indexes [I>=2σ (I)]	R1 = 0.0286, wR2 = 0.0737
Final R indexes [all data]	R1 = 0.0315, wR2 = 0.0744
Largest diff. peak/hole / e Å-3	0.12/-0.15
Flack parameter	-0.03(5)

Table S11. Crystal data and structure refinement for **4**

Identification code	4
Empirical formula	C38H56O8

Formula weight	640.82
Temperature/K	99.99(10)
Crystal system	monoclinic
Space group	P21
a/Å	7.37143(4)
b/Å	12.23188(7)
c/Å	19.03670(10)
α/°	90
β/°	93.1491(5)
γ/°	90
Volume/Å3	1713.879(16)
Z	2
ρcalcg/cm3	1.242
μ/mm-1	0.687
F(000)	696.0
Crystal size/mm3	0.11 × 0.08 × 0.06
Radiation	Cu Kα (λ = 1.54184)
2Θ range for data collection/°	4.648 to 148.642
Index ranges	-9 ≤ h ≤ 9, -14 ≤ k ≤ 15, -23 ≤ l ≤ 23
Reflections collected	33252
Independent reflections	6840 [Rint = 0.0260, Rsigma = 0.0173]
Data/restraints/parameters	6840/1/443
Goodness-of-fit on F2	1.054
Final R indexes [I>=2σ (I)]	R1 = 0.0278, wR2 = 0.0742
Final R indexes [all data]	R1 = 0.0283, wR2 = 0.0746
Largest diff. peak/hole / e Å-3	0.20/-0.21
Flack parameter	0.01(3)
